# Annotated nomenclator of extant and fossil taxa of the Paludomidae (Caenogastropoda, Cerithioidea)

**DOI:** 10.3897/zookeys.850.34238

**Published:** 2019-05-27

**Authors:** Marco T. Neiber, Matthias Glaubrecht

**Affiliations:** 1 Center for Natural History (CeNak), Zoological Museum, Universität Hamburg, Martin-Luther-King-Platz 3, 20146 Hamburg, Germany Universität Hamburg Hamburg Germany

**Keywords:** Freshwater snails, Gastropoda, nomenclature, taxonomy, www-references

## Abstract

This nomenclator provides bibliographic details on all names in the family-, genus-, and species-group of the the family Paludomidae. All nomenclaturally available names are discussed including junior homonyms and objective junior synonyms as well as unavailable names such as nomina nuda, infrasubspecific names and, to some extent, also incorrect subsequent spellings. In the family-group a total of 28 names are included in the nomenclator, of which 21 are available and seven unavailable names. Of the available names in the family-group, six are invalid for nomenclatural reasons. In the genus-group a total of 57 names are included in the catalogue. Of the available names in the genus-group, 11 are invalid for nomenclatural reasons. In the species-group a total of 499 names are included, of which 463 are available, but 21 are invalid for nomenclatural reasons. All names are given in their original combination and spelling (mandatory changes are discussed and corrected spellings are provided), along with the reference to the original publication. For each family- and genus-group name, the original classification and the type genus or type species, respectively, are given. For species-group taxa the type locality and type horizon (for fossil taxa) are provided, usually as given in the original publication. A new name, *Cleopatraadami* nom. nov., is proposed for the fossil *Cleopatracylindrica* (Adam, 1957), which is a homonym of *Cleopatracridlandicylindrica* Mandahl-Barth, 1954, and a lectotype for *Cleopatradubia* Adam, 1959 is designated. A new replacement name *Leloupiella* nom. nov. is proposed for *Stormsia* Leloup, 1953 which is a homonym of *Stormsia* Bourguignat, 1891.

## Introduction

The Paludomidae Stoliczka, 1868 is currently understood as representing taxa with a general distribution including most of tropical sub-Saharan Africa and Madagascar as well as South and Southeast Asia (see Neiber and Glaubrecht 2019b who summarize the results of phylogenetic analyses presented by West and Michel 2000; Michel 2004; Wilson et al. 2004; Glaubrecht and Strong 2007; Strong et al. 2011; for a recent compilation on the diversity of family-group names available for this group and recent classifications, see also Bouchet and Rocroi 2005; Bouchet et al. 2017).

Species in this family of freshwater cerithioideans show a high diversity with regard to shell shape and ornamentation. The endemic paludomids of Lake Tanganyika have especially attracted much attention by taxonomists, early biogeographers, and evolutionary biologists because of their thalassoid (Bourguignat 1885) appearance, i.e. their resemblance to various marine gastropod families (Glaubrecht 1996, 2008; see also Glaubrecht 2009, 2011 for reviews). A large number of names have been introduced for Tanganyikan paludomids by the French malacologists J.-R. Bourguignat, the head of the so called ‘Nouvelle École’ (Dance 1970), on the basis of differences of the shell that are often hardly perceptible (Bourguignat 1885, 1888). However, being among the first to work on the gastropod fauna of the lake, several names first introduced by this author are still in use to this day, although the far greater number certainly represent individual variations that do not merit to be accepted and are thus to a large extent a burden today for taxonomists, biogeographers, and evolutionary biologists working on the paludomid fauna of Lake Tanganyika.

Although several synoptical works covering the regional diversity of Paludomidae in Africa or Asia have been published (e.g. Preston 1915b; Pilsbry and Bequaert 1927; Leloup 1953; Brown 1994; West et al. 2003), Asian members of the family are still especially in need of revision. The basic prerequisite of any taxonomic work on a specific group of organisms is the historical perspective of what is already known, or from a nomenclatural point of view, which names have already been proposed for the group in question. However, a comprehensive annotated list of paludomid names is currently unavailable. Therefore, the nomenclator presented here aims at filling this gap by providing information on all names for Recent and fossil family-, genus-, and species-group taxa introduced for Paludomidae. In addition, the present contribution is intended to facilitate future taxonomic work on Paludomidae by providing direct internet links to many important older taxonomic publications following the general outlines of the nomenclators of other groups of freshwater snails presented by Haszprunar (2014) on valvatids and Neubauer (2016) on melanopsids.

## Methods

The present nomenclator includes only taxa that are, from the current perspective, members of the Paludomidae Stoliczka, 1868 and with at least some degree of plausibility (based on shell morphology, geographical and/or stratigraphical distribution) assignable to this family. Not included are, following Glaubrecht (1996), taxa referred to the genus *Pyrgulifera* Meek, 1877 (see Meek 1871: 294) from Cretaceous deposits of North America and Europe, which have often been associated with thalassoid Paludomidae from Lake Tanganyika or that have sometimes been included in the genus *Paludomus* Swainson, 1840. In the works of Deshayes (1824–1837), Heer (1861), Morelet (1864), Sandberger (1870–1875), Neumayr (1883), Cossmann (1892, 1902), and Spijkerman et al. (2015) several fossils have been described in *Paludomus* or later attributed to *Paludomus*, which are not treated here because, in our opinion, they do not belong to the Paludomidae.

Nomenclaturally available as well as unavailable names (e.g., nomina nuda or infrasubspecific names) according to the rules of the fourth edition of the International Code of Zoological Nomenclature (ICZN 1999, hereafter in brief referred to as the “Code”) with subsequent amendments (see http://www.nhm.ac.uk/hosted-sites/iczn/code/index.jsp) are discussed following the general outline of the nomenclators of the Valvatidae Gray, 1840 (Turton and Gray 1840: 79) by Haszprunar (2014) and the Melanopsidae Adams & Adams, 1854 (Adams and Adams 1853–1854: 309) by Neubauer (2016).

The present nomenclator is divided into three parts that cover the family group, the genus group and the species group in the sense of the Code, respectively. All names in these three parts are listed in alphabetical order, except when the same family-, genus- or species-group name was introduced more than once. In this case names are ordered chronological or in cases of simultaneously published names by page precedence. The header of a section dealing with a name is the name of the taxon with mandatory changes as regulated by the Code (Art. 32 and 34). These are presented in the corrected version without mentioning. In cases where names were spelled incorrectly in the original publication, this spelling is given under ‘Original spelling’.

The status of a name that is unavailable is denoted in square brackets behind the taxon’s name. Behind a name that is either an objective junior synonym of an available name in the genus-group, an unjustified emendation or a junior homonym, its status as an objectively invalid name is denoted in square brackets. All remaining names are nomenclaturally available. However, depending on the taxonomic opinion of a researcher, such names may be taxonomically invalid names. A proposal whether a name is currently accepted or not is not made as this is, in our opinion, the task for future revisionary work. The original reference for a name is always provided. An authorship of a name deviating from the authorship of the work in which it was published, is only accepted in cases where clear evidence exists that person(s) other than the author(s) of the work, in which the name was published, were responsible for the name (Code Art. 50.1.1 and 50.1.3). In such cases, the name(s) of the person(s) responsible for the name is/are listed directly behind the name and the author(s) of the work, in which the name was published, is/are cited under ‘Original reference’. Information on type genera and type species, where relevant, are provided including references to the selection of type species (if subsequently designated). Names exclusively based on fossils are marked by a dagger. Information on type localities and type strata (for fossils only) are usually given exactly as provided in the original reference (in quotes); only in few cases also other sources are additionally provided. Additional information is given in square brackets (e.g., country, province). All other nomenclaturally relevant issues are discussed in the ‘Remarks’ under a name; however actions of a First Reviser in the sense of the Code are only discussed in cases where simultaneously published primary homonyms or different spellings of a name in the original publication are involved. Incorrect subsequent spellings are discussed under the respective correctly spelled names whenever these were noticed.

## Part 1. Family-group names

In the family-group a total of 28 names are included in the nomenclator, of which 21 are available and seven unavailable names. Of the available names in the family-group, six are invalid for nomenclatural reasons.

### Bathanaliidae Ancey, 1906

**Original reference.**Ancey (1906b: 245–246, 250).

**Type genus.***Bathanalia* Moore, 1898.

### Buccinopsidae Nicolas, 1899 [Unavailable]

**Original reference.**Nicolas (1899: 519, 525).

**Original spelling.** Buccinopsidæ Nicolas, 1899.

**Remarks.** The name is apparently not based on the genus *Buccinopsis* Conrad, 1857 (see Conrad 1857: 158) but (following Bouchet et al. 2017: 52) introduced as a descriptive name alluding to the resemblance of species from Lake Tanganyika to the Buccinidae Rafinesque, 1815 (see Rafinesque 1815: 145).

### Cancellopsidae Nicolas, 1899 [Unavailable]

**Original reference.**Nicolas (1899: 519, 525).

**Original spelling.** Cancellopsidæ Nicolas, 1899.

**Remarks.** Unavailable because the name is not based on a genus-group name. It was established as a series within Tanganikidae Nourry, 1898. Following Bouchet et al. (2017: 58) the name is interpreted as descriptive alluding to the resemblance of species from Lake Tanganyika to the Cancellariidae Forbes & Hanley, 1851 (see Forbes and Hanley 1851: 360).

### Cleopatrinae Pilsbry & Bequaert, 1927

**Original reference.**Pilsbry and Bequaert (1927: 249).

**Original spelling.** Cleopatrinæ Pilsbry & Bequaert, 1927.

**Type genus.***Cleopatra* Troschel, 1857.

**Remarks.** Elevated to family rank by Germain (1933: 30).

### Giraudidae Bourguignat, 1885 [Invalid]

**Original reference.**Bourguignat (1885a: 11, 61).

**Original spelling.** Giraudidæ Bourguignat, 1885.

**Type genus.***Giraudia* Bourguignat, 1885.

**Remarks.** Invalid (Code Art. 39) because the type genus *Giraudia* Bourguignat, 1885 is a junior homonym of *Giraudia* Foerster, 1868 (Hexapoda, see Foerster 1868: 184); see also the remarks by Bouchet et al. (2017: 113).

### Hauttecoeuridae Bourguignat, 1885

**Original reference.**Bourguignat (1885a: 10, 41).

**Original spelling.** Hauttecœuridæ Bourguignat, 1885.

**Type genus.***Hauttecoeuria* Bourguignat, 1885.

**Remarks.** Used as the name of a subfamily (with the suffix –inae) and a tribe (with the suffix –ini) by Ponder and Bouchet (2005: 249); see also the remarks by Bouchet et al. (2017: 120).

### Hilacanthidae Bourguignat, 1890 [Invalid]

**Original reference.**Bourguignat (1890: 125).

**Original spelling.**Hylacanthidae Bourguignat, 1890.

**Type genus.***Hilacantha* Ancey, 1886.

**Remarks.** Originally spelled Hylacanthidae based on *Hylacantha* which is an unjustified emendation of *Hilacantha* Ancey, 1886 as indicated by the derivation of the name by Bourguignat (1890: 126, footnote on that page). The earlier use of *Hylacantha* by Bourguignat (1888: 23–24, pl. 9) is here interpreted as an incorrect subsequent spelling as there is no evidence of an intentional change of the name. The name was introduced as a replacement name of Tiphobiidae Bourguignat, 1886 based on *Tiphobia* Smith, 1880, which is unjustifiedly treated as a homonym of *Typhobia* Pascoe, 1869 (Hexapoda; see Pascoe 1869: 279) by Bourguignat (1890: 126) (see Bouchet and Rocroi 2005: 89).

### Lavigeriidae Thiele, 1925

**Original reference.**Thiele (1925: 79).

**Type genus.***Lavigeria* Bourguignat, 1888.

**Remarks.** Used as the name of a subfamily (with the suffix –inae) by Morrison (1954: 358); see also the remarks by Bouchet et al. (2017: 138).

### Limnotrochidae Ancey, 1906

**Original reference.**Ancey (1906b: 245–246, 250).

**Type genus.***Limnotrochus* Smith, 1880.

### Littorinidopsidae Nicolas, 1899 [Unavailable]

**Original reference.**Nicolas (1899: 519, 525).

**Original spelling.** Littorinidopsidæ Nicolas, 1899.

**Remarks.** The name is not based on a genus-group name. It was established as a series within Tanganikidae Nourry, 1898 by Nicolas (1899) as Littorrinidopsidae on p. 519 and as Littorinidopsidae on p. 525. Following Bouchet and Rocroi (2005: 101) and Bouchet et al. (2017: 143; as Littoridinopsidae) the name is interpreted as descriptive alluding to the resemblance of species from Lake Tanganyika to the Littorinidae Children, 1834 (see Children 1834: 110).

### Muricopsidae Nicolas, 1899 [Unavailable]

**Original reference.**Nicolas (1899: 519, 525).

**Original spelling.** Muricopsidæ Nicolas, 1899.

**Remarks.** The name is apparently not based on the genus *Muricopsis* Bucquoy & Dautzenberg, 1882 (see Bucquoy et al. 1882: 16, 19) but (following Bouchet and Rocroi 2005: 112) introduced as a descriptive name alluding to the resemblance of species from Lake Tanganyika to the Muricidae Rafinesque, 1815 (see Rafinesque 1815: 144).

### Nassopsidae Nicolas, 1899 [Unavailable]

**Original reference.**Nicolas (1899: 519, 525).

**Original spelling.** Nassopsidæ Nicolas, 1899.

**Remarks.** The name is apparently not based on the genus-group name *Nassopsis* Smith, 1890. It was established as a series within Tanganikidae Nourry, 1898 and following Bouchet and Rocroi (2005: 113) the name is interpreted as descriptive alluding to the resemblance of species from Lake Tanganyika to the ‘Nassaidae’ Swainson, 1835 (= Nassariidae Iredale, 1916) (see Swainson 1835: 18, 20, Iredale 1916: 82); see also the remarks by Bouchet et al. (2017: 160).

### Nassopsidae Kesteven, 1903

**Original reference.**Kesteven (1903: 621, 634).

**Type genus.***Nassopsis* Smith, 1890.

**Remarks.** Following Bouchet and Rocroi (2005: 113) the name is not accepted to be available from Nicolas (1899). Used as the name of a tribe with ending –ini Bouchet and Strong in Bouchet and Rocroi (2005: 113).

### Naticidopsidae Nicolas, 1899 [Unavailable]

**Original reference.**Nicolas (1899: 519, 525).

**Original spelling.** Naticidopsidæ Nicolas, 1899.

**Remarks.** The name is not based on a genus-group name. It was established as a series within Tanganikidae Nourry, 1898. Following Bouchet and Rocroi (2005: 113) the name is interpreted as descriptive alluding to the resemblance of species from Lake Tanganyika to the Naticidae Guilding, 1834 (see Guilding 1834: 29); see also the remarks by Bouchet et al. (2017: 160–161).

### Neritopsidae Nicolas, 1899 [Unavailable]

**Original reference.**Nicolas (1899: 519, 525).

**Original spelling.** Neritopsidæ Nicolas, 1899.

**Remarks.** The name used by Nicolas (1899) is apparently not based on the genus-group name *Neritopsis* Grateloup, 1832 (see Grateloup 1832: 125–126) and interpreted as descriptive alluding to the resemblance of species from Lake Tanganyika to the Neritidae Rafinesque, 1815 (see Rafinesque 1815: 144). Not Neritopsidae Gray, 1847 (see Gray 1847: 150).

### Paludominae Stoliczka, 1868

**Original reference.**Stoliczka (1868: 206–207).

**Type genus.***Paludomus* Swainson, 1840.

**Remarks.** Elevated to family rank by Pilsbry and Bequaert (1927: 248); used as the name of a tribe (with the suffix –eae) by Wenz (1939: 703).

### Paramelaniidae Moore, 1898

**Original reference.**Moore (1898c: 315).

**Original spelling.**Paramelanidae Moore, 1898.

**Type genus.***Paramelania* Smith, 1881.

**Remarks.** Stem formation in the original spelling incorrect. Used as the name of a subfamily (with the suffix –inae) by Thiele (1925: 83) and as the name of a tribe (with the suffix –eae) by Thiele (1928: 400).

### Philopotamidae Stache, 1889

**Original reference.**Stache (1889: 107).

**Type genus.***Philopotamis* Layard, 1855.

**Remarks.** Established originally as a subfamily of Melaniidae Children, 1823 (see Children 1823: 243) despite the use of the suffix –idae (see Bouchet and Rocroi 2005: 131).

### Pyramidellopsidae Nicolas, 1899 [Unavailable]

**Original reference.**Nicolas (1899: 519, 525).

**Original spelling.** Pyramidellopsidæ Nicolas, 1899.

**Remarks.** The name is not based on a genus-group name. It was established as a series within Tanganikidae Nourry, 1898. Following Bouchet and Rocroi (2005: 148) the name is interpreted as descriptive alluding to the resemblance of species from Lake Tanganyika to the Pyramidellidae Gray, 1840 (see Gray 1840: 117, 148); see also the remarks by Bouchet et al. (2017: 211).

### Reymondiinae Bandel, 1998

**Original reference.**Bandel (1998: 273).

**Type genus.***Reymondia* Bourguignat, 1885.

### Rissopsidae Nicolas, 1899 [Unavailable]

**Original reference.**Nicolas (1899: 519, 525).

**Original spelling.** Rissopsidæ Nicolas, 1899.

**Remarks.** The name is not based on a genus-group name. It was established as a series within Tanganikidae Nourry, 1898. Following Bouchet and Rocroi (2005: 152) the name is interpreted as descriptive alluding to the resemblance of species from Lake Tanganyika to the Rissoidae Gray, 1847 (see Gray 1847: 152); see also the remarks by Bouchet et al. (2017: 217).

### Rumellidae Ancey, 1906

**Original reference.**Ancey (1906b: 245–246).

**Type genus.***Rumella* Bourguignat, 1885.

**Remarks.** Used as the name of a tribe (with the suffix –ini) by Ponder and Bouchet (2005: 249) and Bouchet and Strong in Bouchet and Rocroi (2005: 161).

### Spekiidae Ancey, 1906

**Original reference.**Ancey (1906b: 246).

**Type genus.***Spekia* Bourguignat, 1879.

**Remarks.** Used as the name of a tribe (with the suffix –ini) by Ponder and Bouchet (2005: 249) and Bouchet and Strong in Bouchet and Rocroi (2005: 153). A name Spekiinae was indicated as intentionally new by Bandel (1998: 265). This represents a subsequent use of Ancey’s name, the indication as new was in error.

### Syrnolopsidae Bourguignat, 1890

**Original reference.**Bourguignat (1890: 139).

**Original spelling.** Syrnolopsidæ Nicolas, 1899.

**Type genus.***Syrnolopsis* Smith, 1880.

**Remarks.** Used as the name of a subfamily (with the suffix –inae) by Thiele (1928: 380), as the name of a superfamily (with suffix –oidea) by Golikov and Starborogatov (1987: 27) and as the name of a tribe (with the suffix –ini) by Ponder and Bouchet (2005: 249) and Bouchet and Strong in Bouchet and Rocroi (2005: 166).

### Tanganyiciidae Nourry, 1898

**Original reference.**Nourry (1898: 302).

**Original spelling.**Tanganyikidae Nourry, 1898.

**Type genus.***Tanganyikia* Bourguignat, 1885.

**Remarks.** Corrected to Tanganyiciidae Nourry, 1898 under the Code Art. 35.4.1); interpreted here as being based (by inference) on *Tanganikia* Bourguignat, 1885 which is an unjustified emendation for *Tanganyicia* Crosse, 1881. The interpretation of Bouchet and Rocroi (2005: 167) is not followed here who attribute the name to Nicolas (1899) (with year 1898) and interpret it as not being based on a genus but rather being descriptive to include all prosobranch gastropods of Lake Tanganyika. Apparently Nicolas (1899) was aware of Nourry’s (1898) introduction of the name Tanganyikidae as evidenced by the statement ‘C’est à ce point de vue que s’était placé M. Nourry, pour mettre en évidence non seulement la nécessité de réunir toutes ces formes Thalassoïdes dans une même famille, mais encore pour faire ressortir la descendance marine de la plupart des gastéropodes du Tanganyika’ (Nicolas 1899: 511 and footnote 1 on that page) and should therefore not be regarded as responsible for the name. A name Tanganyiciinae was indicated as intentionally new by Bandel (1998: 277). This represents a subsequent use of Nourry’s name, the indication as new was in error.

### Tiphobiidae Bourguignat, 1886

**Original reference.**Bourguignat (1886: 143).

**Original spelling.**Tiphobidae Bourguignat, 1886.

**Type genus.***Tiphobia* Smith, 1880.

**Remarks.** Stem formation in the original spelling incorrect. Used as the name of a subfamily (with the suffix –inae) by Morrison (1954: 373) and as the name of a tribe (with the suffix –ini) by Ponder and Bouchet (2005: 249) and Bouchet and Strong in Bouchet and Rocroi (2005: 172); see also the remarks by Bouchet et al. (2017: 244). A name Typhobiidae was indicated as an intentionally new name by Moore (1898c: 202) based on *Typhobia* (as used by Moore 1898a–c), which is an incorrect subsequent spelling of *Tiphobia* Smith, 1880. This is interpreted to represent a subsequent use of Bourguignat’s name here and therefore the indication as new was in error. Similarly, a name Tiphobiinae was indicated as intentionally new by Bandel (1998: 262). This also represents a subsequent use of Bourguignat’s name, the indication as new was in error.

### Trochidopsidae Nicolas, 1899 [Unavailable]

**Original reference.**Nicolas (1899: 519, 525).

**Original spelling.** Trochidopsidæ Nicolas, 1899.

**Remarks.** The name is not based on a genus-group name. It was established as a series within Tanganikidae Nourry, 1898 (see Nicolas 1899). Following Bouchet and Rocroi (2005: 176) the name is interpreted as descriptive alluding to the resemblance of species from Lake Tanganyika to the Trochidae Rafinesque, 1815 (see Rafinesque 1815: 143); see also the remarks by Bouchet et al. (2017: 250–251).

### Turbinidopsidae Nicolas, 1899 [Unavailable]

**Original reference.**Nicolas (1899: 525).

**Original spelling.** Turbinidopsidæ Nicolas, 1899.

**Remarks.** The name is not based on a genus-group name. It was established as a series within Tanganikidae Nourry, 1898 (see Nicolas 1899). The name is interpreted here as descriptive alluding to the resemblance of species from Lake Tanganyika to the Turbinidae Rafinesque, 1815 (see Rafinesque 1815: 144).

## Part 2. Genus-group names

In the genus-group a total of 57 available names are included in the catalogue. Of the available names in the genus-group, 11 are invalid for nomenclatural reasons.

The genus-group name *Tanalia* Gray, 1847, type species *Neritaaculeata* Gmelin, 1791 by original designation, which has often been used as a genus-group name in Paludomidae Stoliczka, 1868, is not included in the nomenclator because its type species belongs to the Neritidae Rafinesque, 1815 (Neiber and Glaubrecht 2019a).

### *Anceya* Bourguignat, 1885

**Original reference.**Bourguignat (1885a: 10, 14–15).

**Type species.***Anceyagiraudi* Bourguignat, 1885, by monotypy.

**Remarks.** Gender: feminine.

### *Baizea* Bourguignat, 1885

**Original reference.**Bourguignat (1885a: 10, 33–34).

**Type species.***Baizeagiraudi* Bourguignat, 1885, by monotypy.

**Remarks.** Gender: feminine.

### *Bathanalia* Moore, 1898

**Original reference.**Moore (1898a: 451–452, 454–455; 1898b: 165, 174; 1898c: 189, 192, 202–203).

**Type species.***Bathanaliahowesii* Moore, 1898, by monotypy.

**Remarks.** Gender: feminine. The name *Bathanalia* was published simultaneously (March 1898) in three separate works by J. E. S. Moore (1998a–c). In Moore (1898a) only the spelling *Bathanalia* was used. In Moore (1898b) aside from the spelling *Bathanalia*, also the spellings *Bathyanalia* (p. 166, 170) and *Bathynalia* (p. 171), and in Moore (1998c) aside from *Bathanalia*, also the spelling *Batanalia*. In a subsequent work by the author, Moore (1898d), only the name *Bathanalia* was used. Therefore, Moore (1898d) is deemed here as First Reviser under the Code Art. 24.2.4 of the simultaneously published spellings of Moore (1898a–c) and *Bathanalia* is regarded as the correct original spelling.

### *Bourguignatia* Giraud, 1885 [Invalid]

**Original reference.**Giraud (1885a: 193–194).

**Type species.***Bourguignatiaimperialis* Giraud, 1885, by monotypy.

**Remarks.** Gender: feminine. Junior homonym of *Bourguignatia* Brusina, 1884 (Gastropoda, Neritidae; see Brusina 1884: 72).

### *Bridouxia* Bourguignat, 1885

**Original reference.**Bourguignat (1885a: 10, 29).

**Type species.***Bridouxiagiraudi* Bourguignat, 1885, by subsequent designation (Pilsbry and Bequaert 1927: 314).

**Remarks.** Gender: feminine.

### *Burtonilla* Smith, 1904

**Original reference.**Smith (1904: 97).

**Type species.***Turbonillaterebriformis* Smith, 1890, by monotypy.

**Remarks.** Gender: feminine.

### *Bythoceras* Moore, 1898

**Original reference.**Moore (1898a: 451–452; 1898b: 165–166, 171).

**Type species.***Bythocerasiridescens* Moore, 1898, by monotypy.

**Remarks.** Gender: neuter (Code Art. 30.1.2). The name *Bythoceras* was published simultaneously (March 1898) in two separate works by J.E.S. Moore (see Moore 1998a–b). In Moore (1898a) aside from the spelling *Bythoceras* also the spelling *Bathoceras* was used on p. 455. In Moore (1898b), only the spelling *Bythoceras* was used. In a subsequent work by the author, Moore (1898d), only the name *Bythoceras* was used. Therefore, Moore (1898d) is deemed here as First Reviser under the Code Art. 24.2.4 of the simultaneously published spellings of Moore (1898a, b) and *Bythoceras* is regarded as the correct original spelling.

### *Cambieria* Bourguignat, 1885 [Invalid]

**Original reference.**Bourguignat (1885a: 42, 78).

**Type species.***Lithoglyphusrufofilosus* Smith, 1880, by subsequent designation (Bourguignat 1890: 82).

**Remarks.** Gender: feminine. Introduced as a subgenus of *Tanganikia* Bourguignat, 1885. Objective junior synonym of *Tanganyicia* Crosse, 1881 (same type species).

### *Chytra* Moore, 1898

**Original reference.**Moore (1898d: 307).

**Type species.***Limnotrochuskirkii* Smith, 1880, by monotypy.

**Remarks.** Gender: feminine (Code Art. 30.1.2); from Greek χύτρα (= cooking pot). *Chitra* as used by Riedel (1993) is an incorrect subsequent spelling.

### *Cleopatra* Troschel, 1857

**Original reference.** Troschel (1856–1863: 100–101).

**Type species.***Cyclostomabulimoides* Olivier, 1804, by monotypy.

**Remarks.** Gender: feminine. Introduced as a subgenus of *Paludina* Férussac, 1812 (see Férussac 1812).

### *Coodea* Bourguignat, 1891

**Original reference.**Bourguignat and Servain (1891: 165).

**Type species.***Rissoaponsonbyi* Smith, 1889, by monotypy.

**Remarks.** Gender: feminine. Objective junior synonym of *Horea* Smith, 1889 (non *Horea* Bourguignat, 1888) and objective senior synonym of *Lechaptoisia* Ancey, 1894 (same type species).

### *Coulboisia* Bourguignat, 1888

**Original reference.**Bourguignat (1888: 40, 78).

**Type species.***Stanleyagiraudi* Bourguignat, 1885, by subsequent designation (Baker 1923: 174).

**Remarks.** Gender: feminine.

### *Edgaria* Bourguignat, 1888

**Original reference.**Bourguignat (1888: 34, 78).

**Type species.***Tiphobianassapaucicostata* Smith, 1881, by subsequent designation (Pilsbry and Bequaert 1927: 328).

**Remarks.** Gender: feminine.

### † *Ellinoria* Van Damme & Pickford, 2003

**Original reference.**Van Damme and Pickford (2003: 11, 19, 61–62, 66, 75, fig. 50).

**Type species.***Ellinoriamichelae* Van Damme & Pickford, 2003, by original designation.

**Remarks.** Gender: feminine.

### *Ganga* Layard, 1855

**Original reference.**Layard (1855: 88).

**Type species.***Paludomusdilatata* Reeve, 1852, by monotypy.

**Remarks.** Gender: feminine (Code Art. 30.2.3), from Sinhalese ගඟ (= river). Only the nominal species *Paludomusdilatata*Reeve 1852 was originally included in the new genus *Ganga* by Layard (1855: 88). The nominal species *Paludomusneritoides*Reeve 1847 and *Paludomusolivacea*Reeve 1847 are deemed not to be originally included under the Code Art. 67.2.5, because their assignment to the genus was doubtful as indicated by the question marks behind the names. Hence, *Paludomusdilatata*Reeve 1852 is the type species of *Ganga* Layard, 1855, by monotypy. *Gauga* as used by Cossmann (1909: 126) is an incorrect subsequent spelling.

### *Giraudia* Bourguignat, 1885 [Invalid]

**Original reference.**Bourguignat (1885a: 11, 61–62).

**Type species.***Giraudiapraeclara* Bourguignat, 1885, by subsequent designation (Pilsbry and Bequaert 1927: 311).

**Remarks.** Gender: feminine. A junior homonym of *Giraudia* Foerster, 1868 (Hexapoda, see Foerster 1868: 184). *Girandia* is an incorrect subsequent spelling by Smith (1889).

### *Hauttecoeuria* Bourguignat, 1885

**Original reference.**Bourguignat (1885a: 10, 46–48).

**Original spelling.***Hauttecœuria* Bourguignat, 1885.

**Type species.***Hauttecoeuriasoluta* Bourguignat, 1885, by subsequent designation (Germain 1908: 37).

**Remarks.** Gender: feminine.

### *Hemimitra* Swainson, 1840

**Original reference.**Swainson (1840: 199–200, 202, 340).

**Type species.***Paludomusretusa*Swainson 1840, by monotypy.

**Remarks.** Gender: feminine. Introduced as a subgenus of *Paludomus*Swainson 1840. See Neiber and Glaubrecht (2019a) for the identity of the type species.

### *Heteropoma* Benson, 1856 [Invalid]

**Original reference.**Benson (1856: 494).

**Type species.***Paludomussulcata* Reeve, 1847, by subsequent designation (Neiber and Glaubrecht 2019a).

**Remarks.** Gender: neuter (Code Art. 30.1.2), from Greek ἕτερο- (= other, different) and πῶμα, neuter (= lid). Objective junior synonym of *Philopotamis* Layard, 1855 (same type species).

### † *Heynderycxia* Van Damme & Pickford, 2003

**Original reference.**Van Damme and Pickford (2003: 11, 19, 59–62, 66–67, 75, 77, figs 49a–d, 54e).

**Type species.***Heynderycxiacolumellaris* Van Damme & Pickford, 2003, by original designation.

**Remarks.** Gender: feminine.

### *Hilacantha* Ancey, 1886 [Invalid]

**Original reference.**Ancey (1886: 292–293).

**Type species.***Tiphobiahorei* Smith, 1880, by monotypy for the replaced name *Tiphobia* Smith, 1880.

**Remarks.** Gender: feminine (Code Art. 30.1.2), from Greek ὕλη- (= wooden) and ἄκανθα, feminine (= thorn, spine). Unnecessary nom. nov. pro *Tiphobia* Smith, 1880. *Hylacantha* (Bourguignat 1888: 23–24, pl. 9) is an incorrect subsequent spelling, *Hylacantha* (Bourguignat 1890: 126) is an unjustified emendation.

### *Hirthia* Ancey, 1898

**Original reference.**Ancey (1898: 142).

**Type species.***Hirthialittorina* Ancey, 1898, by subsequent designation (Pilsbry and Bequaert 1927: 328).

**Remarks.** Gender: feminine. *Hirtia* as used by Van Damme and Pickford (2003) is an incorrect subsequent spelling.

### *Horea* Bourguignat, 1888

**Original reference.**Bourguignat (1888: 27–28).

**Type species.***Horeatanganikana* Bourguignat, 1888, by monotypy.

**Remarks.** Gender: feminine.

### *Horea* Smith, 1889 [Invalid]

**Original reference.**Smith (1889: 175).

**Type species.***Rissoaponsonbyi* Smith, 1889, by monotypy.

**Remarks.** Gender: feminine. Established as a subgenus of *Rissoa* Desmarest, 1814 (Desmarest 1814: 7). Invalid, junior homonym of *Horea* Bourguignat, 1888. The name *Horea* was introduced independently by Bourguignat (1888) and Smith (1889) for different taxa in honour of Captain Edward Coode Hore.

### *Hylacantha* Bourguignat, 1890 [Invalid]

**Original reference.**Bourguignat (1890: 126).

**Remarks.** Gender: feminine, see Remarks under *Hilacantha* Ancey, 1886. Unjustified emendation of *Hilacantha* Ancey, 1886 as evidenced by the derivation of the name by Bourguignat (1890: 126, footnote on that page), see also Martens (1891: 7–8). The earlier use of *Hylacantha* by Bourguignat (1888: 23–24, pl. 9) is here interpreted as an incorrect subsequent spelling of *Hilacantha* Ancey, 1886 as there is no evidence of an intentional change of the name. *Tylacanæha* (Nicolas 1899: 512) is here also regarded as an incorrect subsequent spelling, although hardly recognizable anymore, of *Hylacantha* Bourguignat, 1890. The work of Nicolas (1899) contains numerous typesetting errors and apparently that author was aware of the name *Tiphobia* introduced by Smith (1880a) and its replacement name *Hilacantha* introduced by Ancey (1886) in the emendated form *Hylacantha* by Bourguignat (1890) as suggested by the statement “L’énumeration se continue par un genre des plus caracteristques, *Tiphobia* ou *Tylacanæha*, qui par l’opercule est une Melanidæ, tandis que l’absence d’épiderme l’en sépare, d’eû une grande indecision pour cette espèce” (Nicolas 1899: 512) in combination with the synonymous usage of the names *Hylacanthalongirostris* and *Tiphobialongirostris* (Nicolas 1899: 515).

### *Joubertia* Bourguignat, 1888

**Original reference.**Bourguignat (1888: 32, 78).

**Type species.***Paramelaniabaizeana* Bourguignat, 1885, by subsequent designation (Pilsbry and Bequaert 1927: 324).

**Remarks.** Gender: feminine.

### *Lavigeria* Bourguignat, 1888

**Original reference.**Bourguignat (1888: 31, 33–34, 78).

**Type species.***Tiphobianassagrandis* Smith, 1881, by subsequent designation (Pilsbry and Bequaert 1927: 324).

**Remarks.** Gender: feminine. *Lavigieria* is an incorrect subsequent spelling by Martens (1891: 9).

### *Lechaptoisia* Ancey, 1894 [Invalid]

**Original reference.**Ancey (1894: 29).

**Type species.***Rissoaponsonbyi* Smith, 1889, by typification of replaced name.

**Remarks.** Gender: feminine. Nom. nov. pro *Horea* Smith, 1889. Objective junior synonym of *Coodea* Bourguignat, 1891 (same type species).

### *Leloupiella* nom. nov.

http://zoobank.org/17726764-61CF-4026-905E-7149C3B1E6EF

**Type species.***Giraudiaminima* Smith, 1908, by typification of the replaced name.

**Remarks.** New replacement name for the preoccupied name *Stormsia* Leloup, 1953 (not *Stormsia* Bourguignat, 1891). See also Remarks under *Stormsia* Bourguignat, 1891 and *Stormsia* Leloup, 1953.

### *Limnotrochus* Smith, 1880

**Original reference.**Smith (1880b: 425).

**Type species.***Limnotrochusthomsoni* Smith, 1880, by subsequent designation (Pilsbry and Bequaert 1927: 318).

**Remarks.** Gender: masculine.

### *Martelia* Dautzenberg, 1908

**Original reference.**Dautzenberg (1908: 328).

**Type species.***Marteliatanganyicensis* Dautzenberg, 1908, by monotypy.

**Remarks.** Gender: feminine.

### *Mysorelloides* Leloup, 1953

**Original reference.**Leloup (1953: 25, 87–92, 115, 232, pI. 5, fig. 3, figs 11, 41, 42, 57K, 62, 72CC)

**Type species.***Bithyniamultisulcata* Bourguignat, 1888, by monotypy.

**Remarks.** Gender: feminine, originally combined with a feminine adjectival species-group name (Code Art. 30.1.4.4).

### *Nassopsidia* Martens, 1897

**Original reference.**Martens (1897: 208).

**Type species.***Paramelaniacrassilabris* Bourguignat, 1885, by subsequent designation (Pilsbry and Bequaert 1927: 328).

**Remarks.** Gender: feminine. Introduced as a subgenus of *Paramelania* Smith, 1881.

### *Nassopsis* Smith, 1890 [Invalid]

**Original reference.**Smith (1890: 93).

**Type species.***Tiphobianassagrandis* Smith, 1881, by original designation.

**Remarks.** Gender: feminine (Code Art. 30.1.2), from Latin *nassa*, feminine (= weel) and Greek ὄψις, feminine (= appearance). Objective junior synonym of *Lavigeria* Bourguignat, 1888 (same type species).

### *Odontochasma* Tomlin, 1930

**Original reference.**Tomlin (1930: 23).

**Type species.***Tanaliastomatodon* Benson, 1862, by monotypy for the replaced name *Stomatodon* Benson, 1862.

**Remarks.** Gender: neuter (Code Art. 30.1.1). Nom. nov. pro *Stomatodon* Benson, 1862 (non *Stomatodon* Seeley, 1861).

### *Paludomus* Swainson, 1840

**Original reference.**Swainson (1840: 198–201, 210, 340).

**Type species.***Melaniaconica* Griffith & Pidgeon, 1833 in Griffith and Pidgeon (1833–1834) (non *Melaniaconica* Say, 1821, see Say 1821: 176–177), by subsequent designation (Gray 1847: 155).

**Remarks.** Gender: feminine (Code Art. 30.1.1), from Latin palus, feminine (= swamp) and domus, feminine (= house). Swainson (1840) also combined the name with feminine adjectival species-group names. *Paludimus* as used by Kobelt (1909) is an incorrect subsequent spelling.

### *Paramelania* Smith, 1881

**Original reference.**Smith (1881b: 559).

**Type species.***Tiphobiadamoni* Smith, 1881, by subsequent designation (Bourguignat 1890: 162).

**Remarks.** Gender: feminine. Introduced as a subgenus of *Tiphobia* Smith, 1880. The subsequent designation of the same taxon as type species of *Paramelania* Smith, 1881 by Pilsbry and Bequaert (1927: 320) is invalid. *Paramellania* as used by Moore (1903) is an incorrect spelling.

### *Philopotamis* Layard, 1855

**Original reference.**Layard (1855: 88–89).

**Type species.***Paludomussulcata* Reeve, 1847, by subsequent designation (Nevill 1884: 297).

**Remarks.** Gender: feminine (Code Art. 30.1.3). Cossmann (1909: 126) gave as type species *Philopotamisregalis* Layard, 1855; this act is invalid.

### *Ponsonbya* Ancey, 1890

**Original reference.**Ancey (1890: 347).

**Type species.***Ponsonbyaleucoraphe* Ancey, 1890, by monotypy.

**Remarks.** Gender: feminine.

### *Potadomoides* Leloup, 1953

**Original reference.**Leloup (1953: 102–105).

**Type species.***Potadomoidespelseneeri* Leloup, 1953, by monotypy.

**Remarks.** Gender: masculine (Code Art. 30.1.4.4).

### *Pseudocleopatra* Thiele, 1928

**Original reference.**Thiele (1928: 394).

**Type species.***Pseudocleopatratogoensis* Thiele, 1928, by monotypy.

**Remarks.** Gender: feminine.

### *Randabelia* Bourguignat, 1888

**Original reference.**Bourguignat (1888: 31, 78).

**Type species.***Paramelaniahamyana* Bourguignat, 1885, by subsequent designation (Pilsbry and Bequaert 1927: 327).

**Remarks.** Gender: feminine.

### *Reymondia* Bourguignat, 1885

**Original reference.**Bourguignat (1885a: 11, 61–62).

**Type species.***Melaniahorei* Smith, 1880, by subsequent designation (Pilsbry and Bequaert 1927: 312).

**Remarks.** Gender: feminine. Attributed to Giraud, in litt. but J.-R. Bourguignat alone was responsible for the name.

### *Rivulina* Lea & Lea, 1851

**Original reference.**Lea and Lea (1851: 196–197).

**Type species.***Melaniamodicella* Lea & Lea, 1851, by subsequent designation (Neiber and Glaubrecht 2019a).

**Remarks.** Gender: feminine.

### *Rumella* Bourguignat, 1885

**Original reference.**Bourguignat (1885a: 11, 89–90).

**Type species.***Rumellagiraudi* Bourguignat, 1885, by subsequent designation (Baker 1923: 174).

**Remarks.** Gender: feminine.

### *Spekia* Bourguignat, 1879

**Original reference.**Bourguignat (1879: 27–28).

**Type species.***Lithoglyphuszonatus* Woodward, 1859, by monotypy.

**Remarks.** Gender: feminine. *Spekea* as used by Hoyle (1886: 88) and Martens (1891: 8) are incorrect subsequent spellings.

### *Stanleya* Bourguignat, 1885

**Original reference.**Bourguignat (1885a: 11, 86–87).

**Type species.***Lithoglyphusneritinoides* Smith, 1880, by original designation.

**Remarks.** Gender: feminine. The name *Stanleyaneritoides* as used by Bourguignat (1885, 1888) has to be interpreted as an incorrect subsequent spelling of the name of the nominal taxon *Lithoglyphusneritinoides* Smith, 1880 and should not be regarded (as e.g. by Ancey 1906b: 254 or Baker 1923: 174) as a newly introduced name by Bourguignat (1888) (although the author attributed the taxon to himself in that publication) because Bourguignat (1885: 87, footnote) explicitly referred to Smith (1880: 426) and Smith (1881: 287) and also used the misspelled name *Lithoglyphusneritoides* in the synonymy when first using the name *Stanleyaneritoides*. Therefore, *Stanleya* is here interpreted as not having been introduced again by Bourguignat (1888) and the fixation of *Stanleyaneritoides* with author Bourguignat (1888) by Baker (1923: 174) is not valid.

### *Stomatodon* Benson, 1862 [Invalid]

**Original reference.**Benson (1862: 414).

**Type species.***Tanaliastomatodon* Benson, 1862, by monotypy.

**Remarks.** Gender: masculine (Code Art. 30.1.2), from Greek στόμα, neuter (= mouth) and ὀδών, masculine (= tooth). The name *Stomatodon* Benson, 1862 was conditionally proposed by Benson (1862: 414). Benson (1862: 414) stated: “Unfortunately, all the specimens were deficient in the operculum, which, when examined, may possibly authorize its transfer to a new genus, in which case the specific name may fairly be employed to designate it.” And further below on the page and on the next page: “*Stomatodon* seems partly to supply one of the absent links, inasmuch as its operculum must necessarily be provided with a basal projection, while its constriction is also likely to be unguiculate.” *Stomatodon* Benson, 1862 is, however, a junior homonym of *Stomatodon* Seeley, 1861 (see Seeley 1861: 293; Gastropoda, Ringiculidae Philippi, 1853, see Philippi 1853: 190). It has been replaced by *Odontochasma* Tomlin, 1930 (see Tomlin 1930: 23 and Neiber and Glaubrecht 2019a).

### *Stormsia* Bourguignat, 1891

**Original reference.**Bourguignat and Servain (1891: 165).

**Type species.***Syrnolopsiscarinifera* Smith, 1889, by monotypy.

**Remarks.** Gender: feminine. See also Remarks under *Stormsia* Leloup, 1953.

### *Stormsia* Leloup, 1953 [Invalid]

**Original reference.**Leloup (1953: 217–219).

**Type species.***Giraudiaminima* Smith, 1908, by monotypy.

**Remarks.** Gender: feminine. *Stormsia* Leloup, 1953 is a junior homonym of *Stormsia* Bourguignat, 1891. *Stormsia* Bourguignat, 1891 was made available in a note in a biographic and bibliographic work (Servain and Bourguignat 1891) and has been overlooked by most subsequent authors. Although *Stormsia* Bourguignat, 1891 has, to our knowledge, not been used as an accepted name since its introduction, it had been mentioned by Pilsbry and Bequaert (1927) and Germain (1919b) in the synonymy of *Syrnolopis* Smith, 1880. Both, Bourguignat and Leloup apparently dedicated their generic names to Émile Pierre Joseph Storms who established a European presence in the Lake Tanganyika region. Leloup (1953) explicitly introduced *Stormsia* as a new generic name for the single nominal taxon *Giraudiaminima* Smith, 1908, while Bourguignat in Bourguignat and Servain (1891) had introduced *Stormsia* for the nominal taxon *Syrnolopsiscarinifera* Smith, 1889. The name *Stormsia* with direct reference to Leloup (1953) has been used by Brown (1980, 1994), Bandel (1998, 2006), West et al. (2003), Robertson (2007), Glaubrecht (2008), Wagner et al. (2009), and Neiber and Glaubrecht (2019b). The name *Stormsiaminima* (Smith, 1908) has been used by Brown and Mandahl-Barth (1987), Glaubrecht (1994, 1996), Baillie and Groombridge (1996), Alin et al. (1999), Irvine and Donohue (1999), Allison et al. (2000), West and Michel (2000), Van Damme and Pickford (2003), Glaubrecht and Strong (2004), Wilson et al. (2004), Levêque et al. (2006), Strong and Glaubrecht (2007), Avanesyan (2008), Strong and Glaubrecht (2008), Fermon (2009), Nicayenzi et al. (2010), and Brown (2014), but the generic name was unfortunately not referenced by these authors and is therefore, although probable, not without doubt attributable to Leloup (1953). Thus, the conditions of the Code (Art 23.9.1.1 and Art. 23.9.1.2) are, in our opinion, not met. The new replacement name *Leloupiella* nom. nov. is therefore proposed to honour Eugène Leloup’s contributions to the systematics of the malacofauna of Lake Tanganyika; gender: feminine.

### *Syrnolopsis* Smith, 1880

**Original reference.**Smith (1880b: 217–219).

**Type species.***Syrnolopsislacustris* Smith, 1880, by monotypy.

**Remarks.** Gender: feminine, see Remarks under *Nassopsis* Smith, 1890. *Syznolopsis* (Nicolas 1899: 510) is an incorrect subsequent spelling.

### † *Taeniodomus* Krause, 1897

**Original reference.** Krause (1887: 209, 213–214).

**Type species.***Taeniodomusgracilis* Krause, 1897, by subsequent designation (Cossmann 1898: 105).

**Remarks.** Gender: feminine, see Remarks under *Paludomus* Swainson, 1840.

### *Tanganikia* Bourguignat, 1885 [Invalid]

**Original reference.**Bourguignat (1885a: 10, 41–42).

**Remarks.** Gender: feminine. *Tanganikia* Bourguignat, 1885 is an unjustified emendation of *Tanganyicia* Crosse, 1881. *Tanganikea* as used by Martens (1891: 8) is an incorrect subsequent spelling.

### *Tanganyicia* Crosse, 1881

**Original reference.**Crosse (1881: 123–124).

**Type species.***Lithoglyphusrufofilosus* Smith, 1880, by original designation.

**Remarks.** Gender: feminine. *Tanganyikia* as used by Moore (1899) and Germain (1908) are incorrect subsequent spellings.

### *Tiphobia* Smith, 1880

**Original reference.**Smith (1880a: 348).

**Type species.***Tiphobiahorei* Smith, 1880, by monotypy.

**Remarks.** Gender: feminine. *Typhobia* is an incorrect subsequent spelling by Moore (1898a–c) and *Thiphobia* by Riedel (1993).

### *Vinundu* Michel, 2004

**Original reference.**Michel (2004: 2, 4).

**Type species.***Vinunduwestae* Michel, 2004, by original designation.

**Remarks.** Gender: feminine (Code Art. 30.2.2). *Vinundu* Michel & Todd, 2003 in West et al. (2003: 76–77) is unavailable because the work includes a disclaimer stating “this publication is not deemed to be valid for formal taxonomic/nomenclatural purposes”. The name was also used by Van Damme and Pickford (2003: 2) as a nomen nudum because no diagnosis, definition, or description was given.

### *Zanguebaria* Fischer, 1881

**Original reference.**Fischer (1880–1887: 2, 4).

**Type species.***Melaniaamaena* Morelet, 1851, by subsequent designation (Pilsbry and Bequaert 1927: 288).

**Remarks.** Gender: feminine.

## Part 3. Species-group names

In the species-group a total of 499 names are included, of which 463 are available but 21 are invalid for nomenclatural reasons.

### *Paludomusabbreviata* Reeve, 1852

**Original reference.**Reeve (1852: 127).

**Original spelling.***Paludomusabbreviatus* Reeve, 1852.

**Type locality.** “Ceylon” [Sri Lanka].

### *Paludomusacuta* Reeve, 1852

**Original reference.**Reeve (1852: 127).

**Original spelling.***Paludomusacutus* Reeve, 1852.

**Type locality.** “Near Pondicherry” [India, near Puducherry].

### † *Cleopatraadami* nom. nov.

http://zoobank.org/BE605896-D974-47AA-8C40-31CB9AF92C38

**Type locality.** “Rive du ravin de Nyakasia, 65 à 80 m sur lac” [Uganda, river of Nyakasia Ravine, 65 to 80 m above Lake Albert].

**Type stratum.** “Pleistocène inférieur, Série de Kaiso” [Lower Pleisocene, Kaiso Series].

**Type material.** Holotype, Musée royal de l’Afrique centrale, Tervuren MC 2780 (Adam 1957, pl. 4, fig. 1).

**Remarks.** The species was classified as *Cleopatracylindrica* (Adam, 1957) by Van Damme and Pickford (2003). This name is a secondary junior homonym of *Cleopatracridlandicylindrica* Mandahl-Barth, 1954 and because no junior synonyms exist, the new replacement name *Cleopatraadami* nom. nov. is introduced here (see also Remarks under *Viviparuscylindricus* Adam, 1957). The replacement name is chosen in honor of William Adam, the former Director of the Malacology Department of the Royal Belgian Institute of Natural Sciences, who was the first to describe the species (Adam 1957).

### *Anceyaadmirabilis* Bourguignat, 1889

**Original reference.**Bourguignat (1889: 119–120, pl. 7, figs 10, 11).

**Type locality.** “Entre Mpala et Mlilo” [Democratic Republic of the Congo, between Mpala and Mlilo (= Moliro), Lake Tanganyika].

### *Paludomusadustus* Brot, 1880 [Unavailable]

**Original reference.**Brot (1880: 25).

**Remarks.** Attributed to Swainson; published in the synonymy of *Paludomusstephanus* Benson, 1836; unavailable (Code Art. 11.6).

### *Melaniaaegyptiaca* Reeve, 1860

**Original reference.**Reeve (1860: pl. 34 fig. 227 and explanation of the plate).

**Original spelling.***Melania Ægyptiaca* Reeve, 1860.

**Type locality.** “Egypt”.

### *Bithyniabulimoidesaegyptiaca* Frauenfeld, 1864

**Original reference.**Frauenfeld (1864: 565, 583).

**Original spelling.***Bythiniabulimoides* var. *Aegyptiaca* Frauenfeld, 1864.

**Type locality.** “Aegypten” [Egypt].

**Remarks.** Introduced by Frauenfeld (1864: 583) as a variety of *Bithyniabulimoides* (Olivier, 1804) and on p. 565 the name is mentioned with some descriptional terms, thus, satisfying the Code (Art. 12.1).

### *Paludomusaerea* Reeve, 1852

**Original reference.**Reeve (1852: 128).

**Original spelling.***Melania æreus* Reeve, 1852.

**Type locality.** “Mountain streams of Ceylon” [Sri Lanka].

**Remarks.***Tanaliaaerens* as used by Adams and Adams (1853–1854) is an incorrect subsequent spelling.

### *Paludomusafricana* Martens, 1878

**Original reference.**Martens (1878: 297, pl. 2, figs 11–13).

**Original spelling.***PaludomusAfricana* Martens, 1878.

**Type locality.** “Finboni” [Kenya, Finboni, near the coast, between Mombassa and Taita].

### *Paludomusajanensis* Morelet, 1860

**Original reference.**Morelet (1860b: 110, pl. 6, fig. 10).

**Original spelling.***PaludomusAjanensis* Morelet, 1860.

**Type locality.** “Dans les eaux saumâtres d’Hafoun (Raz Hhafoun), à trente lieues au sud du cap Guardafui” [Somalia, brackish waters at Hafun, south of Cape Guardafui ; but probably from the Seychelles].

### *Cleopatraalhiensis* Verdcourt, 1957 [Unavailable]

**Original reference.**Verdcourt (1957: 9).

**Remarks.** Mentioned under *Cleopatraathiensis* Verdcourt, 1957 as a manuscript name attributed to H. B. Preston; unavailable (Code Art. 11.6).

### *Paramelaniaalphonsi* Bourguignat, 1890

**Original reference.**Bourguignat (1890: 217–218).

**Original spelling.***ParamelaniaAlphonsi* Bourguignat, 1890.

**Type locality.** “Sur la plage de Mlilo” [Democratic Republic of the Congo, Mlilo (= Moliro), Lake Tanganyika].

### *Melaniaamaena* Morelet, 1851

**Original reference.**Morelet (1851a: 220–221).

**Original spelling.***Melaniaamæna* Morelet, 1851.

**Type locality.** “Palustria ad orientem insulæ Madagascar” [Madagascar, swamps in the south of the island] (Morelet (1851a, b); subsequently stated to be “Zanzibar et des Séchelles” [Tanzania, Zansibar; Seychelles] (Morelet (1860b).

**Remarks.** Also published as a new species in Morelet (1851b: 192–193, pl. 5, fig. 9), which is considered here as a subsequent use.

### *Syrnolopsisanceyana* Bourguignat, 1885

**Original reference.**Bourguignat (1885a: 20).

**Type locality.** “Sur la plage de Pambété” [Zambia, beach of Pambété, Lake Tanganyika].

### *Hylacanthaanceyi* Bourguignat, 1891

**Original reference.**Bourguignat and Servain (1891: 165).

**Original spelling.***HylacanthaAnceyi* Bourguignat, 1891.

**Type locality.** Not given.

### *Syrnolopsisanceyi* Germain, 1905 [Invalid]

**Original reference.**Germain (1905a: 259).

**Original spelling.***SyrnolopsisAnceyi* Bourguignat, 1905.

**Remarks.** Unjustified emendation of *Syrnolopsisanceyana* Bourguignat, 1885. Germain (1905a) changed the suffix of several species-group names ending in -iana or -ana introduced by Bourguignat (1885, 1888). The names thus formed are to be treated as emendations of the original names (Code Art. 33.2.1) and are available and have as unjustified emendations their own author and date (Code Art. 33.2.2).

### *Paludomusandersoniana* Nevill, 1877

**Original reference.**Nevill (1877: 35).

**Original spelling.***PaludomusAndersoniana* Nevill, 1877.

**Type locality.** “Mandalay, Ava, Bhamô, Kabyuet, and Myadoung” [Myanmar, Mandalay, Ava, Bhamô, Kabuyet, Myadoung].

### † *Cleopatraangulata* Williamson, 1979

**Original reference.**Williamson (1979: 114, 227b, 238b, fig. 65t).

**Type locality.** “Area 110, east of lake Turkana, north Kenya”.

**Type stratum.** “Fauna 52, in the lower Member of the Koobi Fora Formation”.

### *Paludomusannandalei* Preston, 1909

**Original reference.**Preston (1909: 277–278).

**Type locality.** “Tenmalai, W. Ghats (W. side), Travancore (rocky mountain stream)” [India, Kerala, Thenmala, Western Ghats, western side].

### † *Cleopatraarambourgi* Roger, 1944

**Original reference.**Roger (1944: 127–128, pl. 1, figs 15–17).

**Original spelling.***CleopatraArambourgi* Roger, 1944.

**Type locality.** “De la surface du plateau, entre Kalam et Bourillé” [Ethiopia, Omo Plateau].

**Type stratum.** Not given.

**Remarks.** Attributed to L. Germain, but J. Roger alone was responsible for the description.

### *Paramelaniaarenarum* Bourguignat, 1888

**Original reference.**Bourguignat (1888: 39–40, pl. 17, figs 3, 4).

**Type locality.** “Lac Tanganika” [Lake Tanganyika].

### *Cleopatraathiensis* Verdcourt, 1957

**Original reference.**Verdcourt (1957: 8–9, fig. 2).

**Type locality.** “Kenya, Athi Plains”.

### † *Cleopatraferrugineaattenuata* Williamson, 1979

**Original reference.**Williamson (1979: 227b, 238a, pl. 2, figs 7–9).

**Type locality.** “Area 202, east of lake Turkana, north Kenya”.

**Type stratum.** “Fauna 12C, from between the upper and lower tuffs comprising the Suregei tuff complex, basal Koobi Fora Formation”.

**Remarks.** A potadomid species according to Van Bocxlaer et al. (2008).

### *Cleopatraaurocincta* Martens, 1879

**Original reference.**Martens (1879: 103)

**Type locality.** “Bagamojo, gegenüber von Zanzibar” [Tanzania, Bagamoyo].

### *Paludomusbaccula* Reeve, 1852

**Original reference.**Reeve (1852: 128).

**Type locality.** “Branch of the Ganges” [India or Bangladesh, Ganges River].

**Remarks.***Paludomusbacula* is an incorrect subsequent spelling by Layard (1855) and Adams and Adams (1853–1854).

### *Paramelaniabaizeana* Bourguignat, 1885

**Original reference.**Bourguignat (1885a: 74).

**Type locality.** “Près de Kapampa, sur la côte sud-ouest du lac” [Democratic Republic of the Congo, Kapampa, western shore of Lake Tanganyika].

### *Anceyabella* Pilsbry & Bequaert, 1927

**Original reference.**Pilsbry and Bequaert (1927: 238, figs 36, 37).

**Type locality.** “Lake Tanganyika”.

### *Pseudocleopatrabennikei* Mandahl-Barth, 1974

**Original reference.** Mandahl-Barth (1974: 355–356, fig. 2a).

**Type locality.** “River Zaire between Mimosa Island and Monkey Island, off Kinshasa” [Democratic Republic of the Congo].

### *Stomatodonbensoni* Brot, 1880

**Original reference.**Brot (1880: 13–14, pl. 5, fig. 2, 2a, 2b).

**Original spelling.***StomatodonBensoni* Brot, 1880.

**Type locality.** “Südliches Indien, Travancore (Benson), Malabar (Coll. Mea)” [India, Kerala, Travancore; Malabar].

### *Cleopatrabequaerti* Dautzenberg & Germain, 1914

**Original reference.**Dautzenberg and Germain (1914: 59–60, pl. 4, figs 1–6).

**Original spelling.***CleopatraBequaerti* Dautzenberg & Germain, 1914.

**Type locality.** “Kindu, Lualaba, 3° lat. S” [Democratic Republic of the Congo, Kindu, Lualaba River].

### *Cleopatrabulimoidesbicarinata* Pallary, 1924

**Original reference.**Pallary (1924: 33).

**Original spelling.**Cleopatrabulimoidesvar.bicarinata Pallary, 1924.

**Type locality.** “Égypte” [Egypt].

### *Paludomusbicincta* Reeve, 1852

**Original reference.**Reeve (1852: 129).

**Original spelling.***Paludomusbicinctus* Bourguignat, 1852.

**Type locality.** “Mountain streams of Ceylon” [Sri Lanka].

### *Paludomusbifasciatus* Adams & Adams, 1854 [Unavailable]

**Original reference.**Adams and Adams (1853–1854: 340).

**Remarks.** Attributed to Reeve. Nomen nudum, introduced without description, definition, or indication (Code Art. 12.1).

### *Paludomusbilineata* Troschel, 1857

**Original reference.** Troschel (1856–1863: 101, pl. 7, fig. 7).

**Type locality.** Not given.

**Original spelling.***Paludomusbilineatus* Troschel, 1857.

**Remarks.** Troschel (1857) in Troschel (1856–1863: 101, pl. 7, fig. 7) described and figured a radula under the name *Paludomusbilineatus*, attributing the name to L. A. Reeve. However, Reeve (1847, 1852) did not introduce the name *Paludomusbilineatus*, but instead *Paludomusbicinctus* Reeve, 1852 with a similar meaning. Although the name *P.bilineatus* is probably the result of a lapsus memoriae, it is deemed available here.

### *Cleopatrabulimoidesbilirata* Germain, 1918

**Original reference.**Germain (1918: 444–445).

**Original spelling.***Cleopatrabulimoides* variété *bilirata* Germain, 1918.

**Type locality.** “Rosières [= Rosaires = Abramat], sur le Nil Bleu” [Sudan, Er Roseires, Blue Nile].

### *Paludomusbinaluanensis* Thiele, 1928

**Original reference.** Thiele (1828: 392, pl. 8, fig. 17).

**Type locality.** “Von Binaluan auf Nord-Palawan” [Philippines, north Palawan, Binaluan].

### *Paludomusblanfordiana* Nevill ,1877

**Original reference.**Nevill (1877: 37).

**Original spelling.***PaludomusBlanfordiana* Nevill, 1877.

**Type locality.** “Ava” [Myanmar, Ava].

### *Paludomusborneensis* Nevill, 1884

**Original reference.**Nevill (1884: 292).

**Type locality.** “Sadong, Sarawak” [Malaysia, Borneo, Sarawak, Sadong Jaya].

### *Paramelaniabourguignati* Bourguignat, 1885

**Original reference.**Bourguignat (1885a: 73–74).

**Type locality.** “Plage de Kapampa” [Democratic Republic of the Congo, beach of Kapampa, western shore of Lake Tanganyika].

**Remarks.** Attributed to V. Giraud by Bourguignat (1885a: 73), but Bourguignat alone was responsible for the description. Therefore, Bourguignat is the author of the name (Code Art. 50.1).

### *Tiphobiabourguignati* Bourguignat, 1886

**Original reference.**Bourguignat (1886: 148–150, pl. 6, figs 5–7).

**Type locality.** “Dans l’anse de Kamangu, un peu S.-O. des îlots Kilira Chakabala“ [Democratic Republic of the Congo, Kamangu Bay, southwest of the Kilira Chakabala Islands]].

**Remarks.** Attributed to L. Joubert by Bourguignat (1886: 148) but Bourguignat alone was responsible for the description. Therefore, Bourguignat is the author of the name (Code Art. 50.1).

### *Bourguignatiabridouxi* Bourguignat, 1888

**Original reference.**Bourguignat (1888: 29, pl. 12, figs 1–4).

**Original spelling.***BourguignatiaBridouxi* Bourguignat, 1888.

**Type locality.** “Sur les plages de la côte occidentale” [Democratic Republic of the Congo, western shores of Lake Tanganyika].

### *Viviparabridouxiana* Bourguignat, 1888

**Original reference.**Bourguignat (1888: 13–14, pl. 4, fig. 2).

**Original spelling.***ViviparaBridouxiana* Bourguignat, 1888.

**Type locality.** “De la côte nord-occidentale du lac” [Democratic Republic of the Congo, north-western shore of Lake Tanganyika].

### *Hauttecoeuriabridouxiana* Bourguignat, 1888

**Original reference.**Bourguignat (1888: 21–22, pl. 8, figs 26–28).

**Original spelling.***Hauttecœuria Bridouxiana* Bourguignat, 1888.

**Type locality.** “Lac Tanganika” [Lake Tanganyika].

### *Reymondiabridouxiana* Bourguignat, 1888

**Original reference.**Bourguignat (1888: 27–28, pl. 11, figs 14, 15).

**Original spelling.***ReymondiaBridouxiana* Bourguignat, 1888.

**Type locality.** “Lac Tanganika” [Lake Tanganyika].

### *Viviparabrincatiana* Bourguignat, 1888

**Original reference.**Bourguignat (1888: 13–14, pl. 4, fig. 1).

**Original spelling.***ViviparaBrincatiana* Bourguignat, 1888.

**Type locality.** “De la côte nord-occidentale du lac” [Democratic Republic of the Congo, north-western shore of Lake Tanganyika].

### *Hauttecoeuriabrincatiana* Bourguignat, 1888

**Original reference.**Bourguignat (1888: 19, pl. 7, figs 20, 21).

**Original spelling.***Hauttecœuria Brincatiana* Bourguignat, 1888.

**Type locality.** “Lac Tanganika” [Lake Tanganyika].

### *Cleopatrabroecki* Putzeys, 1899

**Original reference.**Putzeys (1899: 60, fig. 16).

**Original spelling.***CleopatraBroecki* Putzeys, 1899.

**Type locality.** “De la rivière Aruwimi, affluent du Congo” [Democratic Republic of the Cong, Aruwimi River, tributary of Congo River].

### *Paludomusbroti* Issel, 1874

**Original reference.**Issel (1874: 455–456, pl. 7, figs 19, 20).

**Original spelling.***PaludomusBroti* Issel, 1874.

**Type locality.** “Sadong, Sarawak” [Malaysia, Borneo, Sarawak, Sadong Jaya].

### *Cyclostomabulimoides* Olivier, 1804

**Original reference.**Olivier (1804: 39, pl. 31, fig. 6).

**Type locality.** “Le Kalidje” [Egypt, Le Kalidje near Alexandria].

### *Paludomusburmanica* Nevill, 1877

**Original reference.**Nevill (1877: 36).

**Original spelling.***PaludomusBurmanica* Nevill, 1877.

**Type locality.** “Yaylaymaw and Mandalay” [Myanmar, Yaylaymaw and Mandalay].

### *Hauttecoeuriaburtoni* Bourguignat, 1888

**Original reference.**Bourguignat (1888: 19, pl. 7, figs 1–3).

**Original spelling.***Hauttecœuria Burtoni* Bourguignat, 1888.

**Type locality.** “Lac Tanganika” [Lake Tanganyika].

### *Paramelaniabythiniformis* Bourguignat, 1888

**Original reference.**Bourguignat (1888: 35–36, pl. 15, figs 26, 27).

**Original spelling.***ParamelaniaBythiniformis* Bourguignat, 1888.

**Type locality.** “Lac Tanganika” [Lake Tanganyika].

### *Rumellacallifera* Bourguignat, 1888

**Original reference.**Bourguignat (1888: 39–40, pl. 17, figs 23–25).

**Type locality.** “De la côte occidentale” [Democratic Republic of the Congo, Lake Tanganyika, western shore].

### *Lavigeriacallista* Bourguignat, 1888

**Original reference.**Bourguignat (1888: 33–34, pl. 14, fig. 2).

**Type locality.** “Sur les rives nord et surtout occidentales du lac” [Democratic Republic of the Congo, northern and western shores of Lake Tanganyika].

### *Paramelaniacallopleuros* Bourguignat, 1885

**Original reference.**Bourguignat (1885a: 69–70).

**Type locality.** “Plage de Pambété” [Zambia, beach of Pambété, Lake Tanganyika].

### *Hauttecoeuriacambieri* Bourguignat, 1885

**Original reference.**Bourguignat (1885a: 56–57).

**Original spelling.***Hauttecœuria cambieri* Bourguignat, 1885.

**Type locality.** “Plage de Mpala” [Democratic Republic of the Congo, beach of Mpala].

**Remarks.** The name was attributed to V. Giraud by Bourguignat (1885a: 56) but Bourguignat alone was responsible for the description. Therefore, Bourguignat is the author of the name (Code Art. 50.1).

### *Cleopatracameroni* Bourguignat, 1879

**Original reference.**Bourguignat (1879: 21–22).

**Original spelling.***CleopatraCameroni* Bourguignat, 1879.

**Type locality.** “Fleuve Kyngani, près de Bagamoyo” [Tanzania, Bagamoyo, Kyngani River].

### *Spekiacameroni* Bourguignat, 1888

**Original reference.**Bourguignat (1888: 15, pl. 5, figs 13–15).

**Original spelling.***SpekiaCameroni* Bourguignat, 1888.

**Type locality.** “Lac Tanganika” [Lake Tanganyika].

### *Hauttecœuriacameroni* Bourguignat, 1888

**Original reference.**Bourguignat (1888: 21–22, pl. 8, figs 15–17).

**Original spelling.***HauttecœuriaCameroni* Bourguignat, 1888.

**Type locality.** “Lac Tanganika” [Lake Tanganyika].

### *Paramelaniacameroniana* Bourguignat, 1885

**Original reference.**Bourguignat (1885a: 80).

**Type locality.** “Sur la plage de Mlilo et sur celle de Kapampa, à l’ouest du lac” [Democratic Republic of the Congo, beach of Mlilo (= Moliro) and beach of Kapampa, Lake Tanganyika].

### *Edgarianassacamerounensis* Leloup, 1953 [Unavailable]

**Original reference.**Leloup (1953: 166).

**Original spelling.**Edgarianassaf.camerounensis Leloup, 1953.

**Remarks.** Mentioned in the synonymy of *Edgardianassa* forme *typica*; unavailable (Code Art. 11.6)

### *Cleopatracara* Pilsbry & Bequaert, 1927

**Original reference.**Pilsbry and Bequaert (1927: 294, fig. 53).

**Type locality.** “Stanleyville” [Democratic Republic of the Congo, Kisangani].

### † *Pseudocleopatracarinata* Van Damme & Pickford, 2003

**Original reference.**Van Damme and Pickford (2003: 10, 56–58, 63, 66, fig. 44).

**Type locality.** “Nkondo locality NK 120, Lake Albert, Uganda”.

**Type stratum.** “Nkondo Formation, Molluscan Association G3b, Terminal Miocene-Early Pliocene”.

### *Syrnolopsiscarinifera* Smith, 1889

**Original reference.**Smith (1889: 174).

**Type locality.** “Lake Tanganyika”.

### *Cleopatracarinulata* Dautzenberg, 1894

**Original reference.**Dautzenberg (1894: 105–106).

**Type locality.** “Ambohimarina, province Hova d’Antankara près Diego-Suarez” [Madagascar, Diana, Ambohimarina].

### *Randabeliacatoxia* Bourguignat, 1888

**Original reference.**Bourguignat (1888: 31, pl. 13, figs 1, 2).

**Type locality.** “Côtes occidentales et méridionales du lac” [Democratic Republic of the Congo/Zambia, western and southern shores of Lake Tanganyika].

### *Hauttecoeuriacharmetanti* Bourguignat, 1888

**Original reference.**Bourguignat (1888: 19, pl. 7, figs 15–17).

**Original spelling.***Hauttecœuria Charmetanti* Bourguignat, 1888.

**Type locality.** “Lac Tanganika” [Lake Tanganyika].

### *Paludomusconicacherraensis* Nevill, 1884

**Original reference.**Nevill (1884: 288).

**Original spelling.**Paludomusconicasubvar.cherraensis Nevill, 1884.

**Type locality.** “Teria Ghat” [India, Megalhaya, Khasi Hills, Teria Ghat].

**Remarks.**Nevill (1884) distinguished between ‘subspecies’, ‘var.’, and ‘subvar.’ Not every subvariety was referred to a subspecies or variety, however, but for example on p. 288 subvarieties were directly associated with a species without intermittent subspecies or variety. It seems that Nevill (1884) expressed by using the different terms to what extent a named taxon differed from the nominal species, with subspecies differing considerably, varieties slightly, and subvarieties only very slightly. Therefore, subspecific and varietal names are regarded here as subspecific names in accordance with the Code (Art. 45.6.4). The names denoted by ‘subvar.’ are, however, regarded as infrasubspecific. Made available by Kobelt in Pfeffer and Kobelt (1887: 422) (Code Art. 45.6.4.1).

### *Paludomuschilinoides* Reeve, 1847

**Original reference.**Reeve (1847: pl. 2, fig. 7a, pl. 3, fig. 7b, c).

**Original spelling.***PaludomusChilinoides* Reeve, 1847.

**Type locality.** “Bed of the Mahawelle Ganga, near Kandy, Ceylon” [Sri Lanka, Kandy, Mahawelle River].

### *Paludomusconicachittagongensis* Nevill, 1884

**Original reference.**Nevill (1884: 288).

**Original spelling.**Paludomusconicasubvar.chittagongensis Nevill, 1884.

**Type locality.** “Chittagong” [Bangladesh, Chittagong].

**Remarks.** See Remarks under *Paludomusconicacherraensis* Nevill, 1884. Made available by Kobelt in Pfeffer and Kobelt (1887: 422) (Code Art. 45.6.4.1).

### *Paludomuscincta* Liu, Zhang & Duan, 1994

**Original reference.**Liu et al. (1994: 30, 35).

**Original spelling.***Paludomuscinctus* Liu, Zhang & Duan, 1994.

**Type locality.** “Sinan (27.9°N, 108.2°E), Guizhou Province. On stones or rocks at the bottom of Wujiang River”.

### *Paludomuscingulata* Martens, 1878

**Original reference.**Martens (1878: pl. 2, figs 14–16).

**Type locality.** “Finboni” [Kenya, Finboni, near the coast, between Mombassa and Taita].

**Remarks.** The names *Paludomusexarata* and *Paludomuscingulata* were established simultaneously by Martens (1878). On pl. 2 Martens (1878) used the name *Paludomuscingulata*, but in the text and explanation of the plates the name *Paludomusexarata* was used. Bourguignat (1885b: 29) used the name in the combination *Cleopatraexarata* as the valid name of the taxon and remarked in a footnote “Ce nom a été modifié en celui de *Cingulata* sur l’explication de la planche”. Acting as first reviser, Bourguignat (1885) thus established the precedence of *Paludomusexarata* Martens, 1878 over *Paludomuscingulata* Martens, 1878.

### † *Edgariacingulata* Cox, 1939

**Original reference.**Cox (1939: 247–248, pl. 15, fig. 8).

**Type locality.** ”The Rungwa Sleeping Sickness Concentration Area, lies about eighty miles to the north-west of the present shore of the lake” [Tanzania, Rungwa].

**Type stratum.** “Quaternary deposits of Lake Rukwa”.

### *Paludomusclavata* Reeve, 1852

**Original reference.**Reeve (1852: 129).

**Original spelling.***Paludomusclavatus* Reeve, 1852.

**Type locality.** “Mountain streams of Ceylon” [Sri Lanka].

### *Paludinacleopatra* Martens, 1860 [Unavailable]

**Original reference.** Martens (1860: 13).

**Original spelling.***Paludinabulimoides* Olivier, 1804 (*cleopatra* Troschel)

**Remarks.** Published as a synonym of *Paludinabulimoides* (Olivier, 1804) and attributed to Troschel who introduced *Cleopatra* as a genus-group name in 1857. Treated by Frauenfeld (1864: 588) as an available name, but not in a way that satisfies it to be adopted (Code, Glossary), i.e. not satisfying the Code (Art. 12.1). Therefore, the name was not made available under the Code (Art. 11.6.1) by Frauenfeld (1864).

### *Spekiacoheni* Irvine & Donohue, 1999 [Unavailable]

**Original reference.**Irvine and Donohue (1999: 11).

**Remarks.** Introduced without description or definition (Code Art. 13.1.1). Mentioned also by Alin et al. (1999) as Spekia n. sp. “*coheni*” and by Allison et al. (2000) and Ngereza (2010) as *Spekiacoheni*.

### *Paludinacolbeaui* Craven, 1880

**Original reference.**Craven (1880: 216, pl. 22, fig. 5).

**Type locality.** “Nossi-Bé Island, in a sluggish stream running through coffee-plantations” [Madagascar, Antsiranana, Nosy Be Island].

### † *Heynderycxiacolumellaris* Van Damme & Pickford, 2003

**Original reference.**Van Damme and Pickford (2003: 10, 60–61, 67, 77, figs 49a–d, 54c).

**Type locality.** “Nkondo locality NK 77, Lake Albert, Uganda”.

**Type stratum.** “Nkondo Formation, Molluscan Association G3b, Terminal Miocene to Early Pliocene”.

### *Lavigeriacombsa* Bourguignat, 1888

**Original reference.**Bourguignat (1888: 33–34, pl. 14, fig. 7).

**Type locality.** “Sur les rives nord et surtout occidentales du lac” [Democratic Republic of the Congo, northern and western shores of Lake Tanganyika].

### *Paludomussulcatacompacta* Nevill, 1884

**Original reference.**Nevill (1884: 299).

**Original spelling.***Paludomus* [*Philopotamis*] *sulcata*var.compacta Nevill, 1884.

**Type locality.** “Ceylon” [Sri Lanka].

**Remarks.** See Remarks under *Paludomusconicacherraensis* Nevill, 1884.

### *Cleopatracongener* Preston, 1913

**Original reference.**Preston (1913: 59, pl. 4, fig. 6).

**Type locality.** “Lake Baringo, British East Africa” [Kenya, Lake Baringo].

### *Melaniaconica* Griffith & Pidgeon, 1833 [Invalid]

**Original reference.**Griffith and Pidgeon (1833–1834: pl. 14, fig. 5).

**Type locality.** “Ceylon” [Sri Lanka] (Griffith and Pidgeon 1833–1834: 598).

**Remarks.** Primary junior homonym of *Melaniaconica* Say, 1821. Although authorship is usually attributed to Gray (see Petit and Coan 2008), the requirements of the Code (Art. 50.1) are not fulfilled in our opinion. The authorship should therefore be attributed to Griffith and Pidgeon.

### *Paludomusconstricta* Reeve, 1852

**Original reference.**Reeve (1852: 129).

**Original spelling.***Paludomusconstrictus* Reeve, 1852.

**Type locality.** “Mountain streams of Ceylon” [Sri Lanka].

### *Paludomussulcatacontracta* Nevill, 1884

**Original reference.**Nevill (1884: 299).

**Original spelling.***Paludomus* [*Philopotamis*] *sulcata*var.contracta Nevill, 1884.

**Type locality.** “Peradenia [and] Ambegammoa, Ceylon” [Sri Lanka].

**Remarks.** See Remarks under *Paludomusconicacherraensis* Nevill, 1884.

### *Cleopatraconvexa* Verdcourt, 1957

**Original reference.**Verdcourt (1957: 7–8, fig. 1).

**Type locality.** “Kenya, Turkana Province, Clay pan 7 miles S. E. Lodwar (subfossil)”.

### *Melaniacoronata* von dem Busch, 1842

**Original reference.**Philippi (1842–1845: 2, pl. 1, figs 5, 6).

**Type locality.** “Bengalia” [India/Bangladesh].

**Remarks.** The entire text with descriptions of von dem Busch’s new *Melania* names was attributed to von dem Busch on p. 4 of Philippi’s (1842–1845) work.

### *Lavigeriacoronata* Bourguignat, 1888

**Original reference.**Bourguignat (1888: 31, pl. 13, figs 13, 14).

**Type locality.** “Sur les rives nord et surtout occidentales du lac” [Democratic Republic of the Congo, northern and western shores of Lake Tanganyika].

### *Bridouxiacostata* Bourguignat, 1885

**Original reference.**Bourguignat (1885a: 31–32).

**Type locality.** “Environs de Kapampa” [Democratic Republic of the Congo, surroundings of Kapampa, western shore of Lake Tanganyika].

### *Cleopatramorellicostata* Preston, 1905

**Original reference.**Preston (1905: 300–301, fig. 4).

**Original spelling.**CleopatraMorellivar.costata Preston, 1905.

**Type locality.** “Victoria Falls, Zambesi River” [Zimbabwe, Zambesi River, Victoria Falls].

### *Melaniacrassa* von dem Busch, 1842

**Original reference.**Philippi (1842–1845: 2–3, pl. 1, figs 10, 11).

**Type locality.** “Bengalia” [India/Bangladesh].

**Remarks.** The entire text with descriptions of von dem Busch’s new *Melania* names was attributed to von dem Busch on p. 4 of Philippi’s (1842–1845) work.

### † *Taeniodomuscrassa* Krause, 1897

**Original reference.**Krause (1897: 214, 219, pl. 12, figs 2, 2a, 3).

**Type locality.** “Thonmergel vom Sungei Pinoh” [Indonesia, Borneo, Sungai Pinoh].

**Type stratum.** “α Eocaen (der Sandstein Etage) Verbeek‘s” [Eocene, Verbeek’s sandstone stage].

### *Paludomuscrassicallosa* Rao, 1929

**Original reference.**Rao (1929: 288–290).

**Type locality.** “Sankha, a large hill-stream between Kamaing and Mogaung” [Myanmar].

### *Tiphobiacrassigranulata* Smith, 1881

**Original reference.**Smith (1881b: 560).

**Original spelling.**Tiphobia (Paramelania) crassigranulata Smith, 1881.

**Type locality.** “Lake Tanganyika”.

**Remarks.***Paramelaniacranigranulata* as used by Moore (1903) is an incorrect subsequent spelling.

### *Paramelaniacrassilabris* Bourguignat, 1885

**Original reference.**Bourguignat (1885a: 84–85).

**Type locality.** “Plage Mlilo” [Democratic Republic of the Congo, Mlilo (= Moliro), Lake Tanganyika].

### *Cleopatracridlandi* Mandahl-Barth, 1954

**Original reference.**Mandahl-Barth (1954: 61–62, fig. 27a, b).

**Original spelling.***Cleopatracridlandicridlandi* Mandahl-Barth, 1954.

**Type locality.** “Lake Victoria, where it was dredged off Dagusi Island from a depth of 20 to 40 feet” [Uganda].

### *Paludomuscumingiana* Dohrn, 1857

**Original reference.**Dohrn (1857: 124).

**Original spelling.***Paludomus Cumingianus* Dohrn, 1857.

**Type locality.** “Ceylon” [Sri Lanka].

**Remarks.** The name *Paludomus Cumingii* as used by Brot (1862: 21) and Brot (1868: 2) is an incorrect subsequent spelling.

### *Limnotrochuscyclostoma* Bourguignat, 1885

**Original reference.**Bourguignat (1885a: 60–61).

**Type locality.** “Plage de Pambété” [Zambia, beach of Pambété, Lake Tanganyika].

### *Paludinacyclostomoides* Küster, 1852

**Original reference.**Küster (1852: 32, pl. 7, figs 6–10).

**Type locality.** “In Aegypten” [Egypt].

### *Cleopatracridlandicylindrica* Mandahl-Barth, 1954

**Original reference.**Mandahl-Barth (1954: 62, fig. 27c–d).

**Type locality.** “From a depth of 60 feet in Buvuma Channel” [Uganda, Buvuma District, Buvuma Channel].

### † *Viviparuscylindricus* Adam, 1957

**Original reference.**Adam (1957: 35–37, pl. 4, figs 1–5).

**Type locality.** “Rive du ravin de Nyakasia, 65 à 80 m sur lac” [Uganda, river of Nyakasia Ravine, 65 to 80 m above Lake Albert].

**Type stratum.** “Pleistocène inférieur, Série de Kaiso” [Lower Pleistocene, Kaiso Series].

**Type material.** Holotype, Musée royal de l’Afrique centrale, Tervuren MC 2780 (Adam 1957, pl. 4, fig. 1).

**Remarks.** The species was classified as *Cleopatracylindrica* (Adam, 1957) by Van Damme and Pickford (2003) in their revision of Albertine Rift Valley freshwater Cerithioidea. By that taxonomic act, the taxon becomes a secondary junior homonym of the nominal taxon *Cleopatracridlandicylindrica* Mandahl-Barth, 1954 and as no junior synonyms exist, the new replacement name *Cleopatraadami* nom. nov. is proposed here (see also Remarks under that name above).

### *Tiphobiadamoni* Smith, 1881

**Original reference.**Smith (1881b: 559–560).

**Original spelling.**Tiphobia (Paramelania) damoni Smith, 1881.

**Type locality.** “Lake Tanganyika”.

### *Pseudocleopatradartevellei* Mandahl-Barth, 1973

**Original reference.**Mandahl-Barth (1973: 280–281, fig. 3d–f, i).

**Type locality.** “River Congo at Matadi” [Democratic Republic of the Congo, Matadi, Congo River].

### *Giraudiahoreidautzenbergi* Germain, 1905

**Original reference.**Germain (1905a: 259).

**Original spelling.***GiraudiaHorei* var. *Dautzenbergi* Germain, 1905.

**Type locality.** “Lac Tanganika” [Lake Tanganyika].

### *Marteliadautzenbergi* Dupuis, 1924

**Original reference.**Dupuis (1924: 20–21, 26, fig. 1).

**Original spelling.***MarteliaDautzenbergi* Dupuis, 1924.

**Type locality.** “Tanganika”…”de la côte occidentale du Lac” [Democratic Republic of the Congo, Lake Tanganyika, western shore].

### *Cleopatradautzenbergi* Pilsbry & Bequaert, 1927

**Original reference.**Pilsbry and Bequaert (1927: 294–295, fig. 54).

**Type locality.** “Lovoi River, near Kikondja” [Democratic Republic of the Congo, near Kikondja, Lovoi River].

### *Paludomusdecussata* Reeve, 1852

**Original reference.**Reeve (1852: 127–128).

**Original spelling.***Paludomusdecussatus* Reeve, 1852.

**Type locality.** “Ceylon” [Sri Lanka].

### *Melaniadenticulata* Benson, 1836

**Original reference.**Benson (1836b: 748).

**Type locality.** “From the north-east frontier of Bengal (Benson 1836a: 350)” [India/Bangladesh].

**Remarks.** The name was conditionally proposed.

### *Paludomusdhuma* Rao, 1925

**Original reference.**Rao (1925: 97).

**Type locality.** “A stream near Thazi Railway Station” [Myanmar].

### *Lavigeriadiademata* Bourguignat, 1888

**Original reference.**Bourguignat (1888: 31, 34, pl. 13, figs 15–17).

**Type locality.** “Sur les rives nord et surtout occidentales du lac” [Democratic Republic of the Congo, northern and western shores of Lake Tanganyika].

### *Paludomusconicadihiriensis* Nevill, 1884

**Original reference.**Nevill (1884: 288).

**Original spelling.**Paludomusconicasubvar.dihiriensis Nevill, 1884.

**Type locality.** “Stream on the Dihiri Hill [Brahmaputra watershed]” [India, Assam].

**Remarks.** See Remarks under *Paludomusconicacherraensis* Nevill, 1884. Made available by Kobelt in Pfeffer and Kobelt (1887: 422) (Code Art. 45.6.4.1).

### *Paludomusdilatata* Reeve, 1852

**Original reference.**Reeve (1852: 128–129).

**Original spelling.***Paludomusdilatatus* Reeve, 1852.

**Type locality.** “Mountain streams of Ceylon” [Sri Lanka].

### *Paludomusdistinguenda* Dohrn, 1857

**Original reference.**Dohrn (1857: 124).

**Original spelling.***Paludomusdistinguendus* Dohrn, 1857.

**Type locality.** “Ceylon” [Sri Lanka].

### *Paludomusdromedarius* Dohrn, 1857

**Original reference.**Dohrn (1857: 124).

**Type locality.** Ceylon [Sri Lanka].

### † *Cleopatradubia* Adam, 1959

**Original reference.**Adam and Lepersonne (1959: 34–38, pl. 5, figs 1–3, pl. 6, figs 1, 2).

**Type locality.** “Nyamavi no 68, camp Nyamavi” through selection of lectotype [Democratic Republic of the Congo, Nyamavi, near Lake Albert].

**Remarks.** The statement by Van Damme and Pickford (2003: 22) that the name *Cleopatradubia* Adam, 1959 is a nomen nudum is incorrect because W. Adam in Adam and Lepersonne (1959: 34–38) correctly introduced it, although not explicitly selecting a holotype and a type locality. Furthermore, the type selection of Van Damme and Pickford (2003: 49–50) does not constitute a valid lectotype designation in accordance with the Code Art. 74.7.1 because the term lectotype or an exact translation of that term was used not by these authors but only the term type and holotype. To avoid further confusion and nomenclatural instability, we here select the specimen proposed as type/holotype by Van Damme and Pickford (2003), i.e. the specimen figured by Adam (1959: pl. 5, fig. 1a), leg. J. Lepersonne, Lep. 464 III (M.C. 2382) from Nyamavi no 68, camp Nyamavi, as lectotype of *Cleopatradubia* Adam, 1959.

### *Hauttecoeuriaduveyieri* Germain, 1905 [Invalid]

**Original reference.**Germain (1905a: 258).

**Original spelling.***Hauttecœuria Duveyieri* Germain, 1905.

**Remarks.** Unjustified emendation of *Hauttecœuriaduveyrieriana* Bourguignat, 1885. Germain (1905a) changed the suffix of several species-group names ending in -iana or -ana introduced by Bourguignat (1885, 1888). The names thus formed are to be treated as emendations of the original names (Code Art. 33.2.1) and are available and have as unjustified emendations their own author and date (Code Art. 33.2.2).

### *Spekiaduveyieri* Germain, 1906 [Invalid]

**Original reference.**Germain (1906: 578).

**Original spelling.***SpekiaDuveyieri* Germain, 1906.

**Remarks.** Unjustified emendation of *Spekiaduveyrieriana* Bourguignat, 1885. Germain (1906) changed the suffix of several species-group names ending in -iana or -ana introduced by Bourguignat (1885). The names thus formed are to be treated as emendations of the original names (Code Art. 33.2.1) and are available and have as unjustified emendations their own author and date (Code Art. 33.2.2).

### *Spekiaduveyrieriana* Bourguignat, 1885

**Original reference.**Bourguignat (1885a: 37–38).

**Type locality.** “Mpala, dans le Marungu, sur la côte sud-ouest du lac” [Democratic Republic of the Congo, Mpala, Marungu region, at the south-western coast of Lake Tanganika].

### *Hauttecœuriaduveyrieriana* Bourguignat, 1885

**Original reference.**Bourguignat (1885a: 53).

**Type locality.** “Plage de Pambété” [Zambia, beach of Pambété, Lake Tanganyika].

### *Paramelaniaduveyrieriana* Bourguignat, 1885

**Original reference.**Bourguignat (1885a: 79).

**Type locality.** “Plage de Mlilo” [Democratic Republic of the Congo, Mlilo (= Moliro), Lake Tanganyika].

**Remarks.** The name was attributed to V. Giraud by Bourguignat (1885a: 79) but Bourguignat alone was responsible for the description and is therefore the author of the name alone (Code 50.1).

### *Anceyagiraudiecarinata* Leloup, 1953 [Unavailable]

**Original spelling.**Anceyagiraudivar.ecarinata Leloup, 1953.

**Original reference.**Leloup (1953: 108, 111–112, fig. 55B).

**Remarks.** Mentioned in the synonymy of Anceya (Anceya) giraudi as a manuscript name of Dupuis; unavailable (Code Art. 11.6).

### *Paramelaniaegregia* Bourguignat, 1885

**Original reference.**Bourguignat (1885a: 81).

**Type locality.** “À Kapampa” [Democratic Republic of the Congo, Kapampa, western shore of Lake Tanganyika].

**Remarks.** The name was attributed to V. Giraud by Bourguignat (1885a: 81) but Bourguignat alone was responsible for the description and therefore is the author of the name (Code Art. 50.1).

### *Spekiazonataelata* Germain, 1906

**Original reference.**Germain (1906: 579).

**Original spelling.**Spekiazonatavar.elata Germain, 1906.

**Type locality.** “À Mpala, sur la côte ouest, ainsi qu’à Pambété, au sud du lac” as given in Bourguignat (1885a) [Democratic Republic of the Congo, Mpala, western shore of Lake Tanganyika and Zambia, Pambété, southern shore of Lake Tanganyika].

### *Cleopatrapirothielata* Dautzenberg & Germain, 1914

**Original reference.**Dautzenberg and Germain (1914: 57–58).

**Original spelling.**CleopatraPirothif.elata Dautzenberg & Germain, 1914.

**Type locality.** “Stn. 92, Bulongo (Bukama), 9° lat. S” [Democratic Republic of the Congo, Bulongo (Bukama), near Lualaba River].

### *Spekiazonataelongata* Bourguignat, 1888

**Original reference.**Bourguignat (1888: 14, pl. 4, fig. 23).

**Original spelling.**Spekiazonatavar.elongata Bourguignat, 1888.

**Type locality.** “Lac Tanganika” [Lake Tanganyika].

### *Paramelaniaelongata* Bourguignat, 1888

**Original reference.**Bourguignat (1888: 37–38, pl. 16, figs 9, 10).

**Type locality.** “Lac Tanganika” [Lake Tanganyika].

### *Paludomusemini* Smith, 1888

**Original reference.**Smith (1888: 54).

**Type locality.** “Albert Nyanza” [Democratic Republic of the Congo/Uganda, Lake Albert].

### *Paludomuserinaceus* Reeve, 1852

**Original reference.**Reeve (1852: 128).

**Type locality.** “Mountain streams of Ceylon” [Sri Lanka].

**Remarks.***Tanaliacrinascens* is an incorrect subsequent spelling by Layard (1855) and *Tanaliacrinacea* is an incorrect subsequent spelling by Adams and Adams (1853–1854).

### *Paludomuserronea* Nevill, 1884

**Original reference.**Nevill (1884: 300).

**Original spelling.***Paludomus* [*Philopotamis*] *erronea* Nevill, 1884.

**Type locality.** “Ceylon [and] Hackalle, Ceylon [at 5,500 ft]” [Sri Lanka].

### *Paludomuseveretti* Smith, 1894

**Original reference.**Smith (1894: 51).

**Type locality.** “Batang Lupar district, W. Sarawak, also Gomanton on the N.E. Coast at Sandakan Bay” [Malaysia/Indonesia, Borneo].

### *Paludomusexarata* Martens, 1878

**Original reference.**Martens (1878: 297–298, pl. 2, figs 14–16).

**Type locality.** “Finboni” [Kenya, Finboni, near the coast, between Mombassa and Taita].

### *Hauttecoeuriaeximia* Bourguignat, 1885

**Original reference.**Bourguignat (1885a: 55–56).

**Original spelling.***Hauttecœuria eximia* Bourguignat, 1885.

**Type locality.** “Plage de Mpala, à l’ouest du lac” [Democratic Republic of the Congo, Mpala, western shore of Lake Tanganyika].

### *Tanganikiafagoti* Germain, 1908

**Original reference.**Germain (1908: 36).

**Original spelling.***TanganikiaFagoti* Germain, 1908.

**Remarks.** Unjustified emendation of *Tanganikiafagotiana* Bourguignat, 1885.

### *Tanganikiafagotiana* Bourguignat, 1885

**Original reference.**Bourguignat (1885a: 43).

**Original spelling.**Tanganikia (Tanganikia) fagotiana Bourguignat, 1885.

**Type locality.** “Dans la partie septentrionale du lac” and “également dans sa partie méridionale […] sur la plage de Pambété” [Democratic Republic of the Congo or Burundi, northern part of Lake Tanganyika; Zambia, Pambété, southern shore of Lake Tanganyika].

### *Paludinafasciata* Kobelt, 1909 [Unavailable]

**Original reference.**Kobelt (1909: 386).

**Type locality.** Not given.

**Remarks.** Published in the synonymy of *Cleopatrabulimoides* (Olivier, 1804); unavailable (Code Art. 11.6).

### *Melaniaferruginea* Lea & Lea, 1851

**Original reference.**Lea and Lea (1851: 182).

**Type locality.** “Zanzibar, East Africa” [Tanzania, Zanzibar].

### *Paramelaniaflexicosta* Martens, 1895

**Original reference.**Martens (1895: 186–187).

**Original spelling.**Paramelania (Edgaria) flexicosta Martens, 1895.

**Type locality.** “Tanganyika” [Lake Tanganyika].

### *Helixfluviatilis* Dillwyn, 1817

**Original reference.**Dillwyn (1817: 959).

**Type locality.** “Inhabits fresh water in Coromandel” [India, Coromandel].

### *Syrnolopsisfoai* Mabille, 1901

**Original reference.**Mabille (1901: 56).

**Type locality.** “Lac Tanganika” [Lake Tanganyika].

### *Assimineafoai* Mabille, 1901

**Original reference.**Mabille (1901: 56–57).

**Type locality.** “Lac Tanganika” [Lake Tanganyika].

**Remarks.***Assimineafoas* is an incorrect subsequent spelling by Pilsbry and Bequaert (1927).

### *Reymondiafoai* Mabille, 1901

**Original reference.**Mabille (1901: 57).

**Original spelling.***ReymondiaFoai* Mabille, 1901.

**Type locality.** “Lac Tanganika” [Lake Tanganyika].

### *Paramelaniaformosa* Bourguignat, 1888

**Original reference.**Bourguignat (1888: 35–36, pl. 15, figs 9–11).

**Type locality.** “Lac Tanganika” [Lake Tanganyika].

### *Paludomusfulgurata* Dohrn, 1857

**Original reference.**Dohrn (1857: 123).

**Original spelling.***Paludomusfulguratus* Dohrn, 1857.

**Type locality.** “Ceylon” [Sri Lanka].

### *Paludomusfuniculata* Reeve, 1847

**Original reference.**Reeve (1847: pl. 3, fig. 13).

**Original spelling.***Paludomusfuniculatus* Reeve, 1847.

**Type locality.** “In a mountain stream at Ratnapoora, Ceylon” [Sri Lanka].

### *Paludomusfutaii* Gredler, 1889

**Original reference.**Gredler (1889: 159–160).

**Original spelling.***PaludomusFutaii* Gredler, 1889.

**Type locality.** “Im Peho (Kuang-tung)” [China, Guangdong, Peho River].

### *Cyclostomagaillardotii* Bourguignat, 1855

**Original reference.**Bourguignat (1855: 333–334, pl. 8, figs 5–7).

**Original spelling.***CyclostomaGaillardotii* Bourguignat, 1855.

**Type locality.** “Les environs de Sayda, en Syrie” [Syria, Sayda].

### *Paludomusgardneri* Reeve, 1847

**Original reference.**Reeve (1847: pl. 2, fig. 9a, b).

**Original spelling.***PaludomusGardneri* Reeve, 1847.

**Type locality.** “In a stream at the foot of Adam’s Peak, Ceylon” [Sri Lanka, Adam’s Peak].

### *Anceyagiraudi* Bourguignat, 1885

**Original reference.**Bourguignat (1885a: 15–16).

**Type locality.** “Evirons de Mlilo, sur la côte ouest du lac” [Democratic Republic of the Congo, surroundings of Mlilo (= Moliro), western shore of Lake Tanganyika].

### *Syrnolopsisgiraudi* Bourguignat, 1885

**Original reference.**Bourguignat (1885a: 20–21).

**Type locality.** “Plage de Pambété” [Zambia, beach of Pambété, Lake Tanganyika].

### *Bridouxiagiraudi* Bourguignat, 1885

**Original reference.**Bourguignat (1885a: 30).

**Type locality.** “Plage de Kapampa, au sud-ouest du lac” [Democratic Republic of the Congo, beach of Kapampa, western shore of Lake Tanganyika].

### *Baizeagiraudi* Bourguignat, 1885

**Original reference.**Bourguignat (1885a: 34–35).

**Type locality.** “Plage de Kapampa, au sud-ouest du lac, dans la region du Marungu“ [Democratic Republic of the Congo, Marungu region, Kapampa, south-western shore of Lake Tanganyika].

### *Spekiagiraudi* Bourguignat, 1885

**Original reference.**Bourguignat (1885a: 36).

**Type locality.** “À Mpala, sur la côte ouest, ainsi qu’à Pambété, au sud du lac” [Democratic Republic of the Congo, Mpala, western shore of Lake Tanganyika and Zambia, Pambété, southern shore of Lake Tanganyika].

### *Tanganikiagiraudi* Bourguignat, 1885

**Original reference.**Bourguignat (1885a: 44).

**Original spelling.**Tanganikia (Tanganikia) giraudi Bourguignat, 1885.

**Type locality.** “Plage de Kapampa, à l’ouest du lac” [Democratic Republic of the Congo, Kapampa, western shore of Lake Tanganyika].

### *Hauttecoeuriagiraudi* Bourguignat, 1885

**Original reference.**Bourguignat (1885a: 49–50).

**Original spelling.***Hauttecœuria giraudi* Bourguignat, 1885.

**Type locality.** “Sur la plage de Pambété” [Zambia, beach of Pambété, Lake Tanganyika].

### *Limnotrochusgiraudi* Bourguignat, 1885

**Original reference.**Bourguignat (1885a: 59–60).

**Type locality.** “Plage de Pambété” [Zambia, beach of Pambété, Lake Tanganyika].

### *Reymondiagiraudi* Bourguignat, 1885

**Original reference.**Bourguignat (1885a: 65–66).

**Type locality.** “Plage de Mlilo” [Democratic Republic of the Congo, Mlilo (= Moliro), Lake Tanganyika].

### *Paramelaniagiraudi* Bourguignat, 1885

**Original reference.**Bourguignat (1885a: 82).

**Type locality.** “Sur la plage de Mlilo” [Democratic Republic of the Congo, Mlilo (= Moliro), Lake Tanganyika].

### *Stanleyagiraudi* Bourguignat, 1885

**Original reference.**Bourguignat (1885a: 88).

**Type locality.** “Plage de Mlilo” [Democratic Republic of the Congo, Mlilo (= Moliro), Lake Tanganyika].

### *Rumellagiraudi* Bourguignat, 1885

**Original reference.**Bourguignat (1885a: 90–91).

**Type locality.** “Sur la plage de Mpala” [Democratic Republic of the Congo, Lake Tanganyika, beach of Mpala].

### † *Pseudocleopatraglaubrechti* Van Damme & Pickford, 2003

**Original reference.**Van Damme and Pickford (2003: 10–11, 57–59, fig. 47).

**Type locality.** “Kaiso locality A, Lake Albert, Uganda”.

**Type stratum.** “Kyeoro and Kaiso Village Formations, Molluscan Associations G5a and GX, Late Pliocene”.

### *Tanganikiaglobosa* Bourguignat, 1885

**Original reference.**Bourguignat (1885a: 46).

**Original spelling.**Tanganikia (Cambieria) globosa Bourguignat, 1885.

**Type locality.** “Plage de Kapampa” [Democratic Republic of the Congo, beach of Kapampa, western shore of Lake Tanganyika].

### *Rumellaglobosa* Bourguignat, 1888

**Original reference.**Bourguignat (1888: 39–40, pl. 17, figs 20–22).

**Type locality.** “De la côte occidentale” [Democratic Republic of the Congo, western shore of Lake Tanganyika].

### *Hirthiaglobosa* Ancey, 1898

**Original reference.**Ancey (1898: 144–145, pl. 9, fig. H).

**Type locality.** “Habitat in lacu Tanganika, ad partem meridionalem et orientalem «Ufipa» dictam” [Tanzania, Ufipa region, Lake Tanganyika, south-eastern shores].

### *Melaniaglobulosa* Griffith & Pidgeon, 1833

**Original reference.**Griffith and Pidgeon (1833–1834: pl. 14, fig. 6).

**Type locality.** Not given.

**Remarks.** See Remarks under *Melaniaconica* Griffith & Pidgeon, 1833

### *Paludomusgracilis* Brot, 1880 [Unavailable]

**Original reference.**Brot (1880: 41).

**Remarks.** Attributed to Parreyss; published in the synonymy of *Paludomustanjoriensis* Blanford, 1863; unavailable (Code Art. 11.6).

### † *Taeniodomusgracilis* Krause, 1897

**Original reference.**Krause (1897: 213–214, 219, pl. 12, figs 1, 1a, b).

**Type locality.** “Thonmergel vom Sungei Pinoh” [Indonesia, Borneo, Sungai Pinoh].

**Type stratum.** “α Eocaen (der Sandstein Etage) Verbeek‘s” [Eocene, Verbeek’s sandstone stage].

### *Syrnolopsisgracilis* Pilsbry & Bequaert, 1927

**Original reference.**Pilsbry and Bequaert (1927: 234–235, figs 31a–c).

**Type locality.** “Lake Tanganyika”.

### *Paludomusgrandidieri* Crosse & Fischer, 1872

**Original reference.**Crosse and Fischer (1872: 209–210).

**Original spelling.***PaludomusGrandidieri* Crosse & Fischer, 1872.

**Type locality.** “Habitat in rivulis regionis orientalis insulæ Madagascar dictæ” [Madagascar, rivers in the eastern part of the island].

### *Syrnolopsisgrandidieri* Germain, 1905 [Invalid]

**Original reference.**Germain (1905a: 259).

**Original spelling.***SyrnolopsisGrandidieri* Germain, 1905.

**Remarks.** Unjustified emendation of *Syrnolopsisgrandidieriana* Bourguignat, 1885. Germain (1905a) changed the suffix of several species-group names ending in –iana or –ana introduced by Bourguignat (1885, 1888). The names thus formed are to be treated as emendations of the original names (Code Art. 33.2.1) and are available and have as unjustified emendations their own author and date (Code Art. 33.2.2).

### *Spekiagrandidieri* Germain, 1906 [Invalid]

**Original reference.**Germain (1906: 579).

**Original spelling.***SpekiaGrandidieri* Germain, 1906.

**Remarks.** Unjustified emendation of *Spekiagrandidieriana* Bourguignat, 1885. Germain (1906) changed the suffix of several species-group names ending in –iana or –ana introduced by Bourguignat (1885). The names thus formed are to be treated as emendations of the original names (Code Art. 33.2.1) and are available and have as unjustified emendations their own author and date (Code Art. 33.2.2).

### *Syrnolopsisgrandidieriana* Bourguignat, 1885

**Original reference.**Bourguignat (1885a: 18–19).

**Type locality.** “Plage de Pambété” [Zambia, beach of Pambété, Lake Tanganyika].

### *Spekiagrandidieriana* Bourguignat, 1885

**Original reference.**Bourguignat (1885a: 40–41).

**Type locality.** “Sur les plages du Marungu, sur la côte sud-ouest du lac“ [Democratic Republic of the Congo, beaches of Marungu region, south-western shores of Lake Tanganyika].

### *Giraudiagrandidieriana* Bourguignat, 1885

**Original reference.**Bourguignat (1885a: 63–64).

**Type locality.** “Dans le sable d’une plage au nord de Mlilo” [Democratic Republic of the Congo, on a beach north of Mlilo (= Moliro), Lake Tanganyika].

### *Paramelaniagrandidieriana* Bourguignat, 1885

**Original reference.**Bourguignat (1885a: 70–71).

**Type locality.** “Aux environs de Pambété” [Zambia, surroundings of Pambété, Lake Tanganyika].

### *Tiphobianassagrandis* Smith, 1881

**Original reference.**Smith (1881b: 561).

**Original spelling.**Tiphobia (Paramelania) nassavar.grandis Smith, 1881.

**Type locality.** “Lake Tanganyika”.

### *Cleopatraguillemei* Bourguignat, 1885

**Original reference.**Bourguignat (1885c: 6–8).

**Original spelling.***CleopatraGuillemei* Bourguignat, 1885.

**Type locality.** “Nyanza Oukéwéré, près de la Mission française”[Tanzania, Lake Victoria].

### *Cleopatraguillemeti* Bourguignat, 1888

**Original reference.**Bourguignat (1888: 13–14, pl. 4, fig. 4).

**Original spelling.***CleopatraGuillemeti* Bourguignat, 1888.

**Type locality.** “L’embouchure du Malagarazi” [Tanzania, Malagarasi Delta].

### *Nassopsisguillemei* Martel & Dautzenberg, 1899

**Original reference.**Martel and Dautzenberg (1899: 172–173, pl. 8, figs 12, 13).

**Original spelling.***NassopsisGuillemei* Martel & Dautzenberg, 1899.

**Type locality.** “Lac Tanganyika” [Lake Tanganyika].

### *Paramelaniaimperialisguillemei* Martel & Dautzenberg, 1899

**Original reference.**Martel and Dautzenberg (1899: 178–180, pl. 8, fig. 23).

**Original spelling.***Paramelaniaimperialis* var. *Guillemei* Martel & Dautzenberg, 1899.

**Type locality.** “Lac Tanganyika” [Lake Tanganyika].

### *Spekiahamyi* Germain, 1906 [Invalid]

**Original reference.**Germain (1906: 580).

**Original spelling.***SpekiaHamyi* Germain, 1906.

**Remarks.** Unjustified emendation of *Spekiahamyana* Bourguignat, 1885. Germain (1906) changed the suffix of several species-group names ending in –iana or –ana introduced by Bourguignat (1885). The names thus formed are to be treated as emendations of the original names (Code Art. 33.2.1) and are available and have as unjustified emendations their own author and date (Code Art. 33.2.2).

### *Syrnolopsishamyana* Bourguignat, 1885

**Original reference.**Bourguignat (1885a: 17–18).

**Type locality.** “Sur la plage de Pambété” [Zambia, beach of Pambété, Lake Tanganyika].

### *Spekiahamyana* Bourguignat, 1885

**Original reference.**Bourguignat (1885a: 38–39).

**Type locality.** “Sur la plage de Mpala” [Democratic Republic of the Congo, Lake Tanganyika, beach of Mpala].

### *Hauttecoeuriahamyana* Bourguignat, 1885

**Original reference.**Bourguignat (1885a: 48–49).

**Original spelling.***Hauttecœuria hamyana* Bourguignet, 1885.

**Type locality.** “Sur la plage de Pambété” [Zambia, beach of Pambété, Lake Tanganyika].

### *Paramelaniahamyana* Bourguignat, 1885

**Original reference.**Bourguignat (1885a: 71–72).

**Type locality.** “De la plage de Pambété, au sud du lac” [Zambia, beach of Pambété, southern shore of Lake Tanganyika].

### *Paludomushanleyi* Dohrn, 1858

**Original reference.**Dohrn (1858: 535).

**Type locality.** “Inhabitants of mountain-streams in Ceylon” [Sri Lanka].

### *Cleopatrahargeri* Smith, 1908

**Original reference.**Smith (1908: 13).

**Original spelling.***CleopatraHargeri* Smith, 1908.

**Type locality.** “Kalingwisi River, Lake Mweru” [Zambia].

### *Cleopatrahemmingi* Verdcourt, 1956

**Original reference.**Hemming and Verdcourt (1956: 60–62, figs 3, 4e).

**Type locality.** “Italian Somaliland, 18 miles W. of Beles Cogani, frequent in the large shallow pools” [Somalia].

### *Paludomushilberi* Gredler, 1886

**Original reference.**Gredler (1886: 19–20).

**Original spelling.***Paludomus* (?) *Hilberi* Gredler, 1886.

**Type locality.** “Aus Hensan, der Provinz Hunan” [China, Hunan].

### *Cleopatrahirta* Dautzenberg & Germain, 1914

**Original reference.**Dautzenberg and Germain (1914: 59, pl. 4, figs 11–14).

**Type locality.** “Stn. 49, Nyangwe, Lualaba” [Democratic Republic of the Congo, Nyangwe, Lualaba River].

### *Tiphobiahorei* Smith, 1880

**Original reference.**Smith (1880a: 348–349, pl. 31, fig. 7).

**Type locality.** “Lake Tanganyika”.

### *Melaniahorei* Smith, 1880

**Original reference.**Smith (1880b: 427).

**Original spelling.***Melania* (―?) *Horei* Smith, 1880.

**Type locality.** “Lake Tanganyika”.

### *Bathanaliahowesi* Moore, 1898

**Original reference.**Moore (1898a: 452, fig. 2; 1898c: 192, 203, pl. 12, figs 29, 30).

**Original spelling.***BathanaliaHowesi* Moore, 1898.

**Type locality.** “Near Mleroes, Tanganyika, at a depth of 950 ft” [Zambia, near Mleroes, southern shore of Lake Tanganyika].

**Remarks.** The name *BathanaliaHowesi* was published simultaneously (March 1898) in two separate works by J. E. S. Moore (1998a, c). In Moore (1898a) the name was spelled *BathanaliaHowesii*, and in Moore (1898b) it was spelled *BathanaliaHowesi*. Moore (1898d) used *BathanaliaHowesii* and *Bathanalia Howsei*, while in another subsequent work by the author, Moore (1898e), only the name *BathanaliaHowesi* was used. Therefore, Moore (1898e) is deemed here as First Reviser under the Code Art. 24.2.4 of the simultaneously published spellings of Moore (1898a, c) and *Bathanaliahowesi* Moore, 1998 (including obligate corrections under the Code Art. 32.5.2.5) is regarded as the correct original spelling. *Bathanalia Howsei* is an incorrect subsequent spelling by Moore (1898d).

### *Bourguignatiaimperialis* Giraud, 1885

**Original reference.**Giraud (1885: 194).

**Type locality.** “Région du Marungu, sur la plage de Mpala (côte sud-ouest du Tanganika)“ [Democratic Republic of the Congo, Lake Tanganyika, Marungu region, beach of Mpala, south-western shore of Lake Tanganyika].

### *Paludomusinflata* Brot, 1880

**Original reference.**Brot (1880: 44–45, pl. 5, fig. 10).

**Original spelling.***Paludomusinflatus* Brot, 1880.

**Type locality.** “Travancore” and “Amerghat” [India, Kerala; Amerghat].

### *Paramelaniainfralirata* Bourguignat, 1888

**Original reference.**Bourguignat (1888: 35–36, pl. 15, figs 4, 5).

**Type locality.** “Lac Tanganika” [Lake Tanganyika].

### *Bythocerasiridescens* Moore, 1898

**Original reference.**Moore (1898a: 452, fig. 1).

**Type locality.** “Near Sumbu, Tanganyika, at a depth of 680 ft” [Zambia, near Sumbu, southern part of Lake Tanganyika].

### *Paludomusisseli* Brot, 1880

**Original reference.**Brot (1880: 31–32, pl. 7, figs 7, 7a, 8).

**Original spelling.***PaludomusIsseli* Brot, 1880.

**Type locality.** “Sarawak auf Borneo” [Malaysia, Borneo, Sarawak].

### *Paludomusconicajaintiaca* Nevill, 1884

**Original reference.**Nevill (1884: 289).

**Original spelling.**Paludomusconicavar.jaintiaca Nevill, 1884.

**Type locality.** “S. Jaintia Hills” [India, Meghalaya].

**Remarks.** See Remarks under *Paludomusconicacherraensis* Nevill, 1884.

### *Cleopatrajohnstoni* Smith, 1893

**Original reference.**Smith (1893: 637, pl. 59, fig. 9).

**Type locality.** “Lake Mweru” [Zambia/Democratic Republic of the Congo].

### *Tiphobiajouberti* Bourguignat, 1886

**Original reference.**Bourguignat (1886: 146–148, 150, pl. 6, figs 11–13).

**Type locality.** “Sur les plages du Masanzé au N.-O. de la presqu’île Oubouari“ [Democratic Republic of the Congo, north-western part of the Ubwari Peninsula, beaches of Masanzé, Lake Tanganyika].

### *Cleopatrajouberti* Bourguignat, 1888

**Original reference.**Bourguignat (1888: 13–14, pl. 4, fig. 3).

**Original spelling.***CleopatraJouberti* Bourguignat, 1888.

**Type locality.** “L’embouchure du Malagarazi” [Tanzania, Malagarasi Delta].

### *Cambieriajouberti* Bourguignat, 1888

**Original reference.**Bourguignat (1888: 17–18, pl. 6, figs 15, 16).

**Original spelling.***CambieriaJouberti* Bourguignat, 1888.

**Type locality.** “Lac Tanganika” [Lake Tanganyika].

### *Hauttecoeuriajouberti* Bourguignat, 1888

**Original reference.**Bourguignat (1888: 19, pl. 7, figs 24, 25).

**Original spelling.***Hauttecœuria Jouberti* Bourguignat, 1888.

**Type locality.** “Lac Tanganika” [Lake Tanganyika].

### *Reymondiajouberti* Bourguignat, 1888

**Original reference.**Bourguignat (1888: 27–28, pl. 11, figs 5, 6).

**Original spelling.***ReymondiaJouberti* Bourguignat, 1888.

**Type locality.** “Lac Tanganika” [Lake Tanganyika].

### *Bourguignatiajouberti* Bourguignat, 1888

**Original reference.**Bourguignat (1888: 29, pl. 12, figs 5–7).

**Original spelling.***BourguignatiaJouberti* Bourguignat, 1888.

**Type locality.** “Sur les plages de la côte occidentale” [Democratic Republic of the Congo, western shore of Lake Tanganyika].

### *Lavigeriajouberti* Bourguignat, 1888

**Original reference.**Bourguignat (1888: 33–34, pl. 14, fig. 4).

**Original spelling.***LavigeriaJouberti* Bourguignat, 1888.

**Type locality.** “Sur les rives nord et surtout occidentales du lac” [Democratic Republic of the Conga, Lake Tanganyika, northern and western shores].

### *Rumellajouberti* Bourguignat, 1888

**Original reference.**Bourguignat (1888: 40, pl. 17, figs 29–31).

**Original spelling.***RumellaJouberti* Bourguignat, 1888.

**Type locality.** “De la côte occidentale” [Democratic Republic of the Congo, western shore of Lake Tanganyika].

### *Paludomustanjoriensiskadapaensis* Nevill, 1884

**Original reference.**Nevill (1884: 294).

**Original spelling.**Paludomustanjoriensisvar.kadapaensis Nevill, 1884.

**Type locality.** “Kadapa District, Madras” [India, Tamil Nadu, Chennai].

**Remarks.** See Remarks under *Paludomusconicacherraensis* Nevill, 1884.

### † *Cleopatrakaisoensis* Van Damme & Pickford, 2003

**Original reference.**Van Damme and Pickford (2003: 11, 52–53, fig. 38).

**Type locality.** “Kaiso Village locality Kaiso A, Lake Albert, Uganda”.

**Type stratum.** “Kaiso Village Formation, Molluscan Associations GX, Late Pliocene”.

### *Paludomuskamaingia* Rao, 1929

**Original reference.**Rao (1929: 290–292).

**Type locality.** “From a small rocky stream near Kamaing” [Myanmar, Kamaing].

### *Cleopatransendweensiskatangana* Pilsbry & Bequaert, 1927

**Original reference.**Pilsbry and Bequaert (1927: 296, fig. 55b).

**Type locality.** “Luvua River, between Kiambi and Ankoro” [Democratic Republic of the Congo].

### *Limnotrochuskirkii* Smith, 1880

**Original reference.**Smith (1880b: 426).

**Original spelling.***LimnotrochusKirkii* Smith, 1880.

**Type locality.** “Lake Tanganyika”.

**Remarks.***Limnotrochuskerkii* as used by Moore (1903) is an incorrect spelling. The deliberate change by Smith (1881a: 286–287) to *Limnotrochuskirki* has also to be interpreted as an incorrect subsequent spelling (Code Art. 33.4).

### † *Potadomoideskisegiensis* Van Damme & Pickford, 2003

**Original reference.**Van Damme and Pickford (2003: 10, 17, 47–48, 62, fig. 34).

**Type locality.** “Kisegi locality KI 5, Lake Albert, Uganda”.

**Type stratum.** “Kakara Formation, Molluscan Association G1, Late Miocene”.

### *Paludomusconicakopiliensis* Nevill, 1884

**Original reference.**Nevill (1884: 289).

**Original spelling.**Paludomusconicavar.kopiliensis Nevill, 1884.

**Type locality.** “Kopili River, North Assam [and] North Assam” [India, Meghalaya/Assam].

**Remarks.** See Remarks under *Paludomusconicacherraensis* Nevill, 1884.

### *Bithyniabulimoideskotschyana* Frauenfeld, 1864 [Unavailable]

**Original reference.**Frauenfeld (1864: 583, 619).

**Original spelling.***Bythiniabulimoides* var. *Kotschyana* Frauenfeld, 1864.

**Remarks.** Nomen nudum, name introduced without definition, description, or indication (Code Art. 12.1).

### *Paludomuskweichowensis* Cheng, 1937

**Original reference.** Cheng (1937: 445–447, figs 1.3, 2.3).

**Original spelling.**Paludomus (Hemimitra) kweichowensis Cheng, 1937.

**Type locality.** “At Shih-men-kan, Kweichow Province, China” [China, Guizhou].

### *Cleopatrakynganica* Bourguignat, 1879

**Original reference.**Bourguignat (1879: 21).

**Original spelling.***CleopatraKynganica* Bourguignat, 1879.

**Type locality.** “Dans le fleuve Kyngani, près de Bagamoyo” [Tanzania, Bagamoyo, Kyngani River].

**Remarks.***Cleopatra Kinganica* is an incorrect subsequent spelling by Bourguignat (1885b, c).

### *Paludomuslabiosa* Benson, 1856

**Original reference.**Benson (1856: 495–496).

**Type locality.** “Hab. in rivulis vallis Tenasserim” [Myanmar, rivers in the Tenasserim Valley].

### *Paludomuslacunoides* Aldrich, 1889

**Original reference.**Aldrich (1889: 23–24, pl. 3, figs 1, 1a–c).

**Type locality.** “In the Kusan and Penggiron districts in Southeastern Borneo and from the Kinan and Kiwa Rivers” [Indonesia, Borneo].

### *Paramelanialacunosa* Bourguignat, 1888

**Original reference.**Bourguignat (1888: 37–38, pl. 16, figs 5, 6).

**Type locality.** “Lac Tanganika” [Lake Tanganyika].

### *Syrnolopsislacustris* Smith, 1880

**Original reference.**Smith (1880b: 426–427).

**Type locality.** “Lake Tanganyika”.

### *Paludomuslaevis* Layard, 1855

**Original reference.**Layard (1855: 89).

**Original spelling.***Paludomuslævis* Layard, 1855.

**Type locality.** “Ceylon, in slow-running streams on the northern side of the mountain zone extending into the flat country beyond Anarajahpoora [and] in a paddy field in the south of the island, near the village of Heneratgodde” [Sri Lanka].

### *Cleopatralangi* Pilsbry & Bequaert, 1927

**Original reference.**Pilsbry and Bequaert (1927: 293–294, figs 51a–d, 52).

**Type locality.** “Stanleyville” [Democratic Republic of the Congo, Kisangani].

### *Cleopatralaurenti* Bourguignat, 1879

**Original reference.**Bourguignat (1879: 24).

**Original spelling.***CleopatraLaurenti* Bourguignat, 1879.

**Type locality.** “Sur les bords du Nil, au-dessus du Caire“ [Egypt, Nile River, upriver of Cairo].

### *Hauttecoeurialavigeriana* Bourguignat, 1888

**Original reference.**Bourguignat (1888: 19, pl. 7, figs 26–27).

**Original spelling.***Hauttecœuria Lavigeriana* Bourguignat, 1888.

**Type locality.** “Lac Tanganika” [Lake Tanganyika].

### *Giraudialavigeriana* Bourguignat, 1888

**Original reference.**Bourguignat (1888: 27–28, pl. 11, figs 22–24).

**Original spelling.***GiraudiaLavigeriana* Bourguignat, 1888.

**Type locality.** “Lac Tanganika” [Lake Tanganyika].

### *Rumellalavigeriana* Bourguignat, 1888

**Original reference.**Bourguignat (1888: 40, pl. 17, figs 32–34).

**Original spelling.***RumellaLavigeriana* Bourguignat, 1888.

**Type locality.** “De la côte occidentale” [Democratic Republic of the Congo, western shore of Lake Tanganyika].

### *Paludomuslayardi* Reeve, 1852

**Original reference.**Reeve (1852: 128).

**Original spelling.***PaludomusLayardi* Reeve, 1852.

**Type locality.** “Mountain streams of Ceylon” [Sri Lanka].

### *Lavigerialechaptoisi* Ancey, 1898

**Original reference.**Ancey (1898: 145–146, pl. 9, fig. I).

**Original spelling.***Lavigeria* (?) *lechaptoisi* Ancey, 1898.

**Type locality.** “Habitat in ripa meridionali lacus Tanganika «Ufipa» dicta” [Tanzania, Ufipa region, Lake Tanganyika].

### *Paramelanialedoulxiana* Bourguignat, 1885

**Original reference.**Bourguignat (1885a: 80–81).

**Type locality.** “Sur la plage Kapampa, à l’ouest du lac” [Democratic Republic of the Congo, Kapampa, western shore of Lake Tanganyika].

**Remarks.** The name was attributed to V. Giraud by Bourguignat (1885a: 80), but Bourguignat alone was responsible for the description and therefore is the author of the name (Code Art 50.1).

### † *Viviparuslepersonnei* Gautier, 1970

**Original reference.** Gautier & Charig (1970: 41, 95–96, figs 6a–d).

**Original spelling.**Viviparus?lepersonnei Gautier, 1970.

**Type locality.** “Kaiso“ [Uganda, Kaiso].

**Type stratum.** Kaiso, Site 3.

### *Cleopatralesnei* Germain, 1935

**Original reference.**Germain (1935: 68–69).

**Original spelling.***CleopatraLesnei* Germain, 1935.

**Type locality.** “Bas Sangadzé (Zambèse)” [Mozambique, Sofala].

**Remarks.** Type locality given erroneously; according to Brown and Mandahl-Barth (1989) a *Paludomus* Swainson, 1840 species.

### *Paramelanialessepsiana* Bourguignat, 1885

**Original reference.**Bourguignat (1885a: 78–79).

**Type locality.** “Sur la plage de Mlilo” [Democratic Republic of the Congo, Mlilo (= Moliro), Lake Tanganyika].

**Remarks.** The name was attributed to V. Giraud by Bourguignat (1885a: 78), but Bourguignat alone was responsible for the description and therefore is the author of the name (Code Art 50.1).

### *Cleopatraletourneuxi* Bourguignat, 1879

**Original reference.**Bourguignat (1879: 19–21).

**Original spelling.***CleopatraLetourneuxi* Bourguignat, 1879.

**Type locality.** “Fleuve Kyngani, près de Bagamoyo” [Tanzania, Bagamoyo, Kyngani River].

### *Ponsonbyaleucoraphe* Ancey, 1890

**Original reference.**Ancey (1890: 347).

**Type locality.** “Lac Tanganika” [Lake Tanganyika].

### *Hauttecoeurialevesquei* Germain, 1905 [Invalid]

**Original reference.**Germain (1905a: 258).

**Original spelling.***Hauttecœuria Levesquei* Germain, 1905.

**Remarks.** Unjustified emendation of *Hauttecœuria levesquiana* Bourguignat, 1888. Germain (1905a) changed the suffix of several species-group names ending in –iana or –ana introduced by Bourguignat (1885, 1888). The names thus formed are to be treated as emendations of the original names (Code Art. 33.2.1) and are available and have as unjustified emendations their own author and date (Code Art. 33.2.2).

### *Hauttecoeurialevesquiana* Bourguignat, 1888

**Original reference.**Bourguignat (1888: 21–22, pl. 8, figs 9–11).

**Original spelling.***Hauttecœuria Levesquiana* Bourguignat, 1888.

**Type locality.** “Lac Tanganika” [Lake Tanganyika].

### *Cleopatralhotellerii* Bourguignat, 1879

**Original reference.**Bourguignat (1879: 25).

**Original spelling.***CleopatraLhotellerii* Bourguignat, 1879.

**Type locality.** “Mandara, près d’Alexandrie” [Egypt, Mandara near Alexandria].

### † *Pseudocleopatralikeae* Van Damme & Pickford, 2003

**Original reference.**Van Damme and Pickford (2003: 10, 18, 54–55, 59, 63, figs 41a, 42).

**Type locality.** “Sinda-Mohari Gautier Site 13, Semliki Plain, Congo” [Democratic Republic of the Congo].

**Type stratum.** “Mohari and Kisegi Formations, Molluscan Association G0 & G1, Late and Middle Miocene”.

### *Syrnolopsislacustrislilacina* Dartevelle & Schwetz, 1948

**Original reference.**Dartevelle and Schwetz (1948: 30, 37, 39, 52, 59, 74, 85, pl. 1, fig. 14).

**Original spelling.**Syrnolopsislacustrisvar.lilacina Dartevelle & Schwetz, 1948.

**Type locality.** “Lac Tanganilsa (?) (Stn. 2065)” [Lake Tanganyika].

### *Paramelanialimnaea* Bourguignat, 1888

**Original reference.**Bourguignat (1888: 39–40, pl. 17, figs 7, 8).

**Original spelling.***ParamelaniaLimnæa* Bourguignat, 1888.

**Type locality.** “Lac Tanganika” [Lake Tanganyika].

### *Paludinalineata* Menke, 1828 [Unavailable]

**Original reference.**Menke (1828: 23).

**Remarks.** Nomen nudum, introduced without description, definition, or indication (Code Art. 12.1). Only the name *Helixtanschaurica* Gmelin, 1791 was mentioned. Presented in the same way in Menke (1830: 41).

### *Edgarialittoralis* Bourguignat, 1888

**Original reference.**Bourguignat (1888: 33–34, pl. 14, figs 14–16).

**Type locality.** “Çà et là sur tout le pourtour du lac” [Lake Tanganyika].

### *Hirthialittorina* Ancey, 1898

**Original reference.**Ancey (1898: 142–144, pl. 9, fig. G).

**Type locality.** “Habitat in lacu Tanganika, ad partem meridionalem et orientalem «Ufipa» dictam” [Tanzania, Ufipa region, south-eastern shore of Lake Tanganyika].

### *Paramelanialivingstoniana* Bourguignat, 1885

**Original reference.**Bourguignat (1885a: 85–86).

**Type locality.** “Plages de Pambété et de Kapampa ” [Zambia, beach of Pambété, and Democratic Republic of the Congo, Kapampa, Lake Tanganyika].

**Remarks.** The name was attributed to V. Giraud by Bourguignat (1885a: 85), but Bourguignat alone was responsible for the description and therefore is the author of the name (Code Art. 50.1).

### *Hauttecoeurialocardi* Germain, 1905 [Invalid]

**Original reference.**Germain (1905a: 258).

**Original spelling.***Hauttecœuria Locardi* Germain, 1905.

**Remarks.** Unjustified emendation *Hauttecœurialocardiana* Bourguignat, 1888. Germain (1905a) changed the suffix of several species-group names ending in –iana or –ana introduced by Bourguignat (1885, 1888). The names thus formed are to be treated as emendations of the original names (Code Art. 33.2.1) and are available and have as unjustified emendations their own author and date (Code Art. 33.2.2).

### *Paramelanialocardi* Germain, 1905 [Invalid]

**Original reference.**Germain (1905a: 259).

**Original spelling.***ParamelaniaLocardi* Germain, 1905.

**Remarks.** Unjustified emendation of *Paramelanialocardiana* Bourguignat, 1885. Germain (1905a) changed the suffix of several species-group names ending in –iana or –ana introduced by Bourguignat (1885, 1888). The names thus formed are to be treated as emendations of the original names (Code Art. 33.2.1) and are available and have as unjustified emendations their own author and date (Code Art. 33.2.2).

### *Paramelanialocardiana* Bourguignat, 1885

**Original reference.**Bourguignat (1885a: 82–83).

**Type locality.** “Sur la plage de Pambété” [Zambia, beach of Pambété, Lake Tanganyika].

### *Hauttecœurialocardiana* Bourguignat, 1888

**Original reference.**Bourguignat (1888: 21–22, pl. 8, figs 12–14).

**Original spelling.***Hauttecœuria Locardiana* Bourguignat, 1888.

**Type locality.** “Lac Tanganika” [Lake Tanganyika].

### *Tiphobialongirostris* Bourguignat, 1886

**Original reference.**Bourguignat (1886: 144–146, 150, pl. 6, figs 8–10).

**Type locality.** “Sur la plage de Kibanga, au sud de la presqu’île Oubouari” [Democratic Republic of the Congo, Kibanga, southern part of the Obwari Peninsula, Lake Tanganyika].

### *Paludomusloricata* Reeve, 1847

**Original reference.**Reeve (1847: pl. 1, figs 1a–c).

**Original spelling.***Paludomusloricatus* Reeve, 1847.

**Type locality.** “In rapids flowing from Adam’s Peak, Ceylon” [Sri Lanka, near Adam’s Peak].

### *Cleopatrabulimoideslutea* Pallary, 1909 [Unavailable]

**Original reference.**Pallary (1909: 64).

**Original spelling.***Cleopatra bulimoïdes* var. ex colore *lutea* Pallary, 1909.

**Remarks.** Nomen nudum, introduced without description, definition, or indication (Code Art. 12.1).

### *Paludomuslutea* Adams, 1874

**Original reference.**Adams (1874: 585–586, pl. 59, figs 5, 5a).

**Original spelling.***Paludomusluteus* Adams, 1874.

**Type locality.** “Borneo”.

### *Paludinalutosa* Souleyet, 1852

**Original reference.**Souleyet (1852: 550, pl. 31, figs 28–30).

**Type locality.** “Cette espèce provient du Gange” [India/Bangladesh, Ganges River].

### *Paramelaniamabilliana* Bourguignat, 1888

**Original reference.**Bourguignat (1888: 37–38, pl. 16, figs 17–18).

**Original spelling.***ParamelaniaMabilliana* Bourguignat, 1888.

**Type locality.** “Lac Tanganika” [Lake Tanganyika].

### *Hauttecoeuriamacrostoma* Bourguignat, 1888

**Original reference.**Bourguignat (1888: 19, pl. 7, figs 10–11).

**Original spelling.***Hauttecœuria macrostoma* Bourguignat, 1888.

**Type locality.** “Lac Tanganika” [Lake Tanganyika].

### *Paludomusmaculata* Lea, 1856

**Original reference.**Lea (1856: 110).

**Type locality.** “Ahmednugger, India” [India, Maharashtra].

### *Paludinamadagascariensis* Crosse & Fischer, 1872

**Original reference.**Crosse and Fischer (1872: 210).

**Original spelling.***PaludinaMadagascariensis* Crosse & Fischer, 1872.

**Type locality.** “Habitat in rivulis regionis orientalis insulæ Madagascar dictæ” [Madagascar, rivers in the eastern part of the island].

### *Paludomusmadagascariensis* Brot, 1880

**Original reference.**Brot (1880: 48, pl. 8, fig. 7).

**Type locality.** “Madagascar” [Madagascar].

**Original spelling.***PaludomusMadagascariensis* Brot, 1880.

### *Paludomushanleyimajor* Nevill, 1884

**Original reference.**Nevill (1884: 305).

**Original spelling.***Paludomus* [*Tanalia*] *hanleyi*var.major Nevill, 1884.

**Type locality.** “Ceylon” [Sri Lanka].

**Remarks.** See Remarks under *Paludomusconicacherraensis* Nevill, 1884.

### *Paramelaniastanleyanamajor* Bourguignat, 1885

**Original reference.**Bourguignat (1885a: 75).

**Original spelling.**Paramelaniastanleyanavar.major Bourguignat, 1885.

**Type locality.** “Plages de Pambété et de Mlilo“ [Zambia, beach of Pambété and Democratic Republic of the Congo, Mlilo (= Moliro), Lake Tanganyika].

### *Syrnolopsisminutamajor* Germain, 1905

**Original reference.**Germain (1905a: 260).

**Original spelling.**Syrnolopsisminutavar.major Germain, 1905.

**Type locality.** “Lac Tanganika“ [Lake Tanganyika].

### *Cleopatrabulimoidesmajor* Dautzenberg & Germain, 1914

**Original reference.**Dautzenberg and Germain (1914: 56–57).

**Original spelling.**Cleopatrabulimoidesf.major Dautzenberg & Germain, 1914.

**Type locality.** “Stn. 72, Kibombo, 4° lat. S“ [Democratic Republic of the Congo, Kibombo].

### *Edgarianassamajor* Leloup, 1953 [Unavailable]

**Original reference.**Leloup (1953: 166).

**Original spelling.**Edgarianassaf.major Leloup, 1953.

**Remarks.** Mentioned in the synonymy of *Edgardianassa* forme *typica*; unavailable (Code Art. 11.6).

### *Edgarialocardianamajor* Leloup, 1953 [Unavailable]

**Original reference.**Leloup (1953: 167).

**Original spelling.**Edgarialocardianaf.major Leloup, 1953.

**Remarks.** Mentioned in the synonymy of *Edgardianassa* forme *giraudi*; unavailable (Code Art. 11.6).

### *Paludomustanjoriensismalabarica* Nevill, 1884

**Original reference.**Nevill (1884: 294–295).

**Original spelling.**Paludomustanjoriensisvar.malabarica Nevill, 1884.

**Type locality.** “Travancore [and] Pulney Hills” [India, Kerala, Pulney Hills].

**Remarks.** See Remarks under *Paludomusconicacherraensis* Nevill, 1884.

### *Cleopatramangoroensis* Ancey, 1890

**Original reference.**Ancey (1890: 344–345).

**Type locality.** “Fleuve Mangoro, de Tananarive à la côte orientale de Madagascar, à 700 m” [Madagascar, from Antananarivo to the eastern coast, at 700 m, Mangoro River].

### *Cleopatramareotica* Bourguignat, 1879

**Original reference.**Bourguignat (1879: 25).

**Original spelling.***CleopatraMareotica* Bourguignat, 1979.

**Type locality.** “Dans le petit canal de Mustapha, près de Ramlé (Égypte)” [Egypt, Ramle near Alexandria, Mustapha Canal].

### *Hauttecoeuriamaunoiri* Germain, 1905 [Invalid]

**Original reference.**Germain (1905a: 258).

**Original spelling.***Hauttecœuria Maunoiri* Germain, 1905.

**Remarks.** Unjustified emendation of *Hauttecoeuriamaunoiriana* Bourguignat, 1885. Germain (1905a) changed the suffix of several species-group names ending in –iana or –ana introduced by Bourguignat (1885, 1888). The names thus formed are to be treated as emendations of the original names (Code Art. 33.2.1) and are available and have as unjustified emendations their own author and date (Code Art. 33.2.2).

### *Tanganikiamaunoiriana* Bourguignat, 1885

**Original reference.**Bourguignat (1885a: 44–45).

**Original spelling.**Tanganikia (Cambieria) maunoiriana Bourguignat, 1885.

**Type locality.** “Plages de Mpala et de Pambété” [Democratic Republic of the Congo, Lake Tanganyika, beach of Mpala and Zambia, beach of Pambété, southern shore of Lake Tanganyika].

### *Hauttecoeuriamaunoiriana* Bourguignat, 1885

**Original reference.**Bourguignat (1885a: 55).

**Original spelling.***Hauttecœuria maunoiriana* Bourguignat, 1885.

**Type locality.** “Sur la plage de Pambété” [Zambia, beach of Pambété, Lake Tanganyika].

### *Paludomusmaurus* Reeve, 1852

**Original reference.**Reeve (1852: 127).

**Type locality.** “Branch of the Ganges” [India/Bangladesh, Ganges River].

### *Cleopatrabulimoidesmedia* Pallary, 1924

**Original reference.**Pallary (1924: 33).

**Original spelling.***Cleopatrabulimoides* variété *media* Pallary, 1924.

**Type locality.** “Égypte” [Egypt].

### *Paludomusmelanostoma* Hanley & Theobald, 1876

**Original reference.**Hanley and Theobald (1876: 49, pl. 121, figs 8–9).

**Type locality.** “Ceylon” [Sri Lanka].

### *Cremnoconchusmessageri* Bavay & Dautzenberg, 1900

**Original reference.**Bavay and Dautzenberg (1900: 116–117).

**Original spelling.***CremnoconchusMessageri* Bavay & Dautzenberg, 1900.

**Type locality.** “Rivière Song-Ky-Kong, près de That-Khé” [Vietnam, near Thất Khê, Kỳ Cùng River].

### † *Ellinoriamichelae* Van Damme & Pickford, 2003

**Original reference.**Van Damme and Pickford (2003: 10, 61–62, fig. 50).

**Type locality.** “Nkondo locality NK 34, Lake Albert, Uganda”.

**Type stratum.** “Nkondo Formation, Molluscan Association G3b, Late Miocene to Early Pliocene”.

### *Paludomusmicrosculpta* Nevill, 1884 [Unavailable]

**Original reference.**Nevill (1884: 297).

**Type locality.** “Ceylon” [Sri Lanka].

**Remarks.** Introduced without description, definition, or indication (Code Art. 12.1).

### *Paludomusrotundamicrostoma* Nevill, 1884

**Original reference.**Nevill (1884: 295).

**Original spelling.**Paludomusrotundavar.microstoma Nevill, 1884.

**Type locality.** “Anamallay Rivers [and] Madura Hills” [India, Tamil Nadu].

**Remarks.** See Remarks under *Paludomusconicacherraensis* Nevill, 1884.

### *Tanganikiasolutamilneedwardsi* Germain, 1905 [Invalid]

**Original reference.**Germain (1905a: 258).

**Original spelling.***Tanganikia* (*Hauttecœuria*) *soluta* var. *Milne*-*Edwardsi* Germain, 1905.

**Remarks.** Unjustified emendation of *Hauttecœuria milneedwardsiana* Bourguignat, 1885. Germain (1905a) changed the suffix of several species-group names ending in –iana or –ana introduced by Bourguignat (1885, 1888). The names thus formed are to be treated as emendations of the original names (Code Art. 33.2.1) and are available and have as unjustified emendations their own author and date (Code Art. 33.2.2).

### *Hauttecoeuriamilneedwardsiana* Bourguignat, 1885

**Original reference.**Bourguignat (1885a: 50–51).

**Original spelling.***Hauttecœuriamilne-edwardsiana* Bourguignat, 1885.

**Type locality.** “Sur les plages du sud du lac” [Zambia, southern shores of Lake Tanganyika].

### *Paramelaniamilneedwardsiana* Bourguignat, 1885

**Original reference.**Bourguignat (1885a: 77–78).

**Original spelling.***Paramelaniamilne-edwardsiana* Bourguignat, 1885.

**Type locality.** “Plage de Mlilo” [Democratic Republic of the Congo, Mlilo (= Moliro), Lake Tanganyika].

### *Rumellamilneedwardsiana* Bourguignat, 1885

**Original reference.**Bourguignat (1885a: 91).

**Original spelling.***Rumellamilne-edwardsiana* Bourguignat, 1885.

**Type locality.** “Plage de Kapampa, au sud-ouest du lac” [Democratic Republic of the Congo, beach of Kapampa, western shore of Lake Tanganyika].

### *Paludomusminensis* Cheng, 1937

**Original reference.** Cheng (1937: 445–448, figs 1.4, 2.4).

**Type locality.** “In Min River near Kienway, Szechuan Province, China”.

### *Giraudiaminima* Smith, 1908

**Original reference.**Smith (1908: 12).

**Type locality.** “Lake Tanganyika”.

### *Paludomusregulataminor* Nevill, 1884 [Unavailable]

**Original reference.**Nevill (1884: 291).

**Original spelling.**Paludomusregulatasubvar.minor Nevill, 1884.

**Type locality.** “Burma” [Myanmar].

**Remarks.** See Remarks under *Paludomusconicacherraensis* Nevill, 1884; not subsequently made available under the Code (Art. 45.6.4.1).

### *Paludomussulcataminor* Nevill, 1884 [Unavailable]

**Original reference.**Nevill (1884: 299).

**Original spelling.***Paludomus* [*Philopotamis*] *sulcata*subvar.minor Nevill, 1884.

**Type locality.** “Ceylon” [Sri Lanka].

**Remarks.** See Remarks under *Paludomusconicacherraensis* Nevill, 1884. Used by Rao (1929) as the valid name of a taxon, however without definition, description, or indication. Therefore not adopted as the name of a taxon in the sense of the code and thus not made available (Code Art. 45.6.4.1).

### *Paludomusaculeataminor* Nevill, 1884 [Unavailable]

**Original reference.**Nevill (1884: 302).

**Original spelling.***Paludomus* [*Tanalia*] *aculeata*subvar.minor Nevill, 1884.

**Type locality.** “Ceylon” [Sri Lanka].

**Remarks.** See Remarks under *Paludomusconicacherraensis* Nevill, 1884. Not subsequently made available under the Code (Art. 45.6.4.1).

### *Paramelaniaduveyrierianaminor* Bourguignat, 1885

**Original reference.**Bourguignat (1885a: 79).

**Original spelling.**Paramelaniaduveyrierianavar.minor Bourguignat, 1885.

**Type locality.** “Dans les environs de Pambété” [Zambia, surroundings of Pambété, Lake Tanganyika].

**Remarks.** Acting as first revisers, we here select the name Paramelaniaduveyrierianavar.minor Bourguignat, 1885 to have the precedence over Paramelanialocardianavar.minor Bourguignat, 1885.

### *Paramelanialocardianaminor* Bourguignat, 1885 [Invalid]

**Original reference.**Bourguignat (1885a: 83).

**Original spelling.**Paramelanialocardianavar.minor Bourguignat, 1885.

**Type locality.** “À Mlilo et aussi à Pambété” [Democratic Republic of the Congo, Mlilo (= Moliro) and Zambia, Pambété, Lake Tanganyika].

**Remarks.** Invalid, homonym of Paramelaniaduveyrierianavar.minor Bourguignat, 1885 [first reviser action, see under *Paramelaniaduveyrierianaminor* Bourguignat, 1885].

### *Reymondiaminor* Smith, 1889

**Original reference.**Smith (1889: 174).

**Type locality.** “Lake Tanganyika”.

### *Hauttecoeuriagiraudiminor* Bourguignat, 1890

**Original reference.**Bourguignat (1890: 100).

**Original spelling.**Hauttecœuria Giraudi var.minor Bourguignat, 1890.

**Type locality.** “A Kibanga, au sud de la presqu’île Oubouari” [Democratic Republic of the Congo, Kibanga, Obwari Peninsula, Lake Tanganyika].

### *Reymondiagiraudiminor* Bourguignat, 1890 [Invalid]

**Original reference.**Bourguignat (1890: 155).

**Original spelling.***ReymondiaGiraudi* forme *minor* Bourguignat, 1890.

**Type locality.** “Plage au nord de Mlilo; sur une autre plage voisine” [Democratic Republic of the Congo, beach north of Mlilo (= Moliro), Lake Tanganyika].

**Remarks.** Invalid, junior homonym of *Reymondiaminor* Smith, 1889.

### *Bythocerasminor* Moore, 1903

**Original reference.**Moore (1903: 242, 244, fig. 24).

**Type locality.** “Lake Tanganyika”.

### *Lavigeriajoubertiminor* Germain, 1905 [Unavailable]

**Original reference.**Germain (1905a: 259).

**Original spelling.**Lavigeriajoubertivar.minor Germain, 1905.

**Type locality.** Not given.

**Remarks.** Nomen nudum, introduced without description, definition, or indication (Code Art. 12.1).

### *Giraudiabridouxianaminor* Ancey, 1906 [Unavailable]

**Original reference.**Ancey (1906b: 254).

**Original spelling.***GiraudiaBridouxiana*f.minor Ancey, 1906.

**Type locality.** “Ufipa, M’bwé” [Tanzania, Ufipa region, Lake Tanganyika].

**Remarks.** Nomen nudum, introduced without description, definition, or indication (Code Art. 12.1).

### *Cleopatrajohnstoniminor* Dautzenberg & Germain, 1914 [Unavailable]

**Original reference.**Dautzenberg and Germain (1914: 57).

**Original spelling.**CleopatraJohnstonif.minor Dautzenberg & Germain, 1914.

**Type locality.** “Stn. 38, riv. Luvua, entre Ankoro et Kiambi“ and “Stn. 79, riv. Luvua, entre Ankoro et Kiambi” [Democratic Republic of the Congo, between Ankoro and Kiambi, Luvua River].

**Remarks.** Nomen nudum, introduced without description, definition, or indication (Code Art. 12.1).

### *Paludomusferrugineaminor* Dautzenberg & Germain, 1914 [Unavailable]

**Original reference.**Dautzenberg and Germain (1914: 61).

**Original spelling.**Paludomus (Zanguebaria) ferrugineaf.minor Dautzenberg & Germain, 1914.

**Type locality.** “Stn. 36, Lovoi, Kikondja, Katanga“ [Democratic Republic of the Congo, near Kikondja, Lovoi River].

**Remarks.** Nomen nudum, introduced without description, definition, or indication (Code Art. 12.1).

### *Cleopatrabulimoidesminor* Pallary, 1924

**Original reference.**Pallary (1924: 33).

**Original spelling.***Cleopatrabulimoides* variété *minor* Pallary, 1924.

**Type locality.** “Égypte” … “d’une localité inconnue” [Egypt].

### *Edgariamilneedwardsianaminor* Dartevelle & Schwetz, 1948

**Original reference.**Dartevelle and Schwetz (1948: 31, 37, 43, 53, 60, 74, 76).

**Type locality.** “Ufipa” [Tanzania, Ufipa region, Lake Tanganyika], see Ancey (1906b: 263).

**Original spelling.***Edgariamilne*-*edwardsiana*var.minor Dartevelle & Schwetz, 1948.

**Remarks.**Dartevelle and Schwetz (1948: 53) remarked “D’autre part, nous avons ulilisé la variété suivante, due à C. F. Ancey et restée manuscrite, mais signalée (2, p. 263): Edgariamilne-edwardsianavar.minor C. F. Ancey”. Ancey (1906b: 263) stated that the specimens are distinguished from the nominate form by their smaller size, thus providing a diagnosis, which is referred to by Dartevelle and Schwetz (1948).

### *Edgariaegregiaminor* Leloup, 1953 [Unavailable]

**Original reference.**Leloup (1953: 166).

**Original spelling.**Edgariaegregiaf.minor Leloup, 1953.

**Remarks.** Mentioned in the synonymy of *Edgardianassa* forme *typica*; unavailable (Code Art. 11.6).

### *Edgarianassaminor* Leloup, 1953 [Unavailable]

**Original reference.**Leloup (1953: 166).

**Original spelling.**Edgarianassaf.minor Leloup, 1953.

**Remarks.** Mentioned in the synonymy of *Edgardianassa* forme *typica*; unavailable (Code Art. 11.6).

### *Edgarialocardianaspinulosaminor* Leloup, 1953 [Unavailable]

**Original reference.**Leloup (1953: 167).

**Original spelling.***Edgarialocardiana var. spinulosa*f.minor Leloup, 1953.

**Remarks.** Mentioned in the synonymy of *Edgardianassa* forme *giraudi*; unavailable (Code Art. 11.6).

### *Syrnolopsisminuta* Bourguignat, 1885

**Original reference.**Bourguignat (1885a: 21–22).

**Type locality.** “Plage de Pambété” [Zambia, beach of Pambété, Lake Tanganyika].

**Remarks.***Syrnolopsisminima* Bourguignat, 1885 as used by Bandel, 1998 is a lapsus for *Syrnolopsisminuta* Bourguignat, 1885.

### *Hauttecoeuriaminuta* Bourguignat, 1885

**Original reference.**Bourguignat (1885a: 57–58).

**Original spelling.***Hauttecœuria minuta* Bourguignat, 1885.

**Type locality.** “Sur la plage de Mpala” [Democratic Republic of the Congo, Lake Tanganyika, beach of Mpala].

### *Paludomusminutiuscula* Gredler, 1885

**Original reference.**Gredler (1885: 232–233, pl. 6, fig. 8).

**Original spelling.**Paludomus?minutiusculus Gredler, 1885.

**Type locality.** “Aus Tschin-chi, einer Stadt 3. Ranges in der Provinz Kuei-tscheu” [China, Guizhou].

### *Philopotamismisconceptus* Nevill, 1884 [Unavailable]

**Original reference.**Nevill (1884: 299).

**Remarks.** Mentioned as a manuscript name under *Paludomusnigricans* Reeve, 1847 by Nevill (1884: 299); unavailable (Code Art. 11.6).

### *Melaniamodicella* Lea & Lea, 1851

**Original reference.**Lea and Lea (1851: 196–197).

**Type locality.** “Timor” [Timor].

### *Hauttecoeuriamoineti* Bourguignat, 1888

**Original reference.**Bourguignat (1888: 19, pl. 7, figs 1–3).

**Original spelling.***Hauttecœuria Moineti* Bourguignat, 1888.

**Type locality.** “Lac Tanganika” [Lake Tanganyika].

### *Syrnolopsislacustrismolirensis* Pilsbry & Bequaert, 1927

**Original reference.**Pilsbry and Bequaert (1927: 230–231, figs 26h, 27c–d).

**Type locality.** “Off Moliro, in 70 m and at Moliro; in Lake Tanganyika” [Democratic Republic of the Kongo, off and at Moliro, Lake Tanganyika].

### *Reymondiamonceti* Bourguignat, 1888

**Original reference.**Bourguignat (1888: 27–28, pl. 11, figs 7–8).

**Original spelling.***ReymondiaMonceti* Bourguignat, 1888.

**Type locality.** “Lac Tanganika” [Lake Tanganyika].

### *Edgariamonceti* Bourguignat, 1888

**Original reference.**Bourguignat (1888: 33–34, pl. 14, figs 12–13).

**Original spelling.***EdgariaMonceti* Bourguignat, 1888.

**Type locality.** “Cà et là sur tout le pourtour du lac” [Lake Tanganyika].

### *Paludomusmonile* Hanley & Theobald, 1876

**Original reference.**Hanley and Theobald (1876: 44, pl. 108, fig. 10).

**Type locality.** “Southern India”.

### *Paludomusmoreleti* Issel, 1874

**Original reference.**Issel (1874: 456–457, pl. 7, fig. 21–22).

**Original spelling.***PaludomusMoreleti* Issel, 1874.

**Type locality.** “Territorio di Sarawak” [Malaysia, Borneo, Sarawak].

### *Cleopatramorelli* Preston, 1905

**Original reference.**Preston (1905: 300–301, fig. 3).

**Original spelling.***CleopatraMorelli* Preston, 1905.

**Type locality.** “Just above Victoria Falls, Zambesi River” [Zimbabwe, Zambesi River, above Victoria Falls].

### *Paramelaniaimperialismpalaensis* Martel & Dautzenberg, 1899

**Original reference.**Martel and Dautzenberg (1899: 180, pl. 8, fig. 24).

**Original spelling.**Paramelaniaimperialisvar.mpalaensis Martel & Dautzenberg, 1899.

**Type locality.** “Lac Tanganyika” [Democratic Republic of the Congo, Lake Tanganyika, Mpala].

### *Cleopatra.mterizensis* Melvill & Standen, 1907

**Original reference.**Melvill and Standen (1907: 5, pl. 1, fig. 2).

**Type locality.** “Bed Mterize River, a tributary of the Loangwa River” [Zambia].

### *Syrnolopsisminutamulticarinata* Ancey, 1906

**Original reference.**Ancey (1906b: 267).

**Original spelling.**Syrnolopsisminutaf.multicarinata Ancey, 1906.

**Type locality.** “Lac Tanganika, côte orientale” [Democratic Republic of the Congo, western shore of Lake Tanganyika].

### *Cleopatramultilirata* Ancey, 1906

**Original reference.**Ancey (1906a: 45).

**Type locality.** “Vinaninony, Madagascar” [Madagascar, Vinaninony].

### *Bithyniamultisulcata* Bourguignat, 1888

**Original reference.**Bourguignat (1888: 11, pl. 3, figs 7–8).

**Original spelling.***Bythiniamultisulcata* Bourguignat, 1888.

**Type locality.** “Sur les rives de la presqu’île Oubouari” [Democratic Republic of the Congo, Lake Tanganyika, shores of Obwari Peninsula].

### *Syrnolopsisminutamulticsulcata* Ancey, 1906 [Unavailable]

**Original reference.**Ancey (1906b: 255).

**Original spelling.**Syrnolopsisminutaf.multisulcata Ancey, 1906.

**Type locality.** “Lac Tanganika, côte orientale” [Democratic Republic of the Congo, western shore of Lake Tanganyika].

**Remarks.** Nomen nudum, introduced without description, definition, or indication (Code Art. 12.1).

### † *Pseudocleopatramusiimei* Van Damme & Pickford, 2003

**Original reference.**Van Damme and Pickford (2003: 10, 12–13, 55–58, 63, 66, fig. 43).

**Type locality.** “Nkondo locality NK 122, Lake Albert, Uganda”.

**Type stratum.** “Lusso Formation (Biozone L0) (L. Edward), Nkondo Formation (L. Albert), Molluscan Associations G3a, G3b & G3c. Late Miocene to Early Pliocene”.

### *Cleopatramweruensis* Smith, 1893

**Original reference.**Smith (1893: 637–638, pl. 59, fig. 10).

**Type locality.** “Lake Mweru” [Democratic Republic of the Congo/Uganda].

### *Paludomusandersonianamyadoungensis* Nevill, 1881

**Original reference.**Nevill (1881: 160).

**Original spelling.**PaludomusAndersonianavar.myadoungensis Nevill, 1881.

**Type locality.** “Myadoung, near the Yunnan” [Myanmar].

### *Paludomusandersoniananana* Nevill, 1881

**Original reference.**Nevill (1881: 160).

**Original spelling.**PaludomusAndersonianasubvar.nana Nevill, 1881.

**Type locality.** “Also from Pegu” [Myanmar].

**Remarks.** Introduced as a subvariety. However, made available by Rao (1929: 292–293, text fig. 6) (Code Art. 45.6.4.1).

### *Paludomusconicanana* Nevill, 1884 [Invalid]

**Original reference.**Nevill (1884: 289).

**Original spelling.**Paludomusconicavar.nana Nevill, 1884.

**Type locality.** “W. Khasi Hills” [India, Meghalaya].

**Remarks.** See Remarks under *Paludomusconicacherraensis* Nevill, 1884. Made available by Kobelt in Pfeffer and Kobelt (1887: 422) (Code Art. 45.6.4.1). Junior homonym of *Paludomusandersoniananana* Nevill, 1881.

### *Melanianassa* Woodward, 1859

**Original reference.**Woodward (1859: 349, pl. 47, fig. 4).

**Original spelling.**Melania (Melanella) nassa Woodward, 1859.

**Type locality.** “Lake Tanganyika in Central Africa”.

**Remarks.***Nassopsisnana* as used by Moore (1903) is an incorrect spelling.

### *Paramelanianassatella* Bourguignat, 1888

**Original reference.**Bourguignat (1888: 37–38, pl. 16, figs 3, 4).

**Type locality.** “Lac Tanganika” [Lake Tanganyika].

### *Paramelanianassatiformis* Bourguignat, 1888

**Original reference.**Bourguignat (1888: 39–40, pl. 17, figs 5, 6).

**Type locality.** “Lac Tanganika” [Lake Tanganyika].

### *Paludomusnasuta* Dohrn, 1857

**Original reference.**Dohrn (1857: 123–124).

**Original spelling.***Paludomusnasuta* Dohrn, 1857.

**Type locality.** “Ceylon” [Sri Lanka].

### *Lithoglyphusneritinoides* Smith, 1880

**Original reference.**Smith (1880b: 426).

**Type locality.** “Lake Tanganyika”.

**Remarks.***StanleyaNeritoides* is an incorrect subsequent spelling by Bourguignat (1885, 1888).

### *Paludomusneritoides* Reeve, 1847

**Original reference.**Reeve (1847: pl. 1, fig. 3a. b).

**Original spelling.***PaludomusNeritoides* reeve, 1847.

**Type locality.** “In the bed of a river at Ambegamoa, Ceylon” [Sri Lanka].

### *Bithyniabulimoidesnigra* Frauenfeld, 1864

**Original reference.**Frauenfeld (1864: 583, 629).

**Original spelling.***Bythiniabulimoides* var. *Nigra* Frauenfeld, 1864.

**Type locality.** “Aegypten” [Egypt].

**Remarks.** Attributed to Caillaud. Introduced by Frauenfeld (1864: 583) as a variety of *Bithyniabulimoides* (Olivier, 1804), and on p. 629 the name was mentioned with some descriptional terms; thus satisfying the Code (Art. 12.1).

### *Paludomusnigricans* Reeve, 1847

**Original reference.**Reeve (1847: pl. 2, fig. 6).

**Type locality.** “Ceylon (in mountain streams at 6,000 feet elevation)” [Sri Lanka].

### *Paludomusnodulosa* Dohrn, 1857

**Original reference.**Dohrn (1857: 125).

**Original spelling.***Paludomusnodulosus* Dohrn, 1857.

**Type locality.** “Ceylon” [Sri Lanka].

### *Cleopatrabulimoidesnsendweensis* Dupuis & Putzeys, 1901

**Original reference.**Dupuis and Putzeys (1901: 55–56).

**Original spelling.**Cleopatrabulimoidesvar.nsendweensis Dupuis & Putzeys, 1901.

**Type locality.** “Nyangwe, Nsendwe, Lokandu” [Democratic Republic of the Congo, Nyangwe; Nsendwe; Lokandu].

### *Cleopatranyanzae* Mandahl-Barth, 1954

**Original reference.**Mandahl-Barth (1954: 63, fig. 29a–b).

**Type locality.** “Kanyakwar wells” [Kenya].

### *Melaniaobesa* Philippi, 1847

**Original reference.**Philippi (1846–1847: 170, pl. 4, fig. 3).

**Original spelling.**Melania?obesa Philippi, 1847.

**Type locality.** “Nova Hollandia?” [Australia; doubtful].

### *Anceyagiraudiobesa* Leloup, 1953 [Unavailable]

**Original reference.**Leloup (1953: 108).

**Original spelling.***Anceyagiraudi* variété *obesa* Leloup, 1953.

**Remarks.** Mentioned in the synonymy of Anceya (Anceya) giraudi; unavailable (Code Art. 11.6).

### Edgarianassavar.obliqua Ancey, 1906

**Original reference.**Ancey (1906b: 264).

**Type locality.** “Sur les rivages de l’Ufipa” [Tanzania, Ufipa region, Lake Tanganyika].

### *Cleopatraobscura* Mandahl-Barth, 1968

**Original reference.**Mandahl-Barth (1968: 34–35, pl. 1 fig. 8, pl. 7 figs 1–3).

**Type locality.** ”Kafubu region, Kimilolo River at Keyberg” [Democratic Republic of the Congo, Keyberg Station near Lubumbashi, Kimililo River].

### *Paramelaniaobtusa* Bourguignat, 1888

**Original reference.**Bourguignat (1888: 35–36, pl. 15, figs 6–8).

**Type locality.** “Lac Tanganika” [Lake Tanganyika].

### *Paludomusolivacea* Reeve, 1847

**Original reference.**Reeve (1847: pl. 1, fig. 5).

**Original spelling.***Paludomusolivaceus* Reeve, 1847.

**Type locality.** “Point Palmas, Island of Sumatra (in a muddy stream)” [Indonsesia, Sumatra, Point Palmas; doubtful].

### *Tanganikiaopalina* Bourguignat, 1888

**Original reference.**Bourguignat (1888: 15, pl. 5, figs 18–19).

**Type locality.** “Sur les plages de la partie septentrionale du lac“ [Democratic Republic of the Congo/Burundi, northern shores of Lake Tanganyika].

### *Paludomusornata* Benson, 1856

**Original reference.**Benson (1856: 496).

**Type locality.** “Hab. in regno Burmanico” [Myanmar].

### *Tanganikiaovoidea* Bourguignat, 1885

**Original reference.**Bourguignat (1885a: 45–46).

**Original spelling.**Tanganikia (Cambieria) ovoidea Bourguignat, 1885.

**Type locality.** “Plage de Kapampa, au sud-ouest du lac” [Democratic Republic of the Congo, beach of Kapampa, western shore of Lake Tanganyika].

### *Paludomuspalawanica* Brot, 1891

**Original reference.**Brot (1891: 17–18).

**Type locality.** “In a brook about ten miles from Puerto Princesa in the Island of Palawan, Philippine Archipelago” [Philippines].

**Remarks.**Brot (1891: 17–18) used both, the spelling *P.palawanicus* and *P.palawanica* in the original description. The grammatical gender of the genus-group name *Paludomus* is feminine. Therefore, *Paludomuspalawanica* Brot, 1891 is the correct original spelling.

### *Bithyniabulimoidespallida* Frauenfeld, 1864

**Original reference.**Frauenfeld (1864: 583, 633).

**Original spelling.***Bythiniabulimoides* var. *Pallida* Frauenfeld, 1864.

**Type locality.** “Aegypten” [Egypt].

**Remarks.** Attributed to Caillaud. Introduced by Frauenfeld (1864: 583) as a variety of *Bithyniabulimoides* (Olivier, 1804), and on p. 633 the name was mentioned with some descriptional terms; thus satisfying the Code (Art. 12.1).

### *Paludomuspaludinoides* Reeve, 1852

**Original reference.**Reeve (1852: 127).

**Original spelling.***PaludomusPaludinoides* Reeve, 1852.

**Type locality.** “Sikkim branch of the Ganges” [India/Bangladesh].

### *Paludomuspalustris* Layard, 1855

**Original reference.**Layard (1855: 89–90).

**Type locality.** “The grassy margins of a tank at Anarajahpoora” [Sri Lanka].

### *Paramelaniapalustris* Bourguignat, 1888

**Original reference.**Bourguignat (1888: 36, pl. 15, figs 31, 32).

**Type locality.** “Lac Tanganika” [Lake Tanganyika].

### *Paludomusparvula* Rao, 1929

**Original reference.**Rao (1929: 293–294).

**Type locality.** “From an unknown locality in Burma” [Myanmar].

### *Paludomusparva* Layard, 1855

**Original reference.**Layard (1855: 90).

**Original spelling.***Paludomusparvus* Layard, 1855.

**Type locality.** “Bombay” [India, Maharashtra, Mumbai].

### *Tiphobianassapaucicostata* Smith, 1881

**Original reference.**Smith (1881b: 561).

**Original spelling.**Tiphobia (Paramelania) nassavar.paucicostata Smith, 1881.

**Type locality.** “Lake Tanganyika”.

### *Cleopatrapauli* Bourguignat, 1885

**Original reference.**Bourguignat (1885b: 27–28, pl. 1, fig. 3).

**Original spelling.***CleopatraPauli* Bourguignat, 1885.

**Type locality.** “Bords du fleuve Haouach” [Chad, Haouach River].

### *Paludomusconicapealiana* Nevill, 1884

**Original reference.**Nevill (1884: 288).

**Original spelling.**Paludomusconicavar.pealiana Nevill, 1884.

**Type locality.** “Assam [and] Sibsagar” [India, Assam, Sivasagar].

**Remarks.** See Remarks under *Paludomusconicacherraensis* Nevill, 1884.

### *Paludomusandersonianapeguensis* Nevill, 1877

**Original reference.**Nevill (1877: 35).

**Original spelling.***PaludomusAndersoniana* var. *Peguensis* Nevill, 1877.

**Type locality.** “Pegu” [Myanmar].

### *Potadomoidespelseneeri* Leloup, 1953

**Original reference.**Leloup (1953: 92, 102–105, 115, 132, 136, pl. 3, fig. 6, figs. 43, 47, 57P, 72H, 76).

**Type locality.** “Dans le delta de la Malagarasi, le long des rives et dans les petites baies, coquilles; par tamisage de la vase et des débris végétaux recueillis par la petite drague dans une anse calme et encombrée de végétations, – 30–40 cm, spécimens vivants” [Tanzania, Malagarasi Delta].

### *Cleopatrapercarinata* Bourguignat, 1885

**Original reference.**Bourguignat (1885b: 27–28, pl. 1, fig. 1).

**Type locality.** “Lac Haoussa”.

### *Lavigeriaperexemia* Bourguignat, 1888

**Original reference.**Bourguignat (1888: 33–34, pl. 14, fig. 3).

**Type locality.** “Sur les rives nord et surtout occidentales du lac” [Democratic Republic of the Congo, northern and western shores of Lake Tanganyika].

### *Viviparaamiltonipersolida* Issel, 1874

**Original reference.**Bourguignat (1888: 454–455).

**Original spelling.***ViviparaHamiltoni*var.persolida Issel, 1874.

**Type locality.** “Sadong, in limpidi ruscelli” [Malaysia, Borneo, Sarawak, Sadong River].

### *Paludinapetrosa* Gould, 1843

**Original reference.**Gould (1843: 144).

**Type locality.** “Tavoy” [Myanmar, Dawei].

### *Paludomusphasianinus* Reeve, 1852

**Original reference.**Reeve (1852: 127).

**Original spelling.***Paludomusphasianina* Reeve, 1852.

**Type locality.** “Seychelles”.

### *Paludomuspicta* Reeve, 1847

**Original reference.**Reeve (1847: pl. 2, figs 10a–b).

**Original spelling.***Paludomuspictus* Reeve, 1847.

**Type locality.** “In a mountain stream at Ratnapoora, Ceylon” [Sri Lanka].

### *Cleopatrapilula* Mandahl-Barth, 1967

**Original reference.**Mandahl-Barth (1967: 130, pl. 2, fig. 12).

**Type locality.** “Bushimaie River at Lukuta, Kasai” [Democratic Republic of the Congo, Kasai, Lukuta, Mbuji-Mayi River].

### *Cleopatrapirothi* Jickeli, 1881

**Original reference.**Jickeli (1881: 338).

**Original spelling.***CleopatraPirothi* Jickeli, 1881.

**Type locality.** “Nordost-Afrika” [Northeast Africa].

### Syrnolopsislacustrisvar.pluricarinata Dartevelle & Schwetz, 1948

**Original reference.**Dartevelle and Schwetz (1948: 30, 37, 39, 52, 59, 74, 85, pl. 1, fig. 11).

**Original spelling.**Syrnolopsislacustrisvar.pluricarinata Dartevelle & Schwetz, 1948.

**Type locality.** “Lac Tanganika, baie de Kasakalawe, profondeur : 10 a 15 m (Stn. 2068)” [Zambia, Bay of Kaskalawe, Lake Tangayika].

### *Rissoaponsonbyi* Smith, 1889

**Original reference.**Smith (1889: 175).

**Original spelling.***Rissoa (Horea) Ponsonbyi* Smith, 1889.

**Type locality.** “Lake Tanganyika”.

### *Cleopatrapoutrini* Germain, 1909

**Original reference.**Germain (1909: 377–378).

**Original spelling.***CleopatraPoutrini* Germain, 1909.

**Type locality.** ”Dans l’Egueï, à 1,000 kilomètres environ au nord de Fort-Lamy” [Chad, Kanem, Eguei].

### *Giraudiapraeclara* Bourguignat, 1885

**Original reference.**Bourguignat (1885a: 62–63).

**Original spelling.***Giraudiapræclara* Bourguignat, 1885.

**Type locality.** “Plage au nord de Mlilo” [Democratic Republic of the Congo, beach north of Mlilo (= Moliro), Lake Tanganyika].

### *Paramelaniapulchella* Bourguignat, 1885

**Original reference.**Bourguignat (1885a: 86).

**Type locality.** “Plage de Kapampa” [Democratic Republic of the Congo, beach of Kapampa, western shore of Lake Tanganyika].

### *Cleopatrabulimoidespulchella* Pallary, 1909

**Original reference.**Pallary (1909: 63).

**Original spelling.***Cleopatrabulimoïdes*var.pulchella Pallary, 1909.

**Type locality.** “Égypte” [Egypt].

**Remarks.** Attributed to Bourguignat in Pallary (1924: 33).

### *Paludomuspunctata* Reeve, 1852

**Original reference.**Reeve (1852: 127).

**Original spelling.***Paludomuspunctatus* Reeve, 1852.

**Type locality.** “Mauritius”.

### *Syrnolopsispupoidea* Leloup, 1953 [Unavailable]

**Original reference.**Leloup (1953: 121).

**Remarks.** Mentioned in the synonymy of *Syrnolopsisgracilis* as a manuscript name of Dautzenberg & Dupuis; unavailable (Code Art. 11.6).

### *Hauttecoeuriapusilla* Bourguignat, 1888

**Original reference.**Bourguignat (1888: 22, pl. 8, figs 32–34).

**Original spelling.***Hauttecœuria pusilla* Bourguignat, 1888.

**Type locality.** “Lac Tanganika” [Lake Tanganyika].

### *Paludomuspustulosa* Annandale, 1921

**Original reference.**Annandale (1921: 563–564, fig. 11A, B).

**Type locality.** “From one stream in the south part of the Manipur Valley” [India, Manipur].

### *Reymondiapyramidalis* Bourguignat, 1888

**Original reference.**Bourguignat (1888: 27–28, pl. 11, figs 9–13).

**Type locality.** “Lac Tanganika” [Lake Tanganyika].

### *Paludomuspyriformis* Dohrn, 1858

**Original reference.**Dohrn (1858: 536).

**Type locality.** “Inhabitants of mountain-streams in Ceylon” [Sri Lanka].

### *Paludomusqianensis* Liu, Duan & Zhang, 1994

**Original reference.**Liu et al. (1994: 30, 34).

**Type locality.** “Huangguoshu, (25.9°N, 105.6°E), Guizhou Province […] on the stones of the ditch beside river” [China, Guizhou].

### *Paludomusquadrasi* Moellendorff, 1894

**Original reference.** Quadras & Moellendorff (1894: 130).

**Original spelling.**Paludomus (Philopotamis) quadrasi Moellendorff, 1894.

**Type locality.** “Busuanga” [Philippines, Busuanga Island].

**Remarks.** Authorship attributed to Moellendorff (Code Art. 50.1).

### *Syrnolopsisquintana* Mabille, 1901

**Original reference.**Mabille (1901: 56).

**Type locality.** “Lac Tanganika” [Lake Tanganyika].

### *Paramelaniarandabeli* Bourguignat, 1888

**Original reference.**Bourguignat (1888: 37–38, pl. 16, figs 21, 22).

**Original spelling.***ParamelaniaRandabeli* Bourguignat, 1888.

**Type locality.** “Lac Tanganika” [Lake Tanganyika].

### *Paludomusrapaeformis* Brot, 1880

**Original reference.**Brot (1880: 30–31, pl. 5, fig. 10).

**Type locality.** Not given.

### *Cleopatraraymondi* Bourguignat, 1879

**Original reference.**Bourguignat (1879: 23–24).

**Original spelling.***CleopatraRaymondi* Bourguignat, 1879.

**Type locality.** “Lac Ballat dans l’isthme de Suez” [Egypt, Isthmus of Suez].

### *Tanaliareevei* Layard, 1855

**Original reference.**Layard (1855: 92).

**Original spelling.***TanaliaReevei* Layard, 1855.

**Type locality.** “The Calloo ganga, Ratnapoora” [Sri Lanka, Ratnapura, Kalu River].

### *Philopotamisregalis* Layard, 1855

**Original reference.**Layard (1855: 93).

**Type locality.** “Stream in the Cnia Corle, Western province, Ceylon” [Sri Lanka].

### *Paludomusregulata* Benson, 1856

**Original reference.**Benson (1856: 496).

**Type locality.** “Hab. ad Thyet-Myo Burmanorum” [Myanmar, Thayet].

### *Paludomusreticulata* Blanford, 1870

**Original reference.**Blanford (1870: 9–10, pl. 3, fig. 1).

**Type locality.** “Hab. in Cachar” [India, Assam, Cachar].

### *Melaniaretusa* Griffith & Pidgeon, 1833

**Original reference.**Griffith and Pidgeon (1833–1834: pl. 14, fig. 9).

**Type locality.** Not given.

**Remarks.** See Remarks under *Melaniaconica* Griffith & Pidgeon, 1833

### *Paludomusretusa* Swainson, 1840

**Original reference.**Swainson (1840: 340).

**Original spelling.**Paludomus (Hemimitra) retusa Swainson, 1840.

**Type locality.** Not given.

### *Bridouxiareymondi* Bourguignat, 1885

**Original reference.**Bourguignat (1885a: 32–33).

**Type locality.** “Plage de Kapampa” [Democratic Republic of the Congo, beach of Kapampa, western shore of Lake Tanganyika].

**Remarks.** The name was attributed to V. Giraud by Bourguignat (1885a: 32), but Bourguignat alone was responsible for the description and is therefore the author of the name (Code Art. 50.1).

### *Spekiareymondi* Bourguignat, 1885

**Original reference.**Bourguignat (1885a: 39–40).

**Type locality.** “Sur la plage de Mpala” [Democratic Republic of the Congo, Lake Tanganyika, beach of Mpala].

**Remarks.** The name was attributed to V. Giraud by Bourguignat (1885a: 39), but Bourguignat alone was responsible for the description and therefore is the author of the name (Code art. 50.1).

### *Hauttecoeuriareymondi* Bourguignat, 1885

**Original reference.**Bourguignat (1885a: 54–55).

**Original spelling.***Hauttecœuria reymondi* Bourguignat, 1885.

**Type locality.** Not given.

**Remarks.** The name was attributed to V. Giraud by Bourguignat (1885a: 54), but Bourguignat alone was responsible for the description and therefore is the author of the name (Code Art. 50.1).

### *Paramelaniareymondi* Bourguignat, 1885

**Original reference.**Bourguignat (1885a: 72–73).

**Type locality.** “Sur la plage de Kapampa dans le Marungu (côte sud-ouest du lac)“ [Democratic Republic of the Congo, Marungu region, beach of Kapampa, western shore of Lake Tanganyika].

**Remarks.** The name was attributed to V. Giraud by Bourguignat (1885a: 72), but Bourguignat alone was responsible for the description and therefore is the author of the name (Code Art. 50.1).

### *Cleopatrabulimoidesrichardi* Germain, 1911

**Original reference.**Germain (1911: 200, pl. 2, figs 5, 6).

**Original spelling.***Cleopatrabulimoides* var. *Richardi* Germain, 1911.

**Type locality.** “Intérieur du Tchad, à 40 kilomètres du bord Ouest” [Interior of Lake Chad, 40 km from its western shore] and “Zone sableuse, bord occidental du Tchad” [Lake Chad, western shore].

### *Paludomusrotunda* Blanford, 1870

**Original reference.**Blanford (1870: 10, pl. 3, fig. 2).

**Type locality.** “Hab. in regione Travancorica” [India, Kerala].

### *Stanleyarotunda* Smith, 1904

**Original reference.**Smith (1904: 93).

**Type locality.** Given as “[Lac Tanganika], côte orientale” [Lake Tanganyika, eastern shore] in Bourguignat (1888) and as “sur les plages entre Oudjiji et l’embouchure du Malagarazi” [Tanzania, Lake Tanganyika, beaches between Ujiji and Malagarasi Delta] in Bourguignat (1890).

**Remarks.** Name introduced without description, but with reference to descriptions in Bourguignat (1888, 1890).

### † *Pseudocleopatrarotunda* Van Damme & Pickford, 2003

**Original reference.**Van Damme and Pickford (2003: 10, 58, 63, 66, fig. 46).

**Type locality.** “Nkondo locality NK 120, Lake Albert, Uganda”.

**Type stratum.** “Nkondo Formation, Molluscan Association G3b, Terminal Miocene-Early Pliocene”.

### *Paludomusrotungensis* Preston, 1915

**Original reference.**Preston (1915a: 539–540).

**Type locality.** “Tenmalai, Upper Rotung” [India, Arunachal Pradesh].

### *Paludomusrudis* Reeve, 1852

**Original reference.**Reeve (1852: 126).

**Type locality.** Not given.

### *Lavigeriaruellaniana* Bourguignat, 1888

**Original reference.**Bourguignat (1888: 33–34, pl. 14, figs 5, 6).

**Original spelling.***LavigeriaRuellaniana* Bourguignat, 1888.

**Type locality.** “Sur les rives nord et surtout occidentales du lac” [Democratic Republic of the Congo, northern and western shores of Lake Tanganyika].

**Remarks.***Lavigeria Ruelliana* is an incorrect subsequent spelling by Martel and Dautzenberg (1899).

### *Anceyarufocincta* Smith, 1906

**Original reference.**Smith (1906: 183–184, pl. 10, fig. 12).

**Type locality.** “Kirando, towards south end of the east coast, 10 fath” [Tanzania, Kirando, Lake Tanganyika, eastern shore]

### *Lithoglyphusrufofilosus* Smith, 1880

**Original reference.**Smith (1880b: 426).

**Type locality.** “Lake Tanganyika”.

### *Cleopatrapirothirufolirata* Germain, 1919

**Original reference.**Germain (1919a: 119).

**Original spelling.***Cleopatrapirothi* variété *rufolirata* Germain, 1919.

**Type locality.** “Rhodésie septentrionale: Lealui, sur le Haut-Zambèse” [Zambia, Lealui].

### *Cleopatrarugosa* Connolly, 1925

**Original reference.**Connolly (1925: 424–425).

**Type locality.** “Italian Somaliland; Aggherrar” [Somalia, Aggherrar].

### † *Edgariarukwaensis* Cox, 1939

**Original reference.**Cox (1939: 246–247, pl. 15, figs 6, 7).

**Type locality.** “Rungwa Sleeping Sickness Concentration Arealies about, lies about eighty miles to the north-west of the present shore of the lake” [Tanzania, Rungwa].

**Type stratum.** “Quaternary deposits of Lake Rukwa”.

### *Paludomusrusiostoma* Gredler, 1885

**Original reference.**Gredler (1885: 231–232, pl. 6, figs 7, 7a).

**Type locality.** “Aus Tschin-chi, einer Stadt 3. Ranges in der Provinz Kuei-tscheu” [China, Guizhou].

**Remarks.** Probably a viviparid, see Zilch (1974).

### *Cleopatraschoutedeni* Dautzenberg & Germain, 1914

**Original reference.**Dautzenberg and Germain (1914: 58–59, pl. 4, figs 15, 16).

**Original spelling.***CleopatraSchoutedeni* Dautzenberg & Germain, 1914.

**Type locality.** “Stn. 50, Nyangwe, Lualaba” [Democratic Republic of the Congo, Nyangwe, Lualaba River], through the lectotype designation by Glaubrecht and Strong (2007: 387).

### *Syrnolopsisminutasemilaevis* Ancey, 1906

**Original reference.**Ancey (1906b: 255, 267).

**Original spelling.**Syrnolopsisminutaf.semilaevis Ancey, 1906.

**Type locality.** “Lac Tanganika, côte orientale” [Tanzania/Burundi, eastern shore of Lake Tanganyika.

### *Paludinasenegalensis* Morelet, 1860

**Original reference.**Morelet (1860a: 190).

**Original spelling.***PaludinaSenegalensis* Morelet, 1860.

**Type locality.** “Habitat circa Podor in paludibus” [Senegal, Podor].

### *Paramelaniaservainiana* Bourguignat, 1885

**Original reference.**Bourguignat (1885a: 83–84).

**Type locality.** “Sur la plage Pambété” [Zambia, beach of Pambété, Lake Tanganyika].

### *Hauttecoeuriaservainiana* Bourguignat, 1888

**Original reference.**Bourguignat (1888: 21–22, pl. 8, figs 18, 19).

**Original spelling.***Hauttecœuria Servainiana* Bourguignat, 1888.

**Type locality.** “Lac Tanganika” [Lake Tanganyika].

### *Paludomussiamensis* Blanford, 1903

**Original reference.**Blanford (1903: 283–284, pl. 8, fig. 3).

**Original spelling.***PaludomusSiamensis* Blanford, 1903.

**Type locality.** “Siam, in valle superiore Menam fluminis” [Thailand, upper Chao Phraya Valley].

### *Paludomusconicasibsaugorensis* Nevill, 1884

**Original reference.**Nevill (1884: 288).

**Original spelling.**Paludomusconicavar.sibsaugorensis Nevill, 1884.

**Type locality.** “Sibsagar” [India, Assam, Sivasagar].

**Remarks.** See Remarks under *Paludomusconicacherraensis* Nevill, 1884.

### *Paludomusajanensissilhouettensis* Nevill, 1884

**Original reference.**Nevill (1884: 296).

**Original spelling.**Paludomusajanensisvar.silhouettensis Nevill, 1884.

**Type locality.** “Silhouette I., Seychelles”.

**Remarks.** See Remarks under *Paludomusconicacherraensis* Nevill, 1884.

### *Tanaliasimilis* Layard, 1855

**Original reference.**Layard (1855: 92).

**Type locality.** “A mountain torrent at Kandangamoa, near Ratnapoora” [Sri Lanka, Ratnapura, Kandangamoa].

### *Hauttecoeuriasingularis* Bourguignat, 1885

**Original reference.**Bourguignat (1885a: 52–53).

**Original spelling.***Hauttecœuria singularis* Bourguignat, 1885.

**Type locality.** “Près de Pambété” [Zambia, near Pambété, Lake Tanganyika].

### *Paramelaniasingularis* Bourguignat, 1888

**Original reference.**Bourguignat (1888: 35–36, pl. 15, figs 16, 17).

**Type locality.** “Lac Tanganika” [Lake Tanganyika].

### *Paludomusskinneri* Dohrn, 1857

**Original reference.**Dohrn (1857: 124–125).

**Original spelling.***PaludomusSkinneri* Dohrn, 1857.

**Type locality.** “Ceylon” [Sri Lanka].

### *Paramelaniasmithi* Bourguignat, 1888

**Original reference.**Bourguignat (1888: 37–38, pl. 16, figs 11, 12).

**Original spelling.***ParamelaniaSmithi* Bourguignat, 1888.

**Type locality.** “Lac Tanganika” [Lake Tanganyika].

### *Cleopatrasmithi* Ancey, 1906

**Original reference.**Ancey (1906a: 45–46).

**Original spelling.***CleopatraSmithi* Ancey, 1906.

**Type locality.** “River Chozi, which flows into the Chambézi, region of lake Bangwéolo, British Central Africa” [Zambia, region of Lake Bangweulu, Chozi River, tributary of Chambeshi River].

### *Stanleyasmithiana* Bourguignat, 1885

**Original reference.**Bourguignat (1885a: 88–89).

**Type locality.** “Plage de Mlilo” [Democratic Republic of the Congo, beach of Mlilo (= Moliro), Lake Tanganyika].

### *Cleopatrasoleilleti* Bourguignat, 1885

**Original reference.**Bourguignat (1885b: 28, pl. 1, fig. 1).

**Original spelling.***CleopatraSoleilleti* Bourguignat, 1885.

**Type locality.** “Lac Haoussa” [Ethiopia, Lake Awasa].

### *Paludomussolida* Dohrn, 1857

**Original reference.**Dohrn (1857: 124).

**Original spelling.***Paludomussolidus* Dohrn, 1857.

**Type locality.** “Ceylon” [Sri Lanka].

### *Hauttecoeuriasoluta* Bourguignat, 1885

**Original reference.**Bourguignat (1885a: 51–52).

**Original spelling.***Hauttecœuria soluta* Bourguignat, 1885.

**Type locality.** “Aux environs de Pambété” [Zambia, surroundings of Pambété, Lake Tanganyika].

### *Paludomussphaerica* Dohrn, 1857

**Original reference.**Dohrn (1857: 124).

**Original spelling.***Paludomus sphæricus* Dohrn, 1857.

**Type locality.** “Ceylon” [Sri Lanka].

### *Paramelaniaspinulosa* Bourguignat, 1885

**Original reference.**Bourguignat (1885a: 75–76).

**Type locality.** “Plage de Mlilo” [Democratic Republic of the Congo, beach of Mlilo (= Moliro), Lake Tanganyika].

### *Paludomusspiralis* Reeve, 1847

**Original reference.**Reeve (1847: pl. 3, fig. 15).

**Type locality.** “Streams of Ceylon” [Sri Lanka].

### *Paludomusspurca* Adams & Adams, 1854

**Original reference.**Adams and Adams (1853–1858: pl. 36, fig. 2).

**Original spelling.***Paludomusspurcus* Adams & Adams, 1854.

**Type locality.** Not given.

### *Paramelaniastanleyana* Bourguignat, 1885

**Original reference.**Bourguignat (1885a: 75).

**Type locality.** “Plages de Pambété et de Mlilo” [Zambia, beach of Pambété and Democratic Republic of the Congo, beach of Mlilo (= Moliro), Lake Tanganyika].

### *Edgarianassastappersi* Leloup, 1953 [Unavailable]

**Original reference.**Leloup (1953: 173).

**Original spelling.**Edgarianassavar.stappersi Leloup, 1953.

**Remarks.** Mentioned in the synonymy of *Edgardianassa* forme *spinulosa* as a manuscript name of Dautzenberg; unavailable (Code Art. 11.6).

### *Melaniastephanus* Benson, 1836

**Original reference.**Benson (1836b: 747).

**Original spelling.***MelaniaStephanus* Benson, 1836.

**Type locality.** From the north-east frontier of Bengal (Benson, 1836a: 350).

### † *Edgariastockleyi* Cox, 1939

**Original reference.**Cox (1939: 246, pl. 15, figs 3–5).

**Type locality.** “The Rungwa Sleeping Sickness Concentration Area, lies about eighty miles to the north-west of the present shore of the lake [Tanzania, Rungwa].

**Type stratum.** “Quaternary deposits of Lake Rukwa”.

### *Tanaliastomatodon* Benson, 1862

**Original reference.**Benson (1862: 414–415).

**Original spelling.***Tanalia* (?) *Stomatodon* Benson, 1862.

**Type locality.** “Habitat in aquis dulcibus montium prope Cottyam, regionis Travancoriæ” [India, Kerala, Kottayam].

### *Paludomusstraeleni* Leloup, 1953

**Original reference.**Leloup (1953: 14–15, 114, 130–131, 133–138, pl. 2, fig. 16, figs. 4, 57T, 70, 72DD, 73A–C).

**Type locality.** “Récoltes de la Mission hydrobiologique belge. N^o^ 38 — Moba, chalutage le long de la côte Nord du village, — 20 m, vase noire (I). N^o^ 43 — Moba, au large, petite drague, vase. N^o^ 49 — Baie de Toa, — 15 m, petite drague, sable, cailloux (II). N^o^ 50 — Au large du cap Bwana n’denge, à 1.000 m, le long de la côte, chalutage depuis 3 milles au Nord-Ouest de la Lugumba jusque par le travers de la rivière, — 20–80 m, fonds divers, vase, rochers, coquilles et spécimens vivants. N^o^ 80 — Dans la baie de Burton, au large de la rivière Mutambala, — 40 m, chalut à panneaux, sable vaseux. N^o^ 127 — Baie d’Utinta, 45–65 m, drague à herse, sable vaseux (III). N^o^ 133 — Baie de Katibili, à 500 m de la rive, — 65–70 m, drague à herse, sable (IV). N^o^ 138 — Baie de Bracone, dans l’île Kavala; — 12 m, petite drague, sable cailloux (V). N^o^ 143 — Baie au Sud de la Malagarasi, à la pointe Sud du delta et devant la rivière. N^o^ 246 — Dans la baie de Burton, à 1 mille au large de Baraka, — 30 m, petite drague, sable. N^o^ 267 — Baie de Nyanza, par le travers, au départ, ± 60 m (VI). N^o^ 328 — Laguno de Katibili, dans le goulot, — 0,50 m, sable (VII)” and “au large de Kituta, Sud du lac (stn. 2064, — 76 m)” and “dans la baie de Sumbu (stn. 2069, — 20–70 m)” [Lake Tanganyika].

### *Paludomusstriata* Nevill, 1884 [Unavailable]

**Original reference.**Nevill (1884: 297).

**Remarks.** Published as a manuscript name under the unavailable name (introduced without description, definition, or indication) *Paludomusmicrosculpta*; unavailable (Code Art. 11.6).

### *Paludomusstriatula* Nevill, 1884

**Original reference.**Nevill (1884: 297–298).

**Type locality.** “Ceylon” [Sri Lanka].

### *Paludomussubdentata* Nevill, 1884

**Original reference.**Nevill (1884: 300).

**Original spelling.***Paludomus* [*Philopotamis*] *subdentata* Nevill, 1884.

**Type locality.** “Ceylon” [Sri Lanka].

### *Paludomussubfasciata* Martens, 1908

**Original reference.**Martens and Thiele (1908: 266–267, pl. 5, fig. 2).

**Type locality.** “Sungei Guleh, Sangkulirang” [Indonesia, Borneo, East Kalimantan].

### *Paludomusnigricanssubgranulosa* Nevill, 1884

**Original reference.**Nevill (1884: 299).

**Original spelling.***Paludomus* [*Philopotamis*] *nigricans*var.subgranulosa Nevill, 1884.

**Type locality.** “Ceylon [and] Newera Ellia” [Sri Lanka; Nuwara Eliya].

**Remarks.** See Remarks under *Paludomusconicacherraensis* Nevill, 1884.

### *Paludomusgrandidierisubmutica* Crosse & Fischer, 1878

**Original reference.**Crosse and Fischer (1878: 74, pl. 1, figs 4, 4a).

**Original spelling.***PaludomusGrandidieri* var. β *Submutica* Crosse & Fischer, 1878.

**Type locality.** “Madagascar, dans les ruisseaux de la partie orientale de l’île” [Madagascar, eastern part of the island].

### *Paludomushanleyisubpicta* Nevill, 1884

**Original reference.**Nevill (1884: 305).

**Original spelling.***Paludomus* [*Tanalia*] *hanleyi*var.subpicta Nevill, 1884.

**Type locality.** “Yatteantotte, Ceylon” [Sri Lanka].

**Remarks.** See Remarks under *Paludomusconicacherraensis* Nevill, 1884.

### *Paludomussulcata* Reeve, 1847

**Original reference.**Reeve (1847: pl. 2, fig. 8a, pl. 3, figs 8b–c).

**Original spelling.***Paludomussulcatus* Reeve, 1847.

**Type locality.** “In a mountain stream at Ratnapoora, Ceylon” [Sri Lanka, Ratnapura].

### † *Paludomussuraiensis* Gurung, 1998

**Original reference.**Gurung (1998: 19–20).

**Type locality.** “Surai Khola, western Nepal”.

**Type stratum.** “Upper part of the Surai Khola Formation, the Dobata Formation

and lower part of the Dhan Khola Formation”.

**Remarks.** See also Parmar (2013).

### *Paludomusswainsoni* Dohrn, 1857

**Original reference.**Dohrn (1857: 125).

**Original spelling.***PaludomusSwainsoni* Dohrn, 1857.

**Type locality.** “Ceylon” [Sri Lanka].

### *Paramelaniatabulata* Sowerby, 1890

**Original reference.**Sowerby (1890: fig. 8 and explication of plate).

**Type locality.** “Lake Tanganyika”.

**Remarks.** See also Verdcourt (2000).

### *Spekiazonatatanganikana* Bourguignat, 1885

**Original reference.**Bourguignat (1885a: 37).

**Original spelling.***Spekiazonata* var. *Tanganikana* Bourguignat, 1885.

**Type locality.** “Plage de Kapampa, dans le Marungu, à l’ouest du lac“ [Democratic Republic of the Congo, Marungu region, beach of Kapampa, western shore of Lake Tanganyika].

### *Horeatanganikana* Bourguignat, 1888

**Original reference.**Bourguignat (1888: 27–28, pl. 11, figs 28–29).

**Original spelling.***HoreaTanganikana* Bourguignat, 1888.

**Type locality.** “Lac Tanganika” [Lake Tanganyika].

### *Reymondiatanganikana* Ancey, 1894 [Invalid]

**Original reference.**Ancey (1894: 28).

**Remarks.** Unjustified emendation of *Reymondiatanganyicensis* Smith, 1889.

### *Melaniatanganyicensis* Smith, 1880

**Original reference.**Smith (1880b: 427).

**Type locality.** “Lake Tanganyika”.

### *Reymondiatanganyicensis* Smith, 1889

**Original reference.**Smith (1889: 175).

**Type locality.** “Lake Tanganyika”.

### *Marteliatanganyicensis* Dautzenberg, 1908

**Original reference.**Dautzenberg (1908: 329, pl. 4, figs 11–12).

**Type locality.** “À M’Pala” [Democratic Republic of the Congo, Mpala, Lake Tanganyika].

### *Hemimitratangi* Chen, 1943

**Original reference.**Chen (1943: 19–20, pl. 6, fig. 2).

**Type locality.** “Kiang-yang, northern Fukien Province” [China, Fujian].

### *Paludomustanjorica* Blanford, 1880 [Invalid]

**Original reference.**Blanford (1880: 220).

**Remarks.** Unjustified emendation of *Helixtanschaurica* Gmelin, 1791.

### *Paludomustanjoriensis* Blanford, 1863

**Original reference.**Blanford (1863: 171).

**Original spelling.***PaludomusTanjoriensis* Blanford, 1863.

**Type locality.** Not given, but by reference to Gmelin (1791: 3655): “Habitat in Coromandel aquis dulcibus” [India, Coromandel]. See also Chemnitz (1786: 174).

**Remarks.** Intended as an emendation for the unavailable name of the nominal taxon *Helixfluviatilistanschauriensis* Chemnitz, 1786 (given as *tanschauriensis* and a reference to Gmelin). Interpreted here not as an emendation but as a newly introduced name by Blanford (1863).

### *Helixtanschaurica* Gmelin, 1791

**Original reference.**Gmelin (1791: 3655).

**Original spelling.***Paludomuslanschaurica* Gmelin, 1791.

**Type locality.** “Habitat in Coromandel aquis dulcibus” [India, lives in freshwater in Coromandel]. According to Chemnitz (1786) “bey Tirutschinapalli und Tanschaur auf Coromandel” [India, Tamil Nadu, near Tiruchirappalli and Thanjavur in Coromandel].

**Remarks.** Spelled as *Helixlanschaurica* by Gmelin (1791: 3655), which is interpreted as an incorrect original spelling (Code Art. 33.5.1), as the reference to Chemnitz (1786: 175) suggests that Gmelin was aware of the correct etymology of the species epithet. The name *Helixlanschaurica* has rarely been used as the valid name of a taxon (Rees 1819; Woodarch 1820; Wodarch and Mawe 1822, 1825, 1827, 1831, 1833; Bosc 1802, 1824, 1836; Pfeiffer 1840, 1841) and to our knowledge only once after 1900 (Coan and Petit 2011), whereas *Helixtanschaurica* is comparatively frequently used (e.g., Gmelin 1792; Frauenfeld 1864 as *Helixtanschaurica*; Schmidt 1818 as *Melaniatanschaurica*; Gray 1855 as *Paludinatanschaurica*; Hanley and Theobald 1876; Preston 1915b; Seshaiya, 1929, 1934; Jacob 1959; Satyamurty 1960; Starobogatov 1970; Subba Rao and Mitra 1979; Brown 1980, 1994; Fretter 1984; Brown and Mandahl-Barth 1989; Daniel et al. 1998; Dillon 2000; Strong and Glaubrecht 2002; Thasun Amarasinghe and Krishnarajah 2009; Strong et al. 2011 as *Paludomustanschaurica*; Starmühlner 1974, 1977, 1979; Subba Rao 1989 as *Paludomustanschauricus*) and usually attributed to Gmelin (1791) (although sometimes with a wrong date). *Paludomussanschauricus* as used by Adams and Adams (1853–1854) is an incorrect subsequent spelling.

### *Helixfluviatilistanschauriensis* Chemnitz, 1786 [Unavailable]

**Original reference.**Chemnitz (1786: 174, pl. 135, 1243a–b).

**Original spelling.***HelixfluviatilisTanschauriensis* Chemnitz, 1786.

**Type locality.** “Sie ist bey Tirutschinapalli und bey Tanschaur auf Coromandel in den dortigen Bächen und süssen Wassern gefunden worden” [India, Tamil Nadu, near Tiruchirappalli and Thanjavur in Coromandel].

**Remarks.** Published in a work rejected by ICZN Direction 1 (ICZN 1987).

### *Cleopatratchadiensis* Germain, 1905

**Original reference.**Germain (1905b: 328, footnote).

**Original spelling.***CleopatraTchadiensis* Germain, 1905.

**Type locality.** “Dans le Tchad, notamment aux environs de l’archipel Kouri” [Chad/ Niger/Nigeria/Cameroon, Lake Chad, Kouri Islands].

**Remarks.**Cleopatrabulimoidesvartschadiensis as used by Kobelt (1909) is an incorrect subsequent spelling.

### *Paludomustennantii* Reeve, 1847

**Original reference.**Reeve (1847: pl. 3, figs 12a–c).

**Original spelling.***PaludomusTennantii* Reeve, 1847.

**Type locality.** “In a rocky stream flowing from Adam’s Peak, Ceylon” [Sri Lanka, near Adam’s Peak].

**Remarks.***Tanaliatennentii* in Layard (1855) and Paludomus (Tanalia) aculeatavar.tennenti in Nevill (1884) are incorrect subsequent spellings.

### *Turbonillaterebriformis* Smith, 1890

**Original reference.**Smith (1890: 95).

**Original spelling.**Turbonilla?terebriformis Smith, 1890.

**Type locality.** “Lake Tanganyika”.

### *Philopotamisthwaitesii* Layard, 1855

**Original reference.**Layard (1855: 93–94).

**Original spelling.***Paludomus Thwaitsii* Layard, 1855.

**Type locality.** “Weyweldenia, a rocky rivulet [and] at Kandanganoa and in the Calloo ganga” [Sri Lanka].

**Remarks.**Paludomus (Tanalia) aculeatavar.thwaitesi is an incorrect subsequent spelling by Nevill (1884).

### *Limnotrochusthomsoni* Smith, 1880

**Original reference.**Smith (1880b: 425).

**Original spelling.***LimnotrochusThomsoni* Smith, 1880.

**Type locality.** “Lake Tanganyika”.

### *Paramelaniatiarella* Martens, 1895

**Original reference.**Martens (1895: 187).

**Original spelling.**Paramelania (Edgaria) tiarella Martens, 1895.

**Type locality.** “Tanganyika” [Lake Tanganyika].

### *Paramelaniatimida* Bourguignat, 1888

**Original reference.**Bourguignat (1888: 35–36, pl. 15, figs 24–25).

**Type locality.** “Lac Tanganika” [Lake Tanganyika].

### *Pseudocleopatratogoensis* Thiele, 1928

**Original reference.**Thiele (1928: 394–395, 400, 402, textfig. 58, pl. 8, figs 18, 18a).

**Type locality.** “Fluß Volta bei Apaso (Togo)” [Ghana, Apaso, now inundated by Lake Volta].

### *Paludomustorrenticola* Dohrn, 1858

**Original reference.**Dohrn (1858: 536).

**Original spelling.**Paludomus (Tanalia) torrenticola Dohrn, 1858.

**Type locality.** “Inhabitants of mountain-streams in Ceylon” [Sri Lanka].

### *Cleopatratrabonjiensis* Smith, 1882

**Original reference.**Smith (1882: 384, pl. 22, figs 10–11).

**Type locality.** “A small lake at Trabonjy, in the north-west central part of the island” [Madagascar, Trabonjy].

### *Paludomustravancorica* Blanford, 1880

**Original reference.**Blanford (1880: 219–220, pl. 2, fig. 22).

**Type locality.** “In Travancor (p. 219) and more specifically: in streams traversing the plains between Trevandrum and the foot of the Aghastyamali hill (p. 220)” [India, Kerala].

### *Paludomustrifasciata* Reeve, 1852

**Original reference.**Reeve (1852: 126).

**Original spelling.***Paludomustrifasciatus* Reeve, 1852.

**Type locality.** “Branch of the Ganges” [India/Bangladesh].

### *Bithyniatrifasciata* Frauenfeld, 1864 [Unavailable]

**Original reference.**Frauenfeld (1864: 583, 655).

**Original spelling.***Bythiniatrifasciata* Frauenfeld, 1864.

**Type locality.** Not given.

**Remarks.** Attributed to Parreyss; published in the synonymy of *Bithyniabulimoides* (Olivier, 1804). Also mentioned by Martens (1865: 203) in the synonymy of Paludina (Cleopatra) bulimoides. Treated as an available name, *Paludinatrifasciata*, of a taxon by Paetel (1873: 64). However, the name was not adopted, i.e. the unavailable name was not used as the valid name of a taxon in a way which establishes it as a new name with its own authorship and date in accordance with the Code (Art. 12.1), and therefore was not made available under the Code (Art. 11.6.1) by Paetel (1873).

### *Edgariatrochlearis* Leloup, 1953 [Unavailable]

**Original reference.**Leloup (1953: 166).

**Remarks.** Mentioned in the synonymy of *Edgardianassa* forme *typica*; unavailable (Code Art. 11.6).

### *Marteliatanganyicensistypica* Leloup, 1953 [Invalid]

**Original reference.**Leloup (1953: 107, 111, 113, 116, 146, pl. 2, fig2, figs 53, 56, 80–81).

**Original spelling.***Paludomustanganyicensis* forme *typica* Leloup, 1953.

**Remarks.** Name for the nominal forme.

### *Bridouxiagirauditypica* Leloup, 1953 [Invalid]

**Original reference.**Leloup (1953: 115, 130, 132, 136, 140–144, pl. 13, figs 6A–B, figs 57V–W, 70, 72J, 75, 78A–B, E).

**Original spelling.***Bridouxiagiraudi* forme *typica* Leloup, 1953.

**Remarks.** Name for the nominal forme.

### *Edgarianassatypica* Leloup, 1953 [Invalid]

**Original reference.**Leloup (1953: 130, 132, 154–158, 164–167, pl. 7, fig. 1, pl. 8, fig. 1, pl. 9, fig. 2, figs 71, 72N, 86).

**Original spelling.***Edgarianassa* forme *typica* Leloup, 1953.

**Remarks.** Name for the nominal forme.

### *Paludomusundata* Reeve, 1847

**Original reference.**Reeve (1847: pl. 1, figs 2a–b).

**Original spelling.***Paludomusundatus* Reeve, 1847.

**Type locality.** “In rapids flowing from Adam’s Peak, Ceylon” [Sri Lanka, near Adam’s Peak].

### *Cleopatrapirothiunicarinata* Martens, 1886

**Original reference.**Martens (1886: 126–127).

**Original spelling.**CleopatraPirothivar.unicarinata Martens, 1886.

**Type locality.** “Aegypten, Fajum-Becken unweit Adue über den Bats am Bahndamm in 17 Meter Seehöhe” [Egypt, Fayum Basin, close to Adue above the Bats near the train embankment, 17 m. a.s.l.].

### *Syrnolopsisminutaunicarinata* Ancey, 1906

**Original reference.**Ancey (1906b: 255, 267).

**Original spelling.**Syrnolopsisminutaf.unicarinata Ancey, 1906.

**Type locality.** “Lac Tanganika, côte orientale” [Tanzania/Burundi, eastern shore of Lake Tanganyika].

### *Cleopatrabulimoidesunilirata* Germain, 1911

**Original reference.**Germain (1911: 199, pl. 2, figs 22–24).

**Original spelling.**Cleopatrabulimoidesvar.unilirata Germain, 1911.

**Type locality.** “Zone sableuse entre Bosso et l’embouchure de la Komadougou, dans le Tchad” [Between Nigeria, Bosso and Nigeria/Niger, mouth of Komadougou River near Lake Chad], “Kélékorarom, dans le Tchad” [Lake Chad, Kélékorarom Island] and “Intérieur du Tchad, à 30 kilomètres du bord Ouest” [Lake Chad, 30 km from its western shore].

### *Spekiazonataunisulcata* Bourguignat, 1890

**Original reference.**Bourguignat (1890: 64).

**Original spelling.***Spekiazonata* var. b *Unisulcata* Bourguignat, 1890.

**Type locality.** “Plage de Pambété” [Zambia, beach of Pambété, Lake Tanganyika].

### † *Cleopatravanloockei* Van Damme & Pickford, 2003

**Original reference.**Van Damme and Pickford (2003: 11, 52–53, fig. 37).

**Type locality.** “Nkondo locality NK 76, Lake Albert, Uganda”.

**Type stratum.** “Nkondo Formation, Molluscan Association G3b, Terminal Miocene -Early Pliocene”.

### *Nassopsisvariabilis* Martel & Dautzenberg, 1899

**Original reference.**Martel and Dautzenberg (1899: 174–175, pl. 8, figs 16–17).

**Type locality.** “Lac Tanganyika” [Lake Tanganyika].

### *Paludomusventricosula* Nevill, 1884

**Original reference.**Nevill (1884: 299–300).

**Original spelling.***Paludomus* [*Philopotamis*] *ventriculosa* Nevill, 1884.

**Type locality.** “Ceylon [and] Kandapanne Ella, 4 Corles [and] Hackalle, Ceylon” [Sri Lanka].

### *Paramelaniavenusta* Bourguignat, 1888

**Original reference.**Bourguignat (1888: 37–38, pl. 16, figs 13–14).

**Type locality.** “Lac Tanganika” [Lake Tanganyika].

### *Cleopatraverreauxi* Pallary, 1909 [Invalid]

**Original reference.**Pallary (1909: 64).

**Original spelling.***CleopatraVerreauxi* Pallary, 1909.

**Remarks.** Unjustified emendation of *Bithyniaverrauxiana* Bourguignat, 1856; the name as introduced by Bourguignat (1856) was given in the synonymy including a reference to the work (Code Art. 33.2.1).

### *Bithyniaverreauxiana* Bourguignat, 1856

**Original reference.**Bourguignat (1856: 241–242).

**Original spelling.***Bithinia Verreauxiana* Bourguignat, 1856.

**Type locality.** “Le Nil, en Égypte” [Egypt, Nile River].

**Remarks.***Cleopatraverreauxi* as used by Kobelt (1909) is apparently an incorrect subsequent spelling, whereas *Cleopatraverreauxi* Pallary, 1909 is interpreted as an unjustified emendation.

### *Bithyniavexillata* Frauenfeld, 1864 [Unavailable]

**Original reference.**Frauenfeld (1864: 583, 659).

**Original spelling.***Bythiniavexillata* Frauenfeld, 1864.

**Type locality.** Not given.

**Remarks.** Attributed to Parreyss; published in the synonymy of *Bithyniabulimoides*. Also mentioned by Martens (1865: 203) in the synonymy of Paludina (Cleopatra) bulimoides. Treated as an available name, *Paludinavexillata*, of a taxon by Paetel (1873: 64). However, the name was not adopted, i.e. the unavailable name was not used as the valid name of a taxon in a way which establishes it as a new name with its own authorship and date in accordance with the Code (Art. 12.1), and therefore was not made available under the Code (Art. 11.6.1) by Paetel (1873).

### *Bridouxiavilleserriana* Bourguignat, 1885

**Original reference.**Bourguignat (1885a: 30–31).

**Type locality.** “Sur la plage de Kapampa” [Democratic Republic of the Congo, beach of Kapampa, western shore of Lake Tanganyika].

### *Tanaliaviolacea* Layard, 1855

**Original reference.**Layard (1855: 92).

**Type locality.** “A small mountain torrent in a dense forest between Gillymalle and Pallabaddoola, towards Adam’s Peak, Ceylon” [Sri Lanka].

### *Pseudocleopatravoltana* Mandahl-Barth, 1973

**Original reference.**Mandahl-Barth (1973: 279–281, fig. 3a, h).

**Type locality.** “River Volta at Daboya” [Ghana, Daboya, River Volta].

### *Paludomusvondenbuschianus* Arifin, 2017 [Unavailable]

**Original reference.**Arifin (2017: 110, 111).

**Remarks.** Nomen nudum, introduced without definition or description (Code Art. 13.1.1) or bibliographic reference to a definition or description (Code Art. 13.1.2); also not explicitly indicated as intentionally new (Code Art. 16.1).

### † *Pseudocleopatrawamalai* Van Damme & Pickford, 2003

**Original reference.**Van Damme and Pickford (2003: 10, 13, 57, 69–70, 76, 79, fig. 45).

**Type locality.** “Nkondo locality NK 84, L. Albert, Uganda”.

**Type stratum.** “Lusso Formation (Biozone I), Upper Nyaburogo and Warwire Formations, Molluscan Associations G4 and G5a, Early to Late Pliocene”.

### *Cleopatrabulimoideswelwitschi* Martens, 1897

**Original reference.**Martens (1897: 185).

**Original spelling.**Cleopatrabulimoidesvar.welwitschi Martens, 1897.

**Type locality.** “Angola, in einem salzhaltigen Bach bei Dungo im Distrikt Pungo-Adongo (am Cuanza)” [Angola, Dungo, Pungo, Cuanza].

### *Vinunduwestae* Michel, 2004

**Original reference.**Michel (2004: 4–11, figs 1A–D, 2A–I, 3A–C, E, 4B, C, 5A, B, 6C–F, 7A, B, 8A, 9).

**Type locality.** “Burundi, kilometre marker 29.8 south of Bujumbura, first major rocky shoreline, coordinates –3.640, 29.361 or 3°38'25"S, 29°21'40"E, 20 m water depth. Stromatolite-covered rocks and boulders”.

**Remarks.** The name was first introduced by West et al. (2003: 77). However, the work includes a disclaimer stating “This publication is not deemed to be valid for formal taxonomic/nomenclatural purposes”.

### † *Pseudocleopatrawilsoni* Van Damme & Pickford, 2003

**Original reference.**Van Damme and Pickford (2003: 10, 59, fig. 48).

**Type locality.** “Nkondo locality NK 126, Lake Albert, Uganda”.

**Type stratum.** “Warwire Formation, Molluscan Association G4, Early Pliocene”.

### *Paludomusyunnanensis* Cheng, 1937

**Original reference.** Cheng (1937: 445–447, figs 1.2, 2.2).

**Original spelling.**Paludomus (Hemimitra) yunnanensis Cheng, 1937.

**Type locality.** “Yangtze River, near Shiikiang, Yunnan Province, China”.

### *Melaniazanguebarensis* Petit de la Saussaye, 1851

**Original reference.**Petit de la Saussaye (1851: 263–264, pl. 7, fig. 1).

**Original spelling.***MelaniaZanguebarensis* Petit de la Saussaye, 1851.

**Type locality.** “Zanzibar, côte de Zanguebar (Afrque orientale)” [Tanzania, Zanzibar].

### *Cleopatrazanguebarica* Bourguignat, 1889

**Original reference.**Bourguignat (1889: 164–165).

**Original spelling.***CleopatraZanguebarica* Bourguignat, 1889.

**Type locality.** “Cette coquille zanzibarienne parait commune dans les cours d’eau de la vallée du Vouami” [Tanzania, Zanzibar, Vouami Valley].

### *Melaniazeylanica* Lea & Lea, 1851

**Original reference.**Lea and Lea (1851: 194–195).

**Type locality.** “Seychelles and Ceylon” [Seychelles; Sri Lanka].

**Remarks.***Paludomusceylanicus* is a subsequent misspelling by Issel (1874).

### *Meloniazonata* von dem Busch, 1842

**Original reference.**Philippi (1842–1845: 3, pl. 1, fig. 12).

**Type locality.** “Bengalia” [India/Bangladesh].

**Remarks.** The entire text with descriptions of von dem Busch’s new *Melania* names was attributed to von dem Busch on p. 4 of Philippi’s (1842–1845) work.

### *Cleopatrabroeckizonata* Putzeys, 1899

**Original reference.**Putzeys (1899: 60).

**Original spelling.**CleopatraBroeckivar.zonata Putzeys, 1899.

**Type locality.** “De la rivière Aruwimi, affluent du Congo” [Democratic Republic of the Congo, Aruwimi River].

### *Lithoglyphuszonatus* Woodward, 1859

**Original reference.**Woodward (1859: 349, pl. 47, fig. 3).

**Type locality.** “Lake Tanganyika in Central Africa”.
